# Supplementary material for: Calibration and analysis of genome-based models for microbial ecology
Source: eLife. 2015 Oct 16;4:e08208. doi: 10.7554/eLife.08208 (PMC4608356; doi:10.7554/eLife.08208)
Supplement: Supplementary file 1. — A thorough user manual for MCM, including an in-depth description of MCM's mathematical framework and step-by-step examples. The latest version can also be retrieved at http://www.zoology.ubc.ca/MCM. DOI: http://dx.doi.org/10.7554/eLife.08208.013 [file elife08208s001.pdf]

MCM

Microbial Community Modeler 1.3
- User Manual -

Stilianos Louca
University of British Columbia, Canada

August 1, 2015

Contents

1 Introduction **4**
1.1 Overview . . . 4
1.2 General model structure . . . 6
1.3 Mathematical model . . . 7

2 Introductory example **10**
2.1 Constructing a microbial community model . . . 10
2.2 Running a single simulation . . . 13
2.3 Uncertainty analysis . . . 15
2.4 Sensitivity analysis . . . 17
2.5 Comparing and fitting the model to data . . . 18
2.6 Fitting the model using arbitrary composite data . . . 21
2.7 Including environmental stochasticity . . . 23
2.8 Including bacteriophages . . . 26
2.9 Environmental (abiotic) reactions . . . 28

3 Installation **33**
3.1 System requirements and dependencies . . . 33
3.2 3rd party components . . . 33
3.3 Installing MCM . . . 34
3.4 Compiling MCM from the sources . . . 34
3.5 Plotting . . . 35

4 MCM control **36**
4.1 The MCM command line . . . 36
4.2 Reading syntax overviews . . . 38
4.3 MCM command line macros . . . 38
4.4 MCM scripts . . . 39

5 Defining a microbial community model **40**
5.1 Environmental variables . . . 40
5.1.1 Introduction . . . 40
5.1.2 Specifying environmental variables . . . 40
5.1.3 Dynamics . . . 41
5.1.4 Temperature and pH as special cases . . . 43
5.2 Metabolites . . . 44
5.2.1 Metabolite transport kinetics . . . 45
5.2.2 Environmental metabolite dynamics . . . 46
5.3 Reactions . . . 48
5.3.1 Chemical equation . . . 48
5.3.2 Gibbs free energy . . . 49
5.3.3 Reaction rate limits . . . 49
5.3.4 Objective coefficients . . . 50
5.3.5 Environmental rates . . . 50
5.4 Species . . . 51
5.4.1 Cell life time . . . 52
5.4.2 Metabolic modulation . . . 53
5.4.3 Population growth . . . 53
5.4.4 The role of cell mass . . . 54
5.4.5 Compartmentalization within cells . . . 54
5.4.6 Species-specialization . . . 54
5.4.7 Enforcing individual reactions . . . 55
5.5 Observables . . . 56
5.5.1 Introduction . . . 56
5.5.2 Defining observables . . . 56

5.6 Focals . . . 58
5.7 Symbolic model parameters . . . 59
5.8 Macros . . . 60
5.9 Model templates . . . 61
5.10 Periodic dilutions . . . 63
5.11 Compartmentalized ecosystem models . . . 63
5.12 Non-stationary cell models . . . 65
5.13 General notes . . . 67
5.13.1 Naming rules . . . 67
5.13.2 Custom functions . . . 67
5.13.3 Random generators . . . 68
5.13.4 Physical units . . . 68
5.14 `micog`: Converting conventional FBA models into MC models . . . 69
5.14.1 Example 1 . . . 70
5.14.2 Example 2 . . . 71

6 Running a single MCM simulation 72

7 Comparing and calibrating models to data 74
7.1 Introduction . . . 74
7.2 Comparing simulations to data . . . 76
7.3 Estimating (fitting) unknown model parameters . . . 77

8 Sensitivity analysis 79
8.1 Sensitivity of model predictions . . . 79
8.2 Sensitivity of log-likelihoods . . . 82

9 Uncertainty analysis 83

10 Example 1 - Simulating a single-species model 84
10.1 Configuring the MC model . . . 84
10.2 Setting up the MCM control script . . . 86
10.3 Running the simulation . . . 86

11 Example 2 - Sensitivity and uncertainty analysis of the *E. coli* model 88
11.1 Defining the symbolic parameters . . . 89
11.2 Sensitivity analysis . . . 89
11.3 Uncertainty analysis . . . 92

12 Example 3 - Comparing a two-species model to data 94
12.1 Configuring the MC model . . . 94
12.2 Running a single simulation using default parameters . . . 96
12.3 Comparing the simulation to data . . . 100
12.4 Local sensitivity analysis of the log-likelihoods . . . 101
12.5 Global sensitivity analysis of the log-likelihoods . . . 103
12.6 Calibrating parameters to data . . . 104
12.7 Calibrating measurement units . . . 106

13 MCM output files (reference list) 108

14 FAQ 111
14.1 Installation and compatibility . . . 111
14.1.1 I get an error that `yacc` was not found when compiling . . . 111
14.1.2 I get an error that `lex` was not found when compiling . . . 111
14.1.3 When I compile I get an error that `omp.h` was not found . . . 112
14.1.4 I get an error that the `libgomp` library was not found . . . 112
14.1.5 Heatmap PDF plots appear blurred . . . 112
14.1.6 MCM doesn't generate any plots . . . 112
14.1.7 MCM hangs during a simulation for no apparent reason . . . 113

14.1.8 Are there any risks associated with using MCM? . . . 113
14.1.9 Will MCM run on Windows? . . . 113
14.2 Models . . . 113
14.2.1 How do I include gene regulation? . . . 113
14.2.2 MCM falsely predicts zero cell metabolism . . . 113
14.2.3 My organism should perform reaction X, but FBA does not predict it . . . 114
14.2.4 What if an FBA problem has no solution or a negative optimum? . . . 114
14.2.5 Can I use different error models for different variables? . . . 114
14.2.6 How do I keep track of species-specific activity? . . . 114
14.3 Control . . . 115
14.3.1 What's a flag or a control variable and how do I use them? . . . 115
14.3.2 Can I analyze or vizualize MC models using 3rd party tools? . . . 115
14.3.3 How can I improve the quality of plots? . . . 115
14.3.4 Can I change the appearance of heatmaps? . . . 115
14.3.5 Some simulations just hang on one time point for ever . . . 116
14.3.6 What format should my time series data files be in? . . . 116
14.3.7 Can I simulate multiple model variants at once? . . . 117
14.3.8 Can I limit the statistical analysis to a specific time interval? . . . 118
14.3.9 How do I exactly repeat a stochastic simulation? . . . 118
14.4 Citing and contributing . . . 118
14.4.1 How do I cite MCM in my work? . . . 118
14.4.2 How can I thank the creators of MCM? . . . 119
14.4.3 How can I contribute to MCM's improvement? . . . 119

15 Disclaimer and license agreement **119**

1 Introduction

1.1 Overview

MCM (Microbial Community Modeler) is a computational tool for modeling the metabolism and population dynamics of multiple unicellular organisms in a realistic environmental context. Cells interact with a dynamical environment in which metabolite concentrations and other environmental variables influence, and are influenced by, microbial metabolism. For example, cells can excrete inhibitory toxins, deplete oxygen by respiration or alter pH by lactate fermentation.

The underlying concept is known as Dynamic Flux Balance Analysis (DFBA) [93, 49, 10, 31]. In DFBA, individual cell metabolism is modeled using conventional FBA [83, 67, 57], and exported or absorbed metabolites are added to or removed from a common (*environmental*) metabolite pool available to all cell species. Environmental metabolite concentrations, in turn, influence metabolite uptake kinetics by individual cells. The model framework is dynamical because cell concentrations and metabolite concentrations can both change with time, and the rate of change of each cell concentration or metabolite concentration can depend on the current cell concentrations and metabolite concentrations. While FBA has long been used to understand individual cellular metabolism [93, 21, 38, 20, 87, 44], recent work has shown that FBA can in fact be scaled up to entire microbial communities to understand their distributed metabolic activity [87, 44, 10, 31].

MCM allows the specification of almost arbitrary microbial community (MC) models within the DFBA model framework (see sections 1.2 and 1.3 for a detailed description of the framework). In the simplest case, an MC model is defined by (i) a set of metabolites, (ii) a set of (reversible or irreversible) chemical reactions between any of the metabolites and (iii) a set of cell species, each being able to perform any subset of reactions and exchange any subset of metabolites with its environment. Optionally, an additional set of environmental variables (e.g. light intensity, temperature or pH) can be included in the model, for example to model light-limitation of photosynthesis or increased cell mortality at high temperatures.

MC models are highly configurable and can accommodate complex microbial communities with several environmental variables, large cell-metabolic networks and complex metabolite exchange kinetics. For example, environmental variables might be given as explicit functions of time, stochastic processes or interpolated from available data. Alternatively, environmental variables can be dynamic, for example with a rate of change depending on metabolite concentrations. Constraints for reaction rates and metabolite exchange rates can depend in an arbitrary manner on any metabolite concentrations and environmental variables. Metabolite concentrations can be explicit functions of time and environmental variables, interpolated from available data or depend dynamically on microbial export, biomass recycling and other measured environmental fluxes. Cell models can include dynamical internal variables that influence, and are influenced by, cell metabolism, thus allowing for so called regulatory FBA approaches [14, 13].

MCM keeps track of a multitude of output variables, such as cell concentrations (dead or alive), gene concentrations (as part of dead or living cells), cell-specific or community-wide reaction rates, metabolite concentrations and cell-specific or community-wide metabolite uptake/export rates. Apart from providing a powerful tool for simulating complex and realistic microbial communities, MCM supports:

(i) local and global sensitivity analysis with respect to any set of numerical model parameters,

(ii) uncertainty analysis in the presence of random model parameters or stochastic dynamics,

(iii) quantitative comparison and statistical evaluation against time series data,

(iv) maximum-likelihood estimation (*fitting*) of any set of numerical model parameters and their confidence intervals using available data, and

(v) maximum-likelihood estimation of unknown data units (calibration).

MCM can be used to estimate and analyze the effects of poorly quantified cell-metabolic, cell-physiological and even environmental parameters using a multitude of temporal data, ranging from chemical concentration profiles, reaction rate measurements to optical cell concentrations and metagenomics. Because MCM can estimate unknown measurement units, raw uncalibrated data (e.g. optical densities with no calibration to CFUs) can also be used for comparison and parameter fitting. In practice, model calibra-

tion becomes analogous to coefficient estimation in conventional multivariate regression. Fig. 1 gives an overview of MCM's working principle.

While MCM is designed for genome-based metabolic models, it can also accommodate conventional functional group models. In these models, different ecological functions such as primary production, heterotrophy or nitrification are performed by distinct populations whose metabolic activity is determined, for example, by Michaelis-Menten kinetics and whose growth is described by simple substrate-biomass yield factors [35, 69]. Hence, natural microbial communities could be modeled even if annotated genomes are not available for each member species. MCM's potential applicability ranges from the gut microbiome [42], soil or groundwater microbial communities [97, 78] to marine oil spills [74], laboratory cultures [47] and bioreactors [45, 48].

MCM is designed to be primarily controlled via the command line through custom scripts, i.e. plain text files containing a set of special MCM commands. This facilitates archiving, task automation, high-throughput execution of multiple simulations and incorporation into other computational pipelines (e.g. through automatic MCM script generation). MCM also includes a python script (`micog.py`) for the automated conversion of multiple FBA models in SBML file format, such as generated by the Model SEED pipeline [32], into an MC model suitable for MCM. On the other hand, MCM produces output in a multitude of file formats, ranging from raw data files, PDF and SVG figures, gnuplot plotting scripts [96] to FBA models as SBML files [37] and GEXF graph files [3].

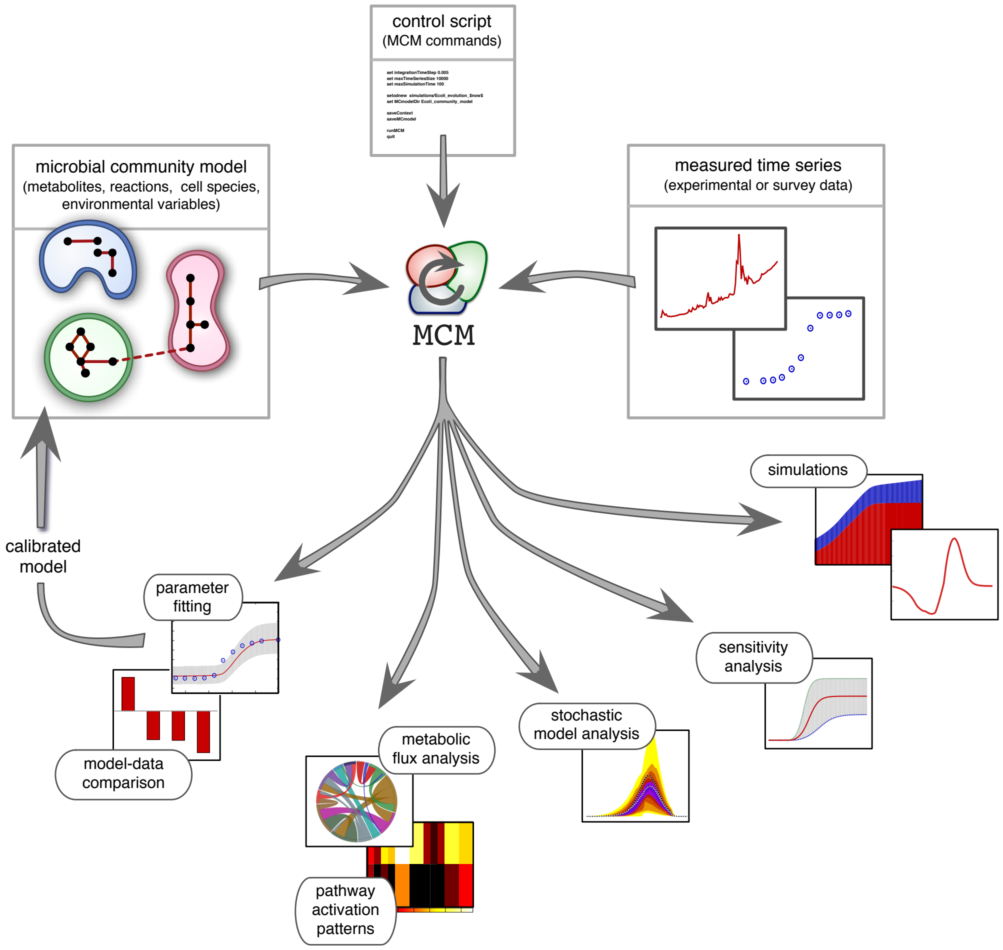

Figure 1: Overview of MCM's working principle and functionalities: A microbial community model is specified using human-readable configuration files, and defined in terms of metabolites, reactions, the metabolic potential of cell species and any additional environmental variables. Models with multiple ecosystem compartments are also possible. A script with MCM commands controls the analysis of the model and, if needed, its calibration using available time series data. The calibrated model can also be used to create new, more complex models.

This document is organized as follows: The underlying model framework is described in detail in sections

1.2 and 1.3. Section 2 provides a step-by-step introductory example that demonstrates MCM's working principles. The only prerequisites for this example are sections 1.2 and 1.3 and ideally a functioning installation of MCM. Section 3 contains details on how to install MCM.

Sections 4, 5, 6, 7, 8 and 9 serve as a detailed documentation of MCM: Section 4 explains the MCM command line interface and MCM scripts. Section 5 explains how to configure your own cell metabolic models and microbial communities from scratch. This is arguably the most complicated and most important section: The full breadth of MC model configurability is revealed here. Sections 6, 7, 8 and 9 explain MCM's main computational functions (running simulations, data comparisons and parameter fitting, sensitivity analysis, uncertainty analysis).

Sections 10, 11 and 12 guide the reader through three realistic examples using published cell-metabolic models, from the point of configuring the MC model all the way to its computational analysis and interpretation. If you are serious about using MCM, you should work through and understand these examples. It will be useful if you have skimmed through the preceding documentation before proceeding to these somewhat advanced examples.

Section 13 serves as a reference of all major MCM output files. Section 14 contains a list of frequently asked questions and issues that you are likely to run into. Finally, section 15 lists our disclaimer and license agreement (you must read this!).

1.2 General model structure

The model framework is formulated as a combination of differential equations and optimization problems. The model considers the population dynamics of S unicellular species, the concentrations of M chemical substances (*metabolites*) in the environment and the per-cell rates of R biologically catalyzed reactions involving these metabolites. Each reaction is formally identified with a unique gene. Each species is characterized by its *metabolic potential*, that is, the subset of reactions it can catalyze as well as any metabolite transport mechanisms available. The environment is assumed to be well mixed (although compartmentalized ecosystem models are also possible, see section 5.11). Optionally, the microbial community's dynamics can be influenced by additional environmental variables (such as temperature or light intensity), which might be, for example, deterministic functions of time or themselves depend on other environmental variables.

At any point in time, the reaction rates and metabolite exchange rates (i.e. *metabolism*) of each cell is assumed to depend only on its metabolic potential, metabolite concentrations and environmental variables[1]. A commonly used mathematical framework for predicting cell metabolism based on environmental nutrient concentrations and metabolic potential is *Flux Balance Analysis* (FBA) [83, 67, 57]. In this framework, cell metabolism is assumed to be regulated in such a way that some objective function, commonly identified with biomass synthesis [93, 38, 25], is maximized. The latter is assumed to be a linear function of reaction rates and/or metabolite exchange rates. The chemical state of cells is assumed to be at dynamic equilibrium, that is, metabolite concentrations within individual cells do not change with time. This assumption of *balanced fluxes* leads to stoichiometric constraints that need to be satisfied for any particular combination of intracellular reaction rates. Reaction rates are limited due to finite enzyme capacities, but might also be limited by inhibitors or toxins in the environment [2] or environmental conditions like temperature and light. For example, anammox (the oxidation of ammonium with nitrite) is obligately anaerobic, i.e., is inhibited by oxygen [95]. Uptake/export rate limits generally depend on environmental nutrient concentrations, due to finite diffusion rates and limited transmembrane transporter efficiency. For example, nutrient uptake rates by phytoplankton are often approximated by linear or Monod-like functions of nutrient concentration [22, 54]. Taken together, cell-metabolic potential, stoichiometric consistency, reaction rate limitations and transport rate limitations define the constraints for a linear optimization problem for each cell species and at each time point.

The assumption of individual cells optimizing biomass synthesis, subject to environmental and physiological constraints, is rooted in the idea that evolution has shaped regulatory mechanisms of unicellular organisms in such a way that they strive for maximum growth whenever possible. This conclusion is less valid for genetically engineered organisms or those exposed to environments that are radically different

[1] This ignores the role of a cell's life history, differences between cell cycle stages or stochastic effects.

from the environments that shaped their evolution [83]. Despite its limitations, FBA has contributed to the understanding of several genome-scale metabolic networks [93, 20, 44], metabolic interactions between cells [26, 10, 31] and the outcome of microbial evolution experiments with directional selection [38, 30], and provides a generic, intuitive starting point for modeling cellular metabolism.

Through their metabolism, cells act as sinks and sources of metabolites in the environmental pool. The total metabolite uptake and export rates across all species define an out- and in-flux into the shared metabolite pool. Other environmental fluxes (e.g. due to sedimentation or precipitation), as well as metabolite recycling due to cell death, can be additional terms in the differential equations for the environmental metabolite concentrations. Because metabolites can be used or produced by several cell species, the environmental metabolite pool provides the interaction hub for species with shared metabolic pathways [81].

The following section describes the MCM model framework in more mathematical detail. A rough understanding of the included mechanisms is required for the configuration and interpretation of microbial community models in MCM.

1.3 Mathematical model

Let $E_1, E_2, ..$ be any independent environmental variables as functions or stochastic processes of time, summarized as a vector $\mathbf{E}$. Let $C_1, .., C_M$ be the concentrations of all considered metabolites in the environmental pool, summarized as a vector $\mathbf{C} \in \mathbb{R}^M$. Similarly, let $\mathbf{N} = (N_1, .., N_S) \in \mathbb{R}^S$ be the concentrations of individual cell species. Each species s is described by a subset of available genes (i.e. the ability to perform certain reactions), as well as a list of metabolite uptake or export mechanisms (collectively referred to as its *metabolic potential*).

Technical note: MCM distinguishes between *active* and *passive* metabolite uptake/export mechanisms, for example representing transport membrane proteins and diffusion, respectively. If a cell lacks an active transport mechanism for a particular metabolite, its transport is constrained by the *passive* transport rate limits for that metabolite.

Let $\mathbb{S} \in \mathbb{R}^{M \times R}$ be the stoichiometric matrix for all possible metabolic reactions, where $\mathbb{S}_{mr}$ is the stoichiometric coefficient for metabolite m in reaction r. For example, the reaction

$$C_6H_{12}O_6 + 6O_2 + 30\mathrm{ADP} \longrightarrow 6CO_2 + 6H_2O + 30\mathrm{ATP} \tag{1}$$

might be formally associated with the presence of the cytochrome c oxidase operon, and its stoichiometric coefficients are -1, -6, -30, $+6$, $+6$ and $+30$ for the compounds $C_6H_{12}O_6$, O_2, ADP, CO_2, H_2O and ATP, respectively. Let H_{rs} be the rate of reaction r within cells of species s. For the reaction in Eq. (1), a rate of 1 mol/(cell × day) means one mole molecules $C_6H_{12}O_6$ are being catabolized per day and per cell. Let $\mathbf{F}_s \in \mathbb{R}^M$ be the corresponding net metabolite fluxes across the cell membrane, from the environment to the cell's interior, that is, $F_{m,s}$ is the net uptake rate of metabolite m by species s. The objective function (i.e. biomass production rate) is assumed to be a linear function of the species-specific reaction rates and metabolite uptake/export rates [73, 39]: Each reaction r contributes to biomass production by a constant proportionality factor Z_r; similarly, the uptake (or export) of a metabolite m contributes to the objective function by a factor $Z_{m,\mathrm{u}}$ (or $Z_{m,\mathrm{e}}$) so that the total biomass production rate per cell is

$$\mathcal{B} = \mathbf{Z}^{\mathrm{T}}\mathbf{H}_s + \mathbf{Z}_{\mathrm{u}}^{\mathrm{T}}\mathbf{F}_{s,\mathrm{u}} + \mathbf{Z}_{\mathrm{e}}^{\mathrm{T}}\mathbf{F}_{s,\mathrm{e}}, \tag{2}$$

where $F_{m,s,\mathrm{u}} = F_{m,s}$ if $F_{m,s} > 0$ (and zero otherwise) and $F_{m,s,\mathrm{e}} = -F_{m,s}$ if $F_{m,s} < 0$ (and zero otherwise). The differentiation between positive and negative fluxes allows the incorporation of different *costs* associated with the uptake or export of a particular metabolite.

In the context of FBA, intracellular metabolite concentrations are assumed to be at equilibrium, which implies $\mathbf{F}_s = -\mathbb{S}\mathbf{H}_s$. In particular, the objective function in Eq. (2) is a just linear function of $\mathbf{H}_s$. For each species s, the reaction rates $\mathbf{H}_s$ are determined by optimizing the species' biomass production rate[2] $\mathcal{B}(\mathbf{H}_s)$ under the constraints imposed on $\mathbf{F}_s$ and $\mathbf{H}_s$ by the cell's metabolic potential and the

[2]Typically, a formal reaction corresponding to biomass production using known biosynthetic precursors is included in a cell's metabolic model, with reaction coefficients chosen according to known biomass stoichiometry (e.g. lipids, proteins, RNA). The coefficients Z_r are zero for all but that one reaction [92, 93, 21, 82]. Different species can have different biomass synthesis reactions.

environment[3]. For each species one thus obtains an optimization problem with linear constraints and a linear objective function, also known as a linear programming problem [41]. A global optimum to such a problem always exists, provided that a sufficiently high number of constraints is specified. Fig. 2 exemplifies the above concepts for a simple MC model.

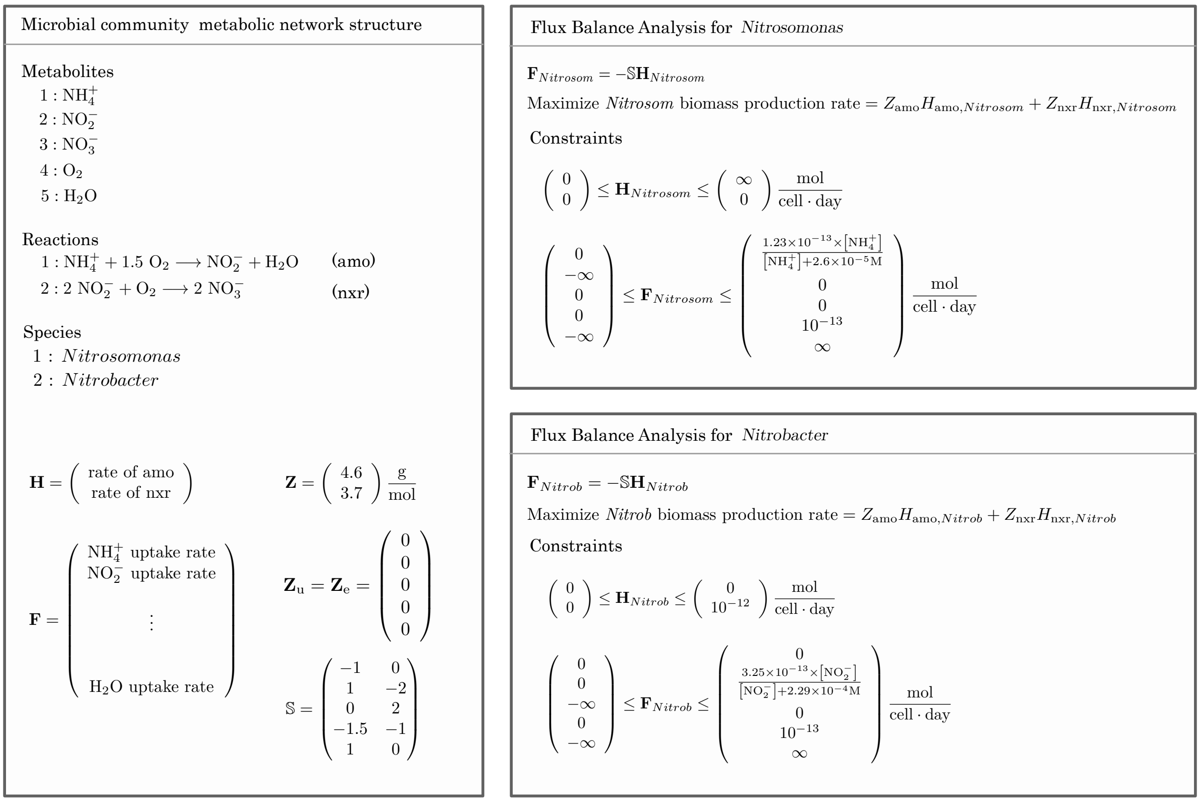

Figure 2: Overview of the metabolic network of a simple MC model comprising 5 metabolites (NH_4^+, NO_2^-, NO_3^-, O_2 and H_2O), 2 redox reactions (amo and nxr) and 2 species (*Nitrosomonas* and *Nitrobacter*). Biomass synthesis is formally associated with each of the two reactions (e.g. 4.6 g dry weight/mol for amo). amo is only performed by *Nitrosomonas* and nxr is only performed by *Nitrobacter*. Both reactions are non-reversible, amo can be performed arbitrarily fast and nxr has a maximum rate of 10^{-12} mol/(cell · day). The maximum NH_4^+ and NO_2^- uptake rates are Monod-like functions of substrate concentrations, while O_2 uptake is limited to below 1×10^{-13} mol/(cell · day). Water can be freely exchanged with the environment. *Nitrosomonas* is unable to take up NO_2^- or NO_3^- while *Nitrobacter* cannot take up NH_4^+ nor NO_3^-. Metabolite exchange, if at all possible, does not impose any explicit cost on a cell ($\mathbf{Z}_\mathrm{u} = \mathbf{Z}_\mathrm{e} = 0$).

In a nutshell, at each time point FBA defines how the metabolite concentrations $\mathbf{C}$, environmental variables $\mathbf{E}$ and cell-metabolic potential determine cell metabolism $\mathbf{H}_s(\mathbf{C}, \mathbf{E})$ as well as cell-environment exchange rates $\mathbf{F}_s(\mathbf{C}, \mathbf{E})$ for each cell species s. Calculating $\mathbf{H}_s$ and $\mathbf{F}_s$ translates to solving the underlying linear optimization problem for each cell species and at each time step. The biomass production rate $\mathcal{B}(\mathbf{H}_s)$ translates into a cell birth rate $\mathcal{B}(\mathbf{H}_s)/\mu_s$, where μ_s is the dry cell mass. Concretely,

$$\frac{dN_s}{dt} = -\frac{N_s}{\tau_s} + \frac{N_s}{\mu_s} \cdot \mathcal{B}(\mathbf{H}_s(\mathbf{C}, \mathbf{E})), \tag{3}$$

where τ_s is the expected cell life time in the absence of any metabolism[4]. The latter can also be interpreted as a measure of minimum maintenance requirements [34] that would be added as a constant negative

[3]More precisely, the maximum forward reaction rate for any reaction can be an *arbitrary* function of $\mathbf{C}$, $\mathbf{E}$ and time. Similarly, the maximum active uptake rate for a metabolite can be an arbitrary function of $\mathbf{C}$, $\mathbf{E}$ and time. In turn, $\mathbf{E}$ can be any arbitrary independent function of time or even a stochastic process. Similarly for the maximum active export rates and the maximum passive uptake/export rates. Any reaction rate H_{rs} is fixed to zero if the corresponding gene is absent in a cell.

[4]Cell death is modeled as a Poissonian process, i.e. inactive cells die at an exponential rate. The expected life time τ_s can be a positive constant or any arbitrary function of time, metabolite concentrations $\mathbf{C}$ and environmental variables $\mathbf{E}$. For example, τ_s might be a decreasing function of ethanol concentration. If one is primarily interested in the exponential

term to the objective function.

Metabolite uptake and export by cells directly affects environmental metabolite concentrations, which change as

$$\frac{d\mathbf{C}}{dt} = -\sum_{s=1}^{S} N_s \mathbf{F}_s(\mathbf{C},\mathbf{E}) + \mathbf{b}\sum_{s=1}^{S} \frac{\mu_s}{\tau_s} N_s + \mathbf{f}(t,\mathbf{C},\mathbf{E}). \tag{4}$$

The 1st sum in Eq. (4) iterates over all species and represents the net metabolite uptake by the entire microbial community. The 2nd sum in Eq. (4) accounts for nutrient recycling through cell death. The constant vector $\mathbf{b} \in \mathbb{R}^M$ quantifies released metabolites per dead dry biomass and can be adjusted to empirical biomass stoichiometry [36]. For example, cell death might be associated with an instantaneous release of $C_6H_{12}O_6$, NH_4^+ and PO_4^{3-} into the environment, at proportions given by the Redfield ratio [68, 23, 12]. The vector $\mathbf{f}$ in Eq. (4) accounts for possible external fluxes into and out of the system, for example due to sedimentation, precipitation or primary production. Environmental stochasticity can be incorporated by defining $\mathbf{f}$ as a stochastic process. Each of the terms in Eq. (4) can be omitted for individual metabolites. In fact, any metabolite concentration C_i can alternatively be specified as an independent function of time, a stochastic process or a function of $\mathbf{E}$ and other C_j $(j \neq i)$, independent of any microbial activity. For example, some metabolites can be defined through conjugate acid-base relationships (see section 5.2.2 for options regarding these so called *environmental metabolite dynamics*)

Finally, each environmental variable E_i can itself be an explicit function of time or a stochastic process, or depend on $\mathbf{C}$ and other E_j $(j \neq i)$. In fact, E_i can be dynamic, with a rate of change dE_i/dt specified as a function of time, $\mathbf{C}$ and $\mathbf{E}$.

Technical note: The model does not necessarily preserve total mass or chemical elements, unless it is carefully configured. In fact, MCM has no way of telling whether a chemical reaction makes any physical sense.

An overview of the model framework is given in Fig. 3. MCM allows the specification of arbitrary models of this structure, with any number of involved metabolites, reactions, cell species and environmental variables (see section 5). Simulations of the model are performed by solving the differential equations (3) and (4) together with the cell-metabolic optimization problems at each time step (see Fig. 4 for an illustration).

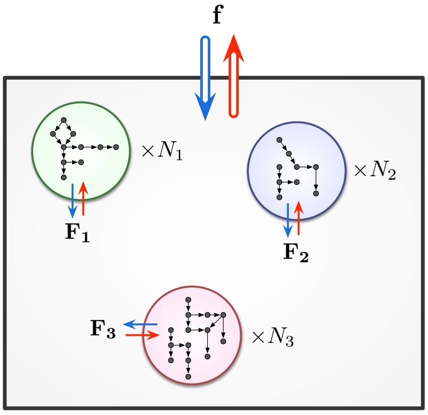

Figure 3: Illustration of the microbial community model framework described in section 1.3, here for 3 cell species. Thick arrows represent metabolite fluxes into and out of the ecosystem (e.g. due to fertilization in the case of a soil microbiome). Thin arrows represent per-cell metabolite fluxes across cell membranes. The graphs inside the cells represent the cell-metabolic models. Individual cell metabolism is predicted using flux balance analysis.

growth phase, τ_s can also be set to infinity (no cell death). It is also possible to include the death term, $-N_s/\tau_s$, only when the cell production rate, $\mathcal{B}/\mu_s$, falls below a certain threshold.

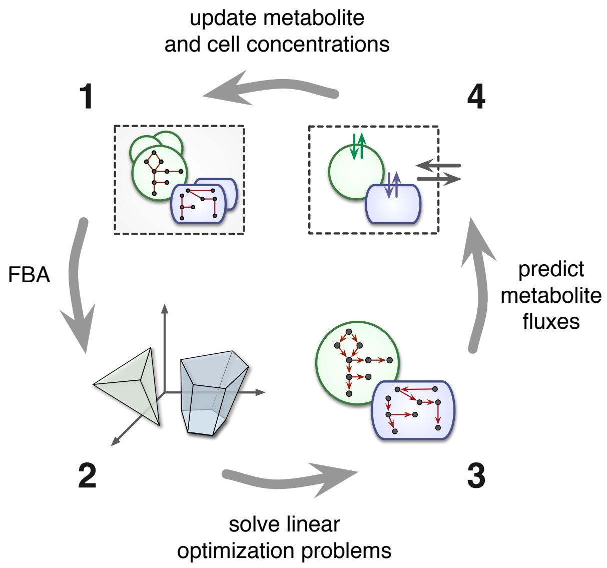

Figure 4: Illustration of the simulation of a microbial community model. Each iteration consists of four main steps: Flux Balance Analysis (FBA) is used to translate cell-metabolic potential, metabolite concentrations and environmental conditions (1) into a linear optimization problem (LOP) for the biomass production rate of each cell species (2). Each LOP optimizes a linear function constrained to a polytope in high-dimensional space. Optimizing biomass production rates yields predictions on intracellular reaction rates (3) and thus microbial metabolite uptake/export rates (4). Metabolite fluxes are used to predict metabolite concentrations and biomass production rates are used to predict cell population sizes in the next time step (1). Optional environmental variables are also updated.

2 Introductory example

In this section we shall construct and analyze a simple model of a two-species nitrifier community. Our model will only comprise 5 metabolites (ammonium `NH4`, nitrite `NO2`, nitrate `NO3`, oxygen `O2` and water `H2O`), 2 redox reactions (aerobic ammonium oxidation to nitrite, or `amo`, and aerobic nitrite oxidation to nitrate, or `nxr`) and 2 cell species (`Nitrosomonas` and `Nitrobacter`). `amo` and `nxr` will only be performed by `Nitrosomonas` and `Nitrobacter`, respectively, and both reactions will be identified with biomass synthesis. An overview of the metabolic network in terms of FBA is given in Fig. 2. This simplistic model should serve as an illustrative example of MCM's working principles. For an elaborate documentation of MCM please consult the remaining sections, in particular sections 5, 6, 7, 8 and 9. More realistic examples using published cell-metabolic models are given in sections 10, 11 and 12. For instructions on installing MCM consult section 3.

2.1 Constructing a microbial community model

MC models are defined using a human-readable formal syntax in a series of special MC model configuration files. In a new empty directory (let's call it `MC_intro_model`), create 4 empty plain text files and name them `metabolites`, `reactions`, `species` and `focals` (these file names must be used exactly). In the first 3 files we will be defining all relevant metabolites, reactions and cell species, respectively. The `focals` file will list the model objects (i.e. metabolites, reactions and species) that we wish to focus on in our analysis. Whether a metabolite, species or reaction is focal or not does only affect the amount of output produced by MCM but has no effect on the microbial community dynamics. While not particularly obvious in this simplistic example, this is very useful for more realistic large-scale metabolic models that can comprise several hundreds of metabolites and reactions [1, 44], only a few of which might be of real interest.

Let's start by defining all relevant metabolites: In the file `metabolites`, write:

```
file_version: 1.3 # do not remove, move or change this line

NH4
    description: ammonium
    max active uptake rate:  Monod 1.23e-13 2.6e-5 # Monod kinetics, requires Vmax and Khalf
    max active export rate:  unlimited
    max passive uptake rate: 0
    max passive export rate: 0
    environmental dynamics:  initial 1e-4 flux microbial_export

NO2
    description: nitrite
    max active uptake rate:  Monod 3.25e-13 2.29e-4
    max active export rate:  unlimited
    max passive uptake rate: 0
    max passive export rate: 0
    environmental dynamics:  initial 0 flux microbial_export

NO3
    description: nitrate
    max active uptake rate:  0
    max active export rate:  unlimited
    max passive uptake rate: 0
    max passive export rate: 0
    environmental dynamics:  initial 0 flux microbial_export

O2
    description: oxygen
    max active uptake rate:  1e-13
    max active export rate:  unlimited
    max passive uptake rate: 0
    max passive export rate: 0
    environmental dynamics:  concentration 0.217e-3   # 100% saturation with atmosphere

H2O
    description: water
    max active uptake rate:  unlimited
    max active export rate:  unlimited
    max passive uptake rate: unlimited
    max passive export rate: unlimited
    environmental dynamics:  concentration 0
```

click here for source code

Observe that each metabolite is defined by a unique name (see section 5.13.1 for name restrictions) and a list of mandatory key:value pairs (one per line). Comments are preceded by the "#" symbol and are ignored by MCM. Excessive whitespace is also ignored. The `description` of a metabolite can be empty or contain a short descriptive text to be included in MCM's latter output.

In general, metabolites can be transported between cells and environment either *actively* (e.g. using a transport membrane protein) or *passively* (e.g. via diffusion). In MCM, active transport differs from passive transport in that the availability of an active transport mechanism needs to be explicitly specified for a cell species (see below), while passive transport kinetics are applicable whenever an active transport mechanism is absent. In the example above, all `max passive uptake/export rate`s except for water are set to zero, so that exchange only takes place if a cell is explicitly specified to possess the appropriate active transport mechanism. Most `active uptake/export rates` are assumed to be `unlimited` (i.e. are not explicitly constrained), except for the `O2`, `NH4`, `NO2` and `NO3` uptake rates, whose limits are either set to a constant value or depend on substrate concentrations in a Monod-like manner. Other more complicated transport kinetics are also possible and are described in full detail in section 5.2.1.

The `environmental dynamics` of a metabolite specify how its concentration in the environmental pool changes with time. In the example above, the concentrations of `NH4`, `NO2` and `NO3` are dynamical and their

rate of change depends on net microbial export (which might be negative). Replacing microbial_export in their environmental dynamics with a numerical constant (or a function of time, t) would mean that their concentration changes at that constant (or time-dependent) rate. While the NH4 concentration is initially 10 μM, both NO2 and NO3 are initially absent from the environment. The O2 concentration is explicitly set to be constant and at 100% saturation with the atmosphere (at 37°C). Alternatively, the O2 concentration could have been an explicit function of time (t) or interpolated from available measurements. We arbitrarily set the concentration of water to constant 0, however water is only considered as a metabolic by-product and will not affect our model's dynamics. The full range of possible environmental metabolite dynamics is described in section 5.2.2.

Technical note: All physical quantities and constants must be given in specific (typically SI-derived) physical units. For example, time is given in days, half-saturation constants and metabolite concentrations are given in mol/L and metabolite export rates are given in mol/(cell · day). See section 5.13.4 for a full list of appropriate units.

Next, let's define the two redox reactions, ammonium oxidation (amo) and nitrite oxidation (nxr). In the reactions file, insert the following:

```
file_version: 1.3 # do not remove, move or change this line

amo
    description:      ammonia oxidation to nitrite
    equation:         NH4 + 1.5 O2 -> NO2 + H2O
    max forward rate: unlimited  # reaction can be performed arbitrarily fast
    max reverse rate: 0          # reaction is non-reversible
    objective:        4.6        # g dry biomass produced per mol reaction flux

nxr
    description:      nitrite oxidation to nitrate
    equation:         2 NO2 + O2 -> 2 NO3
    max forward rate: 1e-12      # reaction flux constrained to below 1e-12 mol/(cell*day)
    max reverse rate: 0
    objective:        3.7
```

click here for source code

Similarly to metabolites, each reaction is defined by a unique name and a set of mandatory key:value pairs (one per line). Each reaction is characterized by a chemical equation that describes the transformation of a set of reactants into a set of products. Only pre-defined metabolites can be used in chemical equations and names are case-sensitive (i.e. nh3 is not the same as NH3). Reactants and products are separated by "->" and can be preceded by an optional stoichiometric coefficient (note the space between the latter and the metabolite name). In the above example, both reactions are non-reversible. amo has an unlimited (i.e. unconstrained) max forward rate (in our model amo will, however, be limited by the finite NH4 uptake rate). On the other hand, nxr has a finite max forward rate due to (hypothetical) finite enzyme capacities. In general, reaction rate limits can be functions of metabolite concentrations (even of non-participating metabolites such as inhibitors) or other environmental variables (see section 5.3.3). The objective of a reaction (e.g. 4.6 for amo) specifies the amount of biomass produced per reaction flux (g dry weight per mol).

In the species file, define the two cell species, Nitrosomonas and Nitrobacter:

```
file_version: 1.3 # do not remove, move or change this line

Nitrosomonas
    initial concentration: 1e8          # initial cell concentration (1/L)
    mass:                  3e-13        # dry cell mass (g/cell)
    life time:             infinite     # ignore cell death
    reactions:             amo
    active uptakes:        O2, NH4      # cells can actively take up O2 and NH4
    active exports:        NO2

Nitrobacter
    initial concentration: 1e8
```

```
    mass:                    4e-13
    life time:               20          # inactive cells live on average 20 days
    reactions:               2 * nxr
    active uptakes:          O2, NO2
    active exports:          NO3
```

click here for source code

Similarly to metabolites and reactions, each cell species is defined by a unique name and a list of mandatory key:value pairs. The `initial concentration` specifies the cell concentration at time 0. The `life time` specifies the expected time until cell death in the absence of metabolic activity. Here, we have omitted to model cell death for `Nitrosomonas` while we assume that a metabolically inactive `Nitrobacter` cell lives on average 20 days. In general, a cell's `life time` may explicitly depend on time, metabolite (e.g. toxin) concentrations or environmental variables such as temperature (see section 5.4.1).

The `active uptakes` and `active exports` lists specify the metabolites (separated by a comma) for which active uptake and export mechanisms are available, respectively. The corresponding uptake/export rate limits for each actively transported metabolite are taken as specified in the `metabolites` file (see above). Since we specified the `max passive uptake/export rate` of `H2O` as `unlimited` (see above), both species can also freely exchange water with their environment.

In the above example, each species can only perform one of two redox reactions, `amo` or `nxr`. If a cell can perform several reactions, these need to be separated by a comma. Note that `Nitrobacter` is specified to have two copies of the `nxr` gene. While this has no effect on the cell's metabolic activity (which is determined according to FBA), specifying gene copy numbers (by default assumed to be one) is useful when comparing simulations of gene concentrations to quantitative metagenomic time series [16].

Finally, let us specify focal (i.e. particularly interesting) metabolites, reactions and species. In the `focals` file, insert the following:

```
file_version: 1.3 # do not remove, move or change this line

FOCAL_METABOLITES
  NH4
  NO2
  NO3

FOCAL_REACTIONS
  amo
  nxr

FOCAL_SPECIES
  *   # all species are focal
```

click here for source code

In this example we do not include `O2` nor `H2O` in the `focals` list because their dynamics are trivial anyway. On the other hand, we included all cell species using the special wildcard "*".

2.2 Running a single simulation

Now that we've defined our MC model, let us prepare an MCM script that will contain all necessary commands to run our simulation. In the parent directory containing our `MC_intro_model` directory, create a plain text file (let's call it `script_intro`) containing the following:

```
# specify the MC model directory
set MCmodelDir "MC_intro_model"

# create a new non-existing output directory
# all simulation results will be saved in here
setodnew "simulations_intro/run_"
```

```
saveScript    # save a backup copy of this script to the current output directory
saveMCmodel   # save a backup copy of the MC model to the current output directory

# adjust simulation parameters
set integrationTimeStep 0.01  # integration time step in days
set maxTimeSeriesSize   100   # record about 100 time points
set maxSimulationTime   15    # simulate 15 days

# plot metabolic activity heatmaps every 5 days
set plotMetabolicActivityHeatmaps start 0 step 5

# enable parallel computation
set parallel

# run simulation
runMCM

# open output directory and quit MCM when done
openod
quit
```

click here for source code

Notice that most MCM commands fall into one of three categories:

- `set <control variable name> <some value>`, for setting the value of a control variable. For example, "`set maxSimulationTime 15`" sets the time span of simulations to 15 days.
- `set` (or `unset`) `<flag name>`, for toggling a boolean flag on or off. For example, "`set parallel`" enables parallel computation, i.e. using multiple CPUs or cores to solve FBA models in parallel.
- `<some standalone command>`, for invoking a simulation or other comparable tasks.

Notable exceptions exist, such as `setodnew` (written as a single word and followed by a quoted file path), which creates a new non-existing output directory with the given path (a number is appended to the directory name to ensure non-existence).

Technical note: All unspecified control variables and flags have default values, which can be retrieved using the `state` command.

Similarly to MC model configuration files, MCM ignores comments in scripts that are preceded by the "#" symbol, as well as excessive whitespace. Commands are case-sensitive, e.g. `quit` is valid but `Quit` is not. See sections 4.1 and 4.4 for more information on MCM's command line interface and MCM scripts. For an overview of commands and options you can also run MCM in the terminal and type `help` in the MCM command line.

The command actually invoking the simulation in the example above is `runMCM`; the remaining commands simply modify control variables or prepare the output directory into which results are to be saved. The above script can be executed in the terminal by calling MCM with the script file path as an argument, e.g.

```
./MCM script_intro
```

Upon completion, you should obtain a new directory `simulations_intro/run_01`, into which all simulation results are saved. MCM saves all results in the form of strictly structured data and plot files, though some overview output is reproduced in the command line. Within the specified output directory, all output files have a fixed name and location. However, which files are created depends both on the invoked MCM function as well as current control variable and flag values. In our case the sub-output directory `simulation_summary` will contain the predicted temporal profiles of several community-wide summary variables, such as metabolite concentrations, reaction rates or cell concentrations (see Fig. 5 for an example). The sub-output directory `metabolic_activity_heatmaps` will contain metabolic activity heatmaps generated at regular time intervals (see Fig. 6 for an example). See section 13 for a list of possible output files and section 6 for detailed simulation options.

Note: At this point you should make a backup of the model and script, as we will be reverting to it in subsequent examples.

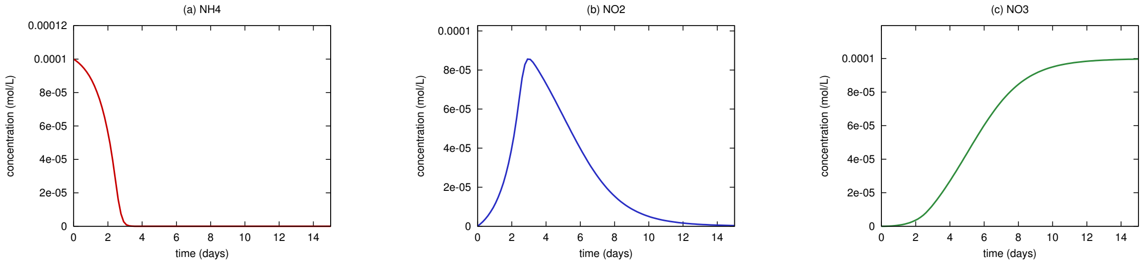

Figure 5: Predicted `NH4`, `NO2` and `NO3` concentrations for the simple nitrifier community model (section 2.2). The initially present `NH4` is quickly oxidized by `Nitrosomonas` to `NO2`, which is in turn oxidized by `Nitrobacter` to `NO3`, albeit with a certain time lag due to initially low `Nitrobacter` populations.

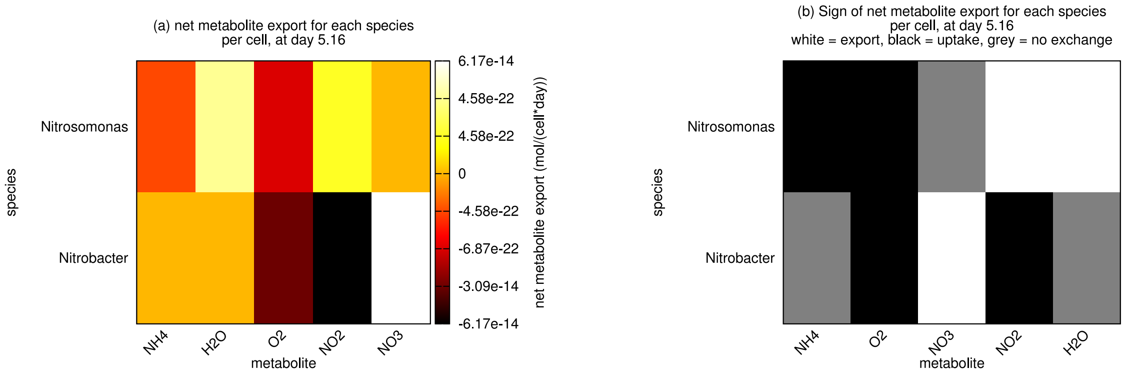

Figure 6: (a) Predicted metabolic activity (net metabolite export per cell) at day 5 for the simple nitrifier community model (section 2.2). (b) Sign of net metabolite fluxes, emphasizing net export (white) and uptake (black). Columns including both a black and white entry (`NO2` in this case) indicate metabolite exchange between species.

2.3 Uncertainty analysis

As simple as the example above is, it already requires the specification of a respectable number of numeric parameters (5 uptake kinetic parameters, 2 initial metabolite concentrations, 2 cell masses, 2 initial cell concentrations and 1 cell life expectancy). In practice, several parameters might be poorly quantified or even random. If the probability distribution of parameters is known (e.g. as a Bayesian posterior), MCM can project the *uncertainty* of input parameters to an uncertainty of the microbial community's behavior. MCM's *uncertainty analysis* involves several Monte Carlo simulations of the MC model and aims to estimate the probability distribution of model predictions corresponding to the pre-defined probability distribution of model parameters. Uncertainty analysis is described in detail in section 9.

Model parameters included in uncertainty analysis need to be defined as *symbolic*. In short, this means assigning them a name, a value range, a default value and a probability distribution. In general, symbolic parameters allow a higher level analysis of microbial communities, including sensitivity analysis, uncertainty analysis and parameter estimation from data. See section 5.7 for more details on symbolic parameters.

Let us define two symbolic parameters, `Khalf_NH4` and `initial_Nb`, representing the `NH4` uptake half-saturation constant and initial `Nitrobacter` cell concentration, respectively. Create a plain text file **`parameters`**, place it in the MC model directory used in the previous example (`MC_intro_model`), and fill in the following content:

```
file_version: 1.3 # do not remove, move or change this line

Khalf_NH4
    description:  ammonium uptake half-saturation constant
    default:      2.6e-5
    minimum:      1e-5
    maximum:      1e-3
    distribution: uniform
    fixed:        no

initial_Nb
    description:  initial Nitrobacter cell concentration
    default:      1e8
    minimum:      1e7
    maximum:      1e9
    distribution: logUniform
    fixed:        no
```

click here for source code

Notice the familiar syntax: Each symbolic parameters is defined by a unique name and a list of key:value pairs (one per line). The first parameter is uniformly distributed within its permissible range (as defined by its `minimum` and `maximum`), while the 2nd parameter is log-uniformly distributed. For purposes other than uncertainty analysis or fitting, the `distribution` of a parameter can also be omitted. We defined both parameters to be non-`fixed`, because we will be varying them in the following analysis (`fixed` symbolic parameters always evaluate to their `default` value). We set their `default` value to the numeric values used in our original model in section 2.2.

So far MCM has no way of knowing what these parameters actually mean. To include them in our MC model we refer to them by their name, enclosed between two "$" symbols: In the `metabolites` configuration file (located in your MC model directory, `MC_intro_model`), change the active uptake kinetics of `NH4` to the following:

```
max active uptake rate:  Monod 1.23e-13 $Khalf_NH4$
```

Similarly, in the `species` file change the `Nitrobacter` initial cell concentration to

```
initial concentration: $initial_Nb$
```

In principle, any numerical value present in any of the MC model configuration files `metabolites`, `reactions` and `species`, can be replaced by a symbolic parameter. In the script file `script_intro` from the previous section replace the `runMCM` command with the following:

```
# reduce verbosity (i.e. amount of information printed out during computation)
# for a minimal progress report set this to 0
set UA_verbosity 2

# specify number of random simulations for uncertainty analysis
set UA_MonteCarloTrials 100

# randomly initialize random number generation
seed

# perform uncertainty analysis
UAMCM
```

click here for source code

Observe that we randomly initialize MCM's random number generator using the command `seed`. This is to ensure independent results each time we perform an uncertainty analysis. Execute the modified script as previously, e.g. by calling

```
./MCM script_intro
```

in your terminal. Through a series of Monte Carlo simulations, MCM will estimate the probability distributions of focal model variables. The estimated distributions are saved as percentile contour plots (see Fig. 7) along with detailed reports into the output directory.

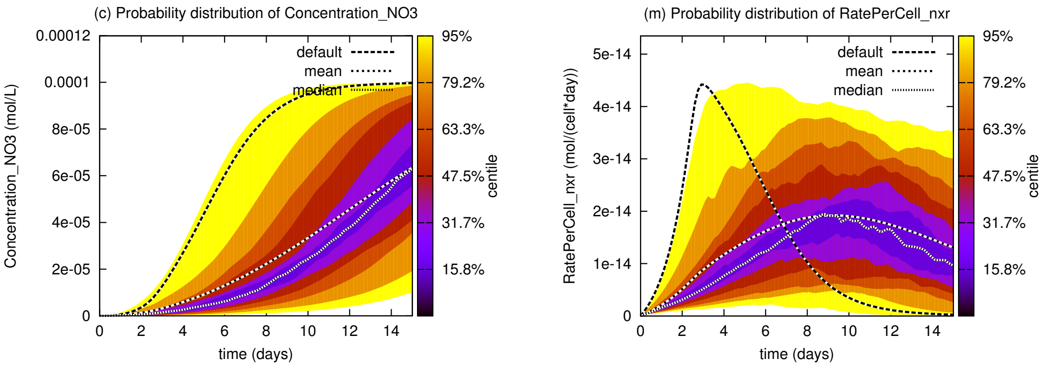

Figure 7: Probability distribution of (c) NO3 concentration and (m) nxr reaction rate per cell through time, as predicted by uncertainty analysis of the nitrifier community model (section 2.3). Each color region represents a percentile centered around the median. Also shown are the mean as well as the prediction based on default parameter values. Excerpt from the output file UA.pdf.

2.4 Sensitivity analysis

An alternative to uncertainty analysis, sensitivity analysis (SA) aims to quantify the dependence of a system's behavior on individual parameters, either when these are only slightly varied (*local* SA), or when these vary between extreme values (*global* SA) [7]. In MCM, local and global sensitivity analysis are invoked using the commands LSAMCM and GSAMCM, respectively.

Similarly to uncertainty analysis, SA requires the specification of non-fixed symbolic parameters, the sensitivity to which is to be evaluated. Contrary to uncertainty analysis, SA does not require (nor does it use) the probability distributions of parameters. Instead, local SA evaluates symbolic parameters close to their default value in order to estimate the partial derivatives of output variables with respect to these parameters, while global SA evaluates symbolic parameters at their default, minimum and maximum values (as defined in the parameters file). SA is described in detail in section 8.1.

For this example, we can just leave our MC model configuration, including the parameters file, unchanged from the previous section. In the script file script_intro, replace all uncertainty analysis-associated commands (the ones we added in the previous section) with the following commands:

```
# reduce verbosity (i.e. amount of information printed out during computation)
set SA_verbosity 2

# enable hierarchical clustering of heatmaps
set SA_clusterParameters
set SA_clusterVariables

# choose which observables to include
set SA_includeMetaboliteConcentrations
set SA_includeMetaboliteExportCommunityWide
set SA_includeCellConcentrations
unset SA_includeMetaboliteExportPerCell
unset SA_includeReactionRatesCommunityWide
unset SA_includeReactionRatesPerCell
unset SA_includeCellConcentrationsDeadAlive
unset SA_includeCellGrowthRates
```

```
unset SA_includeGeneConcentrations
unset SA_includeGeneConcentrationsDeadAlive

# create a sub-output directory and run LSA
changeod "LSA"
LSAMCM

# create another sub-output directory and run GSA
changeod "../GSA"
GSAMCM
```

click here for source code

Executing the new script will result in two sub-output directories, LSA and GSA, containing detailed reports and graphical summaries of the local and global sensitivity analysis, respectively. Fig. 8 exemplifies some of the generated output.

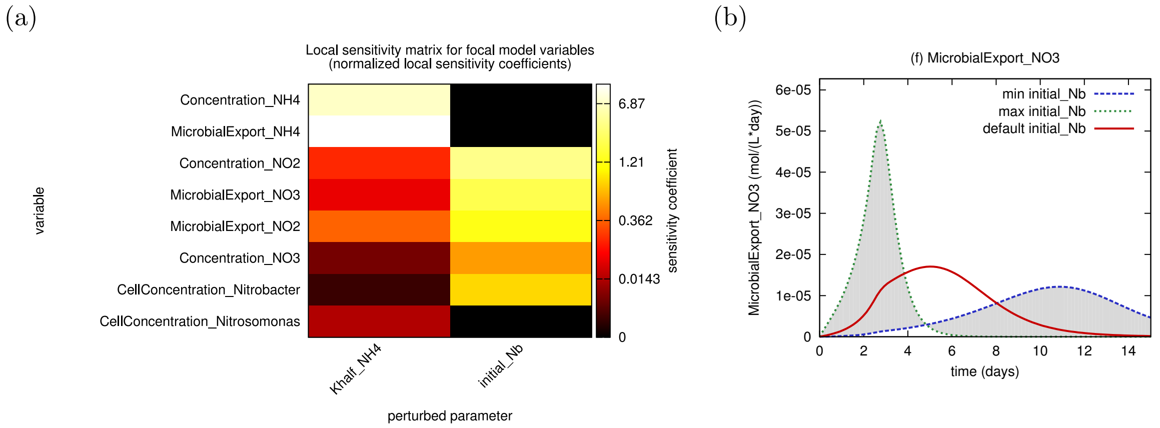

Figure 8: (a) Local sensitivity heatmap for the nitrifier community model (section 2.4). Each entry quantifies the absolute relative response of the variable in that row to small variations of the parameter in that column, integrated over time (see section 8.1 for a mathematical definition). Observe that the initial Nitrobacter concentration does not seem to influence the dynamics of NH4 nor Nitrosomonas (as expected). (b) Variation of microbial NO3 export when the initial_Nb parameter (initial Nitrobacter cell concentration) takes on its minimum or maximum values, calculated via global sensitivity analysis of the nitrifier model. Excerpts from the files LSA_heatmap.pdf and GSA_responses_to_initial_Nb.pdf.

2.5 Comparing and fitting the model to data

As valuable as it is to make predictions based on mechanistic models, the loop of scientific enquiry is only closed when predictions are validated against, and models are improved in view of, real measurements. MCM allows the statistical evaluation of model predictions against time series data (such as optical cell concentrations, rate measurements or metagenomics) as well as the calibration (*fitting*) of model parameters to the data using maximum-likelihood estimation. For more details on the underlying statistical theory as well as the fitting process consult section 7.

Simulation results are automatically compared to any time series data available. The latter needs to be given in tabular format in plain text files with a particular name structure. Consult section 14.3.6 for details on data file formats. In this example, we will be using an artificial time series data set for (a) Nitrobacter cell concentrations and (b) NO3 concentration. The former will be in optical density units, i.e. not calibrated to actual cell counts. As a result, MCM will estimate the appropriate linear unit scaling that best matches the simulation to the data. Create a new directory (let's call it data_intro) and two subdirectories, CellConcentrationsDeadAlive and MetaboliteConcentrations. The names of these subdirectories are used by MCM for identification purposes and must be as given here. Create two plain text files, "Nitrobacter [au]" and "NO3", and place them in the first and second data subdirectory that you created, respectively. These files will contain our two time series. We designate the first time series

as *dead+alive* cell concentrations because suspended dead cells also contribute to light scattering during optical measurement and can thus not be differentiated from living cells. The " [au]" suffix in the file name tells MCM that the data is in arbitrary (unknown) units.

In the "Nitrobacter [au]" time series file, fill in an artificial time series similar to the following:

```
# time series for Nitrobacter cell concentrations (dead+alive, artificial)
# uncalibrated units (optical concentration - negative control OD)
# time(day)    OD
0              0.2
2.1            0.23
4.0            0.22
8              0.61
8              0.54
10             0.89
13             0.98
13             0.71
14             0.92
```

click here for source code

Similarly, fill the NO3 time series file with something like the following:

```
# Time series for NO3 concentrations (artificial)
# time(days)    NO3_concentration(mol/L)
0               2.6e-6
1.2             5.1e-6
3               1.0e-5
5.1             2.2e-5
8               5.3e-5
8.2             4.9e-5
10              8.7e-5
12              9.4e-5
13              9.5e-5
13              9.8e-5
```

click here for source code

Observe that multiple values (e.g. from repeated measurements) can be given for the same time points. These will be averaged if the flag averageAmbiguousData is set or treated as independent time points otherwise. As in all MCM files, comments preceded by the "#" sign as well as excessive whitespace are ignored.

Having prepared our data sets, we now need to point MCM to the root data directory (which we called data_intro). In the original MCM script from section 2.2 we add the specification of our data directory, ending up with the following script:

```
# specify the MC model directory
set MCmodelDir "MC_intro_model"

# create a new non-existing output directory
# all simulation results will be saved in here
setodnew "simulations_intro/run_"

saveScript    # save a backup copy of this script to the current output directory
saveMCmodel   # save a backup copy of the MC model to the current output directory

# adjust simulation parameters
set integrationTimeStep 0.01  # integration time step in days
set maxTimeSeriesSize   100   # record about 100 time points
set maxSimulationTime   15    # simulate 15 days

# plot metabolic activity heatmaps every 5 days
set plotMetabolicActivityHeatmaps start 0 step 5
```

```
# enable parallel computation
set parallel

# point MCM to our data
set MCdataDir "data_intro"

# specify error distribution for the following variables
set errorModel_CellConcentrationsDeadAlive   logNormal
set errorModel_MetaboliteConcentrations      normal

# run simulation
runMCM

# open output directory and quit MCM when done
openod
quit
```

click here for source code

Observe that we also specify a log-normal error distribution for cell concentrations and a normal error distribution for metabolite concentrations. Simply put, this means that deviations of cell concentrations and metabolite concentrations from data are statistically evaluated on a logarithmic and linear scale, respectively. For a mathematical explanation of log-normal and normal error structures see section 7.

Executing the above script will run a single simulation similar to the one in section 2.2, using the default values of our symbolic parameters. However, now MCM finishes the simulation with a comparison of its predictions to the provided data. All related reports and plots (exemplified in Fig. 9) are saved to the sub-output directory `comparison_to_data`. For example, the file `comparison_to_data/comparison.pdf` shows an overview of the data comparison, including coefficients of determination, log-likelihoods and estimates of noise variance.

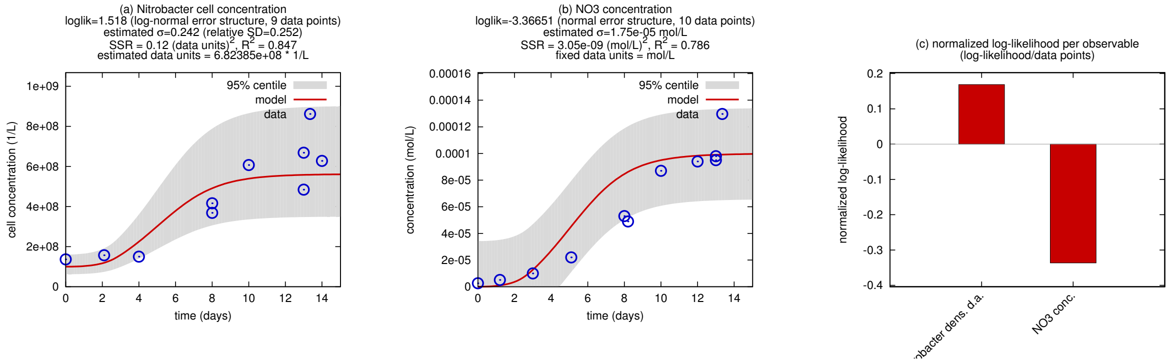

Figure 9: Summary plots of the comparison of the nitrifier community model (section 2.5) to artificial time series data for (a) `Nitrobacter` cell concentrations (dead+alive) and (b) `NO3` concentrations. The shaded region represents the estimated 95% error centile around the model predictions (under the assumption of the latter being the *true* deterministic component). Note that the units of the first data set are estimated at 6.8×10^8 cells/L per optical density unit. Fig. (c) summarizes the normalized log-likelihoods of the evaluated output variables. Excerpt from the file `comparison.pdf`.

Having compared our model to the available data (and most likely having obtained a moderate to bad match), let's go a step further and adjust the two symbolic parameters, `Khalf_NH4` and `initial_Nb`, to better match our data. A sensitivity analysis of the individual log-likelihoods (similar to the sensitivity analysis demonstrated in section 2.4 above), would help us infer the direction to which parameters should be adjusted in order to improve the match (see section 8.2 for details). We shall skip this intermediate step and directly attempt an automated maximum-likelihood estimation using MCM's fitting function.

Since we have already (i) specified an MC model, (ii) defined a set of non-`fixed` symbolic parameters and their permitted ranges (see section 2.3) and (iii) provided MCM with appropriate data, we are ready to proceed with the fitting. Modify the last script by replacing `runMCM` with the command `fitMCM`

and execute the script in the terminal as usual. The invoked parameter fitting is an iterative process and involves several repeated simulations with slightly varying parameter values, starting at their default values. A final simulation with the fitted parameters (i.e. maximizing the log-likelihood) and a comparison to the data are saved to the sub-output directory `run_final` (see Fig. 10). Following fitting, MCM will also estimate confidence intervals for the fitted parameters (this involves several more simulations). The final fit report (saved in the file `fit_report.txt`) is exemplified below:

```
Fitting completed after 178 simulations: max objective reached within tolerance
  Maximum normalized log-likelihood (MNLL) = 0.943069 (= log-likelihood/19 data points)
  Fitted parameter values:
    Khalf_NH4 = 0.000238477 (ammonium uptake half-saturation constant)
    initial_Nb = 1.27811e+08 (initial Nitrobacter cell concentration)

  95% confidence intervals for fitted parameters
  estimated from inverse observed Fisher information:
    Khalf_NH4 = 0.000238477 +/- 3.16e-05 (standard error = 1.612e-05 = 6.7 %)
    initial_Nb = 1.27811e+08 +/- 3.069e+07 (standard error = 1.566e+07 = 12.3 %)

Finished after 222 simulations
```

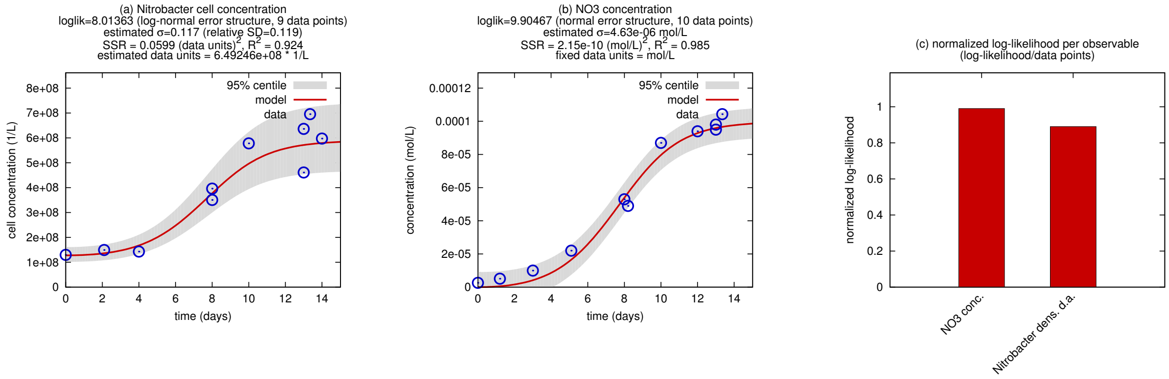

Figure 10: Comparison of the nitrifier community model to time series data for (a) `Nitrobacter` cell concentrations (dead+alive) and (b) `NO3` concentrations, following a maximum-likelihood fit of the parameters `Khalf_NH4` and `initial_Nb` (section 2.5). Note that since the `Nitrobacter` cell concentration data was provided in unknown units, a calibration to actual cell counts was also performed (one optical density unit corresponding to 6.46×10^8 cells/L). Fig. (c) summarizes the normalized log-likelihoods of the evaluated variables. The overall log-likelihood after fitting is 17.9, compared to -1.84 prior to fitting (see Fig. 9). Excerpt from the file `run_final/comparison.pdf`.

Note that the example model considered here is very simple and *well-behaved*. For realistic large-scale models parameter fitting might (a) take much longer to converge, (b) fail for unexpected reasons or (c) fail to estimate parameter confidence intervals. This is particularly likely when many parameters are fitted simultaneously or when the model responds highly non-linearly to parameter changes. These issues are characteristic of the (dreaded) *inverse problem*, i.e. the calibration of mechanistic models to experimental data [90]. Resolving them typically requires a good understanding of the model and extensive tweaking of the fitting process. For a detailed explanation of MCM's fitting options consult section 7.3.

2.6 Fitting the model using arbitrary composite data

In the previous example we calibrated our model using available data for two variables: `Nitrobacter` cell concentrations and `NO3` concentrations. In practice some variables may not be observed directly, but only inferred indirectly from other composite or *observable* variables. For example, the cell concentrations of the nitrifiers *Nitrosomonas* and *Nitrobacter* might not be separately measurable, but the total concentration of nitrifiers might be. In principle, such composite data can still provide valuable information on an ecosystem's state.

In the following example we shall define a new observable, nitrifier_concentration, as the total concentration of Nitrosomonas and Nitrobacter. Create a new text file observables, place it into the existing MC model directory (MC_intro_model), and fill in the following:

```
file_version: 1.3 # do not remove, move or change this line

nitrifier_concentration
  value:       Nitrobacter + Nitrosomonas
  error model: normal
```

In general, observables can be functions of any other variables such as metabolite concentrations, reaction rates or even other observables (section 5.5). While observables do not themselves influence the behavior of a model, they can be used for uncertainty analysis, sensitivity analysis and model fitting just like any other variable (e.g. as in the examples above, sections 2.3, 2.4 and 2.5).

Observe that we specified the error model of nitrifier_concentration as normal, which means that this particular observable shall be compared to data assuming a normal error distribution. Let us compare and then calibrate our model using a (hypothetical) time series for nitrifier concentrations. Similarly to the example in section 2.5, create a text file nitrifier_concentration, place it into a new directory data_intro/Observables/, and fill in a time series similar to the following:

```
# time series for nitrifier cell concentrations (artificial)
# time(day)    cells/L
0.5            3e8
2.3            7.2e8
4.0            2.1e9
8              2.3e9
10             2.2e9
13             2.1e9
14             2.05e9
```

click here for source code

In the previous MC script (section 2.5) set the error models of cell and metabolite concentrations to none, so that MCM only uses the nitrifier_concentration data for comparison. We thus end up with the following script:

```
# specify the MC model directory
set MCmodelDir "MC_intro_model"

# create a new non-existing output directory
# all simulation results will be saved in here
setodnew "simulations_intro/run_"

saveScript    # save a backup copy of this script to the current output directory
saveMCmodel   # save a backup copy of the MC model to the current output directory

# adjust simulation parameters
set integrationTimeStep 0.01  # integration time step in days
set maxTimeSeriesSize   100   # record about 100 time points
set maxSimulationTime   15    # simulate 15 days

# plot metabolic activity heatmaps every 5 days
set plotMetabolicActivityHeatmaps start 0 step 5

# enable parallel computation
set parallel

# point MCM to our data
set MCdataDir "data_intro"

# specify error distribution for observables
set errorModel_CellConcentrationsDeadAlive   none
```

```
set errorModel_MetaboliteConcentrations      none

# run simulation with default parameters, saving results in a sub-output directory
changeod "run_default"
runMCM

# fit model and save results in a separate sub-output directory
changeod "../fitting"
fitMCM

# open output directory and quit MCM when done
openinod ".."
quit
```

click here for source code

The above script first simulates the default model and then invokes MCM's fitting routine, which completes after about 100 simulations. Fig. 11 compares the fitted model to the data used for calibration. As this example demonstrates, in principle a single time series of any arbitrary observable (here, `nitrifier_concentration`) can be used to calibrate multiple model parameters (here, `Khalf_NH4` and `initial_Nb`), as long as the calibrated parameters somehow influence the model predictions being compared to the data.

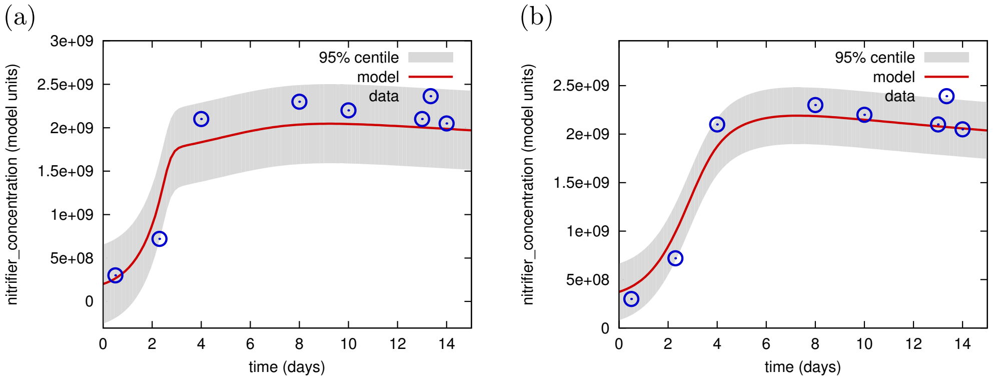

Figure 11: Comparison of the nitrifier community model to time series for `nitrifier_concentration`, (a) with default parameter values and (b) following a maximum-likelihood parameter calibration (section 2.6). Excerpt from the file `fitting/run_final/comparison.pdf`.

2.7 Including environmental stochasticity

Natural microbiomes are inevitably subject to fluctuations in environmental conditions, such as precipitation, temperature or nutrient influx. In the following example, we shall extend our nitrifier model to include two sources of environmental stochasticity: (a) fluctuations in `NH4` supply rates and (b) fluctuations in temperature, which in turn influence reaction rates. We shall then perform an uncertainty analysis of the resulting (stochastic) model, similar to the one in section 2.3.

To eliminate any stochasticity due to parameter uncertainty, we set both symbolic parameters (`Khalf_NH4` and `initial_Nb`) to `fixed`. Hence, the `parameters` file should now look as follows:

```
file_version: 1.3 # do not remove, move or change this line

Khalf_NH4
    description:  ammonium uptake half-saturation constant
    default:      2.6e-5
    minimum:      1e-5
    maximum:      1e-3
    distribution: uniform
```

```
    fixed:        yes

initial_Nb
    description:  initial Nitrobacter cell concentration
    default:      1e8
    minimum:      1e7
    maximum:      1e9
    distribution: logUniform
    fixed:        yes
```

click here for source code

As a result, `Khalf_NH4` and `initial_Nb` will always evaluate to their default values. We then modify the `environmental dynamics` of `NH4` to include a random external supply rate: In the `metabolites` file, modify the `NH4` record to the following:

```
NH4
    description: ammonium
    max active uptake rate:  Monod 1.23e-13 $Khalf_NH4$
    max active export rate:  unlimited
    max passive uptake rate: 0
    max passive export rate: 0
    environmental dynamics:  initial 1e-4 flux logOU 1e-4 1e-5 5 and microbial_export
```

click here for source code

`NH4` is now replenished at a rate which is itself a stochastic log-Ornstein-Uhlenbeck process[5] with a mean of 1×10^{-4} mol/(L · day), a standard deviation of 1×10^{-5} mol/(L · day) and a correlation time of 5 days.

To define temperature as an environmental variable, create a new plain text file called `environment`, place it in the MC model directory (which we called `MC_intro_model`) and fill in the following:

```
file_version: 1.3 # do not remove, move or change this line

temperature
   dynamics: value 293 and OU 5 10
   units:    K
   sign:     positive
```

click here for source code

Notice the familiar syntax: Each environmental variable is defined by a unique name and a set of key:value pairs (one per line). The option `units` (optional) merely specifies axis labels during graphical output, and `sign:positive` (optional) ensures that `temperature` is always positive regardless of numerical errors or flaws in the model. The most important aspect of an environmental variable is its `dynamics`, which either specifies its `rate` of change, or its explicit `value` as is the case above. `value` can be, for example, a function of time (`t`), metabolite concentrations and other environmental variables, or an interpolation of a measured time series, or even a stochastic process. Consult section 5.1 for more details on environmental variables. In our case, `temperature` fluctuates around 293 K as an Ornstein-Uhlenbeck stochastic process with a standard deviation of 5 K and a correlation time of 10 days. For completion, let's tell MCM that we find temperature interesting: At the end of the `focals` file, add the following two lines:

```
FOCAL_ENVIRONMENT
   temperature
```

So far temperature has no effect on our model's dynamics. Let's make the maximum `amo` reaction rate scale exponentially with temperature by changing the `amo` definition in the `reactions` file to the following:

```
amo
    description:      ammonia oxidation to nitrite
    equation:         NH4 + 1.5 O2 -> NO2 + H2O
    max forward rate: custom 1e-14 * exp((temperature - 293)/5)
    max reverse rate: 0          # reaction is non-reversible
```

[5] A positive stochastic process whose logarithm is an Ornstein-Uhlenbeck process [91, 27].

```
    objective:         4.6        # g dry biomass produced per mol reaction flux
```

click here for source code

Observe that the max forward rate of amo is now a custom function of temperature (T). The custom keyword can generally be followed by *any* mathematical expression that depends on time (t), metabolite concentrations or environmental variables (see section 5.3.3 for more details).

Summarizing, we have so far (a) set all symbolic parameters to fixed, (b) added a stochastic influx of NH4 to the system, (c) defined temperature as a stochastic environmental variable and (d) included temperature effects on the amo reaction rate limits. Let us now modify the MCM script to perform an uncertainty analysis of the model in face of the introduced stochasticity. We can use the same script as in section 2.3, with the difference that we choose to run simulations a bit longer and use more Monte Carlo trials for higher accuracy. Hence, our new MCM script should look as follows:

```
# specify the MC model directory
set MCmodelDir "MC_intro_model"

# create a new non-existing output directory
# all simulation results will be saved in here
setodnew "simulations_intro/run_"

saveScript    # save a backup copy of this script
saveMCmodel   # save a backup copy of the MC model

# adjust simulation parameters
set integrationTimeStep 0.01  # integration time step in days
set maxTimeSeriesSize   100   # record about 100 time points
set maxSimulationTime   50    # simulate 50 days

# plot metabolic activity heatmaps every 5 days
set plotMetabolicActivityHeatmaps start 0 step 5

# enable parallel computation
set parallel

# reduce verbosity
set UA_verbosity 2

# randomly initialize random number generator
seed

# use more Monte Carlo trials
set UA_MonteCarloTrials 500

# perform uncertainty analysis
UAMCM

# open output directory and quit MCM when done
openod
quit
```

click here for source code

Executing the above script will perform an uncertainty analysis similar to section 2.3, the results of which are exemplified in Fig. 13. Typical temperature profiles and the resulting maximum amo reaction rates are shown in Fig. 12.

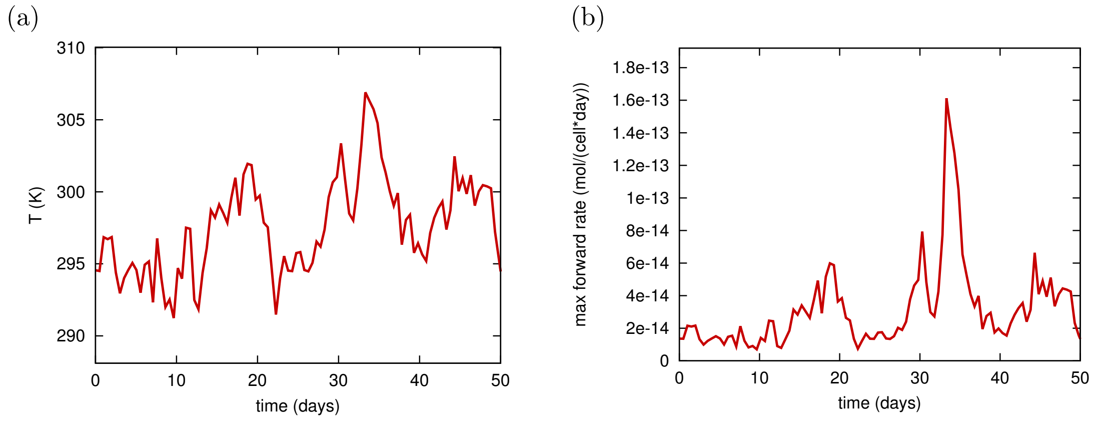

Figure 12: (a) Random temperature fluctuations and (b) resulting maximum amo reaction rates during a random simulation of the stochastic nitrifier community model (section 2.7).

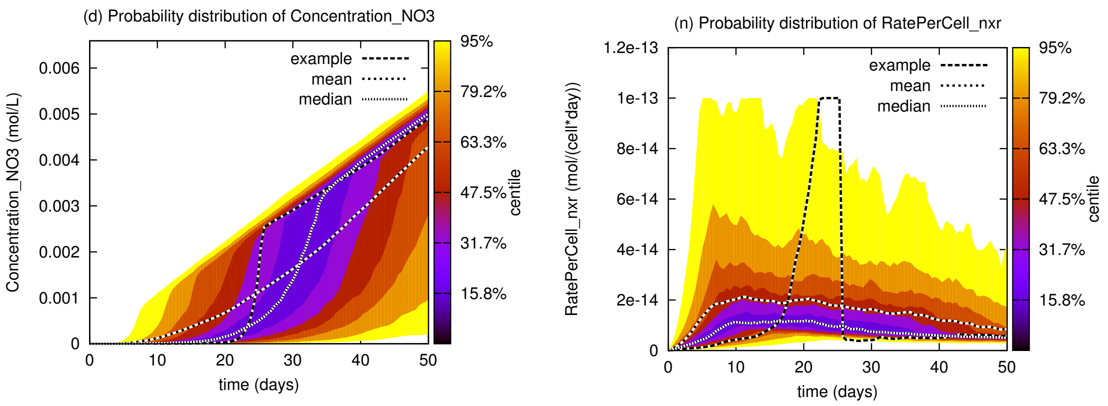

Figure 13: Probability distribution of (c) NO3 concentration and (e) nxr reaction rate per cell through time, as predicted by uncertainty analysis of the nitrifier model in the presence of environmental stochasticity (section 2.7). Also shown are the mean and a random example simulation. Excerpt from the output file `UA.pdf`.

2.8 Including bacteriophages

While long overseen, bacteriophages are increasingly perceived as important regulators of microbial communities, for example in the ocean [88] or bioreactors [84]. As we shall demonstrate here, MCM can accommodate bacteriophages in models, for example as dynamical environmental variables. Our starting point is the first deterministic model from section 2.1, hence we only keep the model files `metabolites`, `reactions`, `species` and `focals` while restoring them to their first version. Let us create an `environment` file defining two bacteriophages, specializing on Nitrosomonas and Nitrobacter, respectively:

```
file_version: 1.3 # do not remove, move or change this line

phage_Ns
    description: bacteriophage (Nitrosomonas-specialist)
    dynamics:    initial 1 rate 20*Nitrosomonas_infected - phage_Ns/10
    units:       viral particles/L
    sign:        positive


phage_Nb
    description: bacteriophage (Nitrobacter-specialist)
    dynamics:    initial 1 rate 20*Nitrobacter_infected - phage_Nb/10
```

```
    units:       viral particles/L
    sign:        positive
```

click here for source code

Observe that free viral particles are released at a rate of 20 particles per infected host cell per day, are lost at a rate of 0.1/day and have an initial concentration of 1/L. We specified the `sign` of the phage concentration as `positive` to ensure that these never become negative (e.g. due to numerical errors). Defining "infected" versions of the two cell species is straightforward: In the `species` configuration file add the two additional species `Nitrosomonas_infected` and `Nitrobacter_infected` and modify the existing `Nitrosomonas` and `Nitrobacter` to account for phage infections, obtaining the following:

```
file_version: 1.3 # do not remove, move or change this line

Nitrosomonas
    initial concentration:  1e8
    mass:                   3e-13
    life time:              infinite
    reactions:              amo
    active uptakes:         O2, NH4
    active exports:         NO2
    population growth:      birth and death and -1e-9*phage_Ns*Nitrosomonas


Nitrosomonas_infected
    initial concentration:  0           # no infected cells at the beginning
    mass:                   3e-13
    life time:              2           # reduced life expectancy of 2 days
    reactions:              amo
    active uptakes:         O2, NH4
    active exports:         NO2
    metabolic modulation:   0.5
    population growth:      birth and death and 1e-9*phage_Ns*Nitrosomonas


Nitrobacter
    initial concentration:  1e8
    mass:                   4e-13
    life time:              20
    reactions:              2 * nxr
    active uptakes:         O2, NO2
    active exports:         NO3
    population growth:      birth and death and -1e-9*phage_Nb*Nitrobacter


Nitrobacter_infected
    initial concentration:  0           # no infected cells at the beginning
    mass:                   4e-13
    life time:              2           # reduced life expectancy of 2 days
    reactions:              2 * nxr
    active uptakes:         O2, NO2
    active exports:         NO3
    population growth:      birth and death and 1e-9*phage_Nb*Nitrobacter
```

click here for source code

Observe that infected cells have a decreased life expectancy, when compared to their uninfected counterparts. Furthermore, cells become infected at a rate proportional to phage as well as uninfected cell concentration, i.e. according to mass action law. The dynamics of this classical disease model are specified in the cells' `population growth` (which defaults to `birth and death` if omitted, see section 5.4.3). Finally, using the `metabolic modulation` (see section 5.4.2) we specified that `Nitrosomonas_infected` has a metabolic activity reduced by a factor 1/2, compared to `Nitrosomonas` under identical conditions.

To include phage concentrations in graphical simulation output, we specify them as focal by adding the following to the `focals` file:

```
FOCAL_ENVIRONMENT
    phage*
```

Notice that we use the wild card '`*`' to include both `phage_Ns` and `phage_Nb`. Running a simulation using the original script from section 2.2 produces the output illustrated in Fig. 14. Notice the initial grow of both cell species, triggering the proliferation of bacteriophages and the subsequent infection of cells.

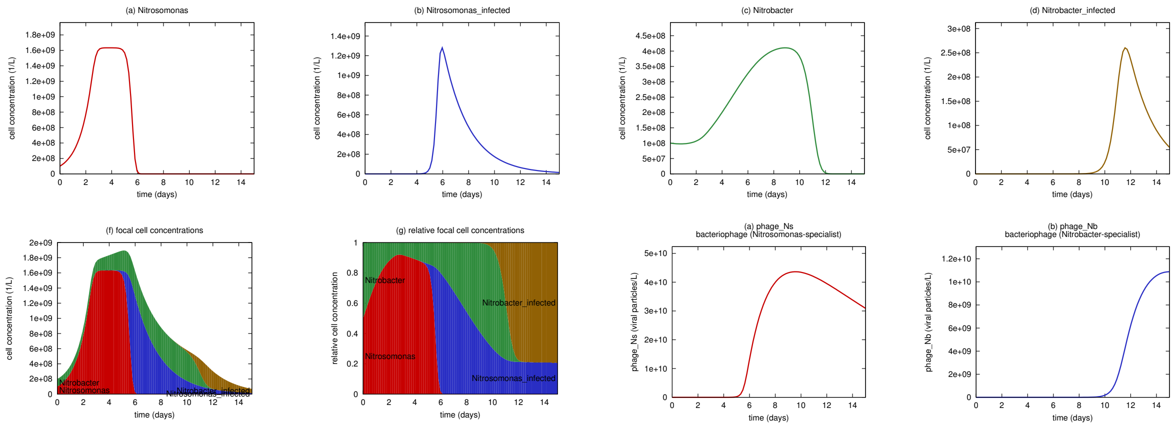

Figure 14: Dynamics of the nitrifying bioreactor model with bacteriophages, described in section 2.8. Top row: Densities of non-infected and infected cell species. Bottom row: Stacked cell concentrations (columns 1 & 2) and bacteriophage concentrations (columns 3 & 4). Excerpts from the output files `cell_concentrations.pdf` and `environment.pdf`.

2.9 Environmental (abiotic) reactions

Some reactions can take place abiotically in the environment, i.e. independent of microbial metabolism. For example, nitrate reduction to ammonium in soils can occur abiotically in the presence of sulfate green rust [29, 28] stoichiometrically as follows:

$$\mathrm{Fe_4^{II}Fe_2^{III}(OH)_{12}(SO_4)_{(s)}} + \tfrac{1}{4}\mathrm{NO_3^-} \rightarrow \mathrm{SO_4^{2-}} + \tfrac{1}{4}\mathrm{NH_4^+} + 2\mathrm{Fe_3O_{4(s)}} + \tfrac{3}{2}\mathrm{H^+}. \tag{5}$$

Experimentally determined rates follow the first-order kinetics

$$\frac{d\left[\mathrm{NH_4^+}\right]}{dt} = k \cdot [\mathrm{Fe(II)}]_{\mathrm{GR}} \cdot \left[\mathrm{NO_3^-}\right], \tag{6}$$

where $k \approx 4.26\ \mathrm{L \cdot mol^{-1} \cdot d^{-1}}$ at 25°C [28]. Let us incorporate this environmental reaction into the original microbial model from section 2.1. Hence, we start with the first version of the model files `metabolites`, `reactions`, `species` and `focals`. We begin by adding the three metabolites, `FeIIGr`, `Fe3O4` and `SO4` to the `metabolites` file:

```
FeIIGr
    description: sulfate green rust
    max passive uptake rate: 0
    max passive export rate: 0
    environmental dynamics:  initial 0.012 flux environmental_production

Fe3O4
    description: iron (II,III) oxide
    max passive uptake rate: 0
    max passive export rate: 0
    environmental dynamics:  initial 0 flux environmental_production
```

```
SO4
    description: sulfate
    max passive uptake rate: 0
    max passive export rate: 0
    environmental dynamics:  initial 0 flux environmental_production
```

Observe that we have specified the `environmental dynamics` of all three metabolites as dynamical, with a rate of change according to `environmental_production`. This tells MCM to update `FeIIGr`, `Fe3O4` and `SO4` concentrations according to the net amounts produced (or consumed) by environmental reactions. Let's also adjust the `environmental dynamics` of `NH4` and `NO3` to account for fluxes from abiotic reactions (in addition to microbial metabolism):

```
NH4
    description: ammonium
    max active uptake rate:  Monod 1.23e-13 2.6e-5
    max active export rate:  unlimited
    max passive uptake rate: 0
    max passive export rate: 0
    environmental dynamics:  initial 1e-4 flux microbial_export and environmental_production

NO3
    description: nitrate
    max active uptake rate:  0
    max active export rate:  unlimited
    max passive uptake rate: 0
    max passive export rate: 0
    environmental dynamics:  initial 0 flux microbial_export and environmental_production
```

Next, let's add protons (`H`) as an additional metabolite:

```
H
    description: protons
    max passive uptake rate: 0
    max passive export rate: 0
    environmental dynamics:  concentration 10^(-7)
```

Note that we assumed a fixed pH of 7 to calculate proton concentrations. More generally, we could have defined pH as an environmental variable that depends, for example, on `NH4` concentrations. To summarize, the `metabolites` file should now contain the following:

```
file_version: 1.3 # do not remove, move or change this line

NH4
    description: ammonium
    max active uptake rate:  Monod 1.23e-13 2.6e-5 # Monod kinetics, requires Vmax and Khalf
    max active export rate:  unlimited
    max passive uptake rate: 0
    max passive export rate: 0
    environmental dynamics:  initial 1e-4 flux microbial_export and environmental_production

NO2
    description: nitrite
    max active uptake rate:  Monod 3.25e-13 2.29e-4
    max active export rate:  unlimited
    max passive uptake rate: 0
    max passive export rate: 0
    environmental dynamics:  initial 0 flux microbial_export

NO3
    description: nitrate
```

```
    max active uptake rate:  0
    max active export rate:  unlimited
    max passive uptake rate: 0
    max passive export rate: 0
    environmental dynamics:  initial 0 flux microbial_export and environmental_production

O2
    description: oxygen
    max active uptake rate:  1e-13
    max active export rate:  unlimited
    max passive uptake rate: 0
    max passive export rate: 0
    environmental dynamics:  concentration 0.217e-3   # 100% saturation with atmosphere

H2O
    description: water
    max active uptake rate:  unlimited
    max active export rate:  unlimited
    max passive uptake rate: unlimited
    max passive export rate: unlimited
    environmental dynamics:  concentration 0

FeIIGr
    description: sulfate green rust
    max passive uptake rate: 0
    max passive export rate: 0
    environmental dynamics:  initial 0.012 flux environmental_production

Fe3O4
    description: iron (II,III) oxide
    max passive uptake rate: 0
    max passive export rate: 0
    environmental dynamics:  initial 0 flux environmental_production

SO4
    description: sulfate
    max passive uptake rate: 0
    max passive export rate: 0
    environmental dynamics:  initial 0 flux environmental_production

H
    description: protons
    max passive uptake rate: 0
    max passive export rate: 0
    environmental dynamics:  concentration 10^(-7)
```

click here for source code

In the `reactions` file, add the following reaction with first-order kinetics:

```
ammonification
   equation: FeIIGr + 0.25*NO3 -> SO4 + 0.25*NH4 + 2*Fe3O4 + 1.5*H
   environmental rate: 4 * 4.26 * FeIIGr * NO3
```

In general, a reaction's `environmental rate` can depend explicitly on time, environmental variables, metabolite concentrations or cell concentrations (see section 5.3.5 for a full list of options). Biologically catalyzed reactions can also include an `environmental rate`, although in our case (nitrification) we choose to neglect it.

Next, let's tell MCM that we find the metabolites `FeIIGr`, `Fe3O4` and `SO4` and the reaction `ammonification` interesting, by modifying the `focals` to the following:

```
file_version: 1.3 # do not remove, move or change this line

FOCAL_METABOLITES
  NH4
  NO2
  NO3
  Fe*
  SO4

FOCAL_REACTIONS
  amo
  nxr
  ammonification

FOCAL_SPECIES
  *
```

click here for source code

Observe that we used the wildcard '*' to designate all iron-compounds and all cell species as focal.

Because biocatalyzed nitrification oxidizes ammonium to nitrate and abiotic ammonification slowly reduces nitrate back to ammonium, nitrogen cycling is expected in the system (as long as sulfate green rust is available as an electron donor). To visualize nitrogen fluxes between reactions at particular time points (e.g. at the end of day 2 and day 14), we use the MCM control variable `plotMetabolicFluxDiagrams` (see section 6 for further options). Concretely, in the original script from section 2.2 we add the following line anywhere prior to the `runMCM` command:

```
set plotMetabolicFluxDiagrams at 2 14
```

Running the modified script produces the output illustrated in Figs. 15 and 16.

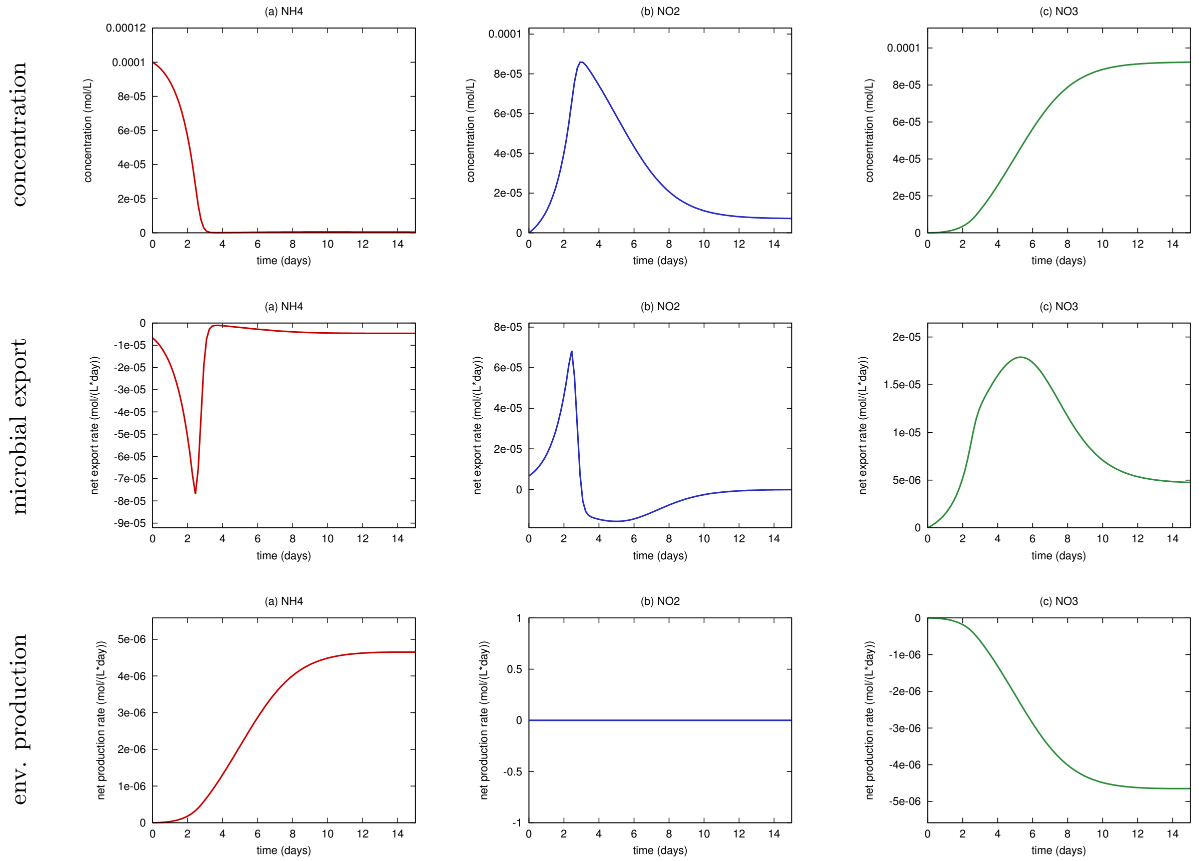

Figure 15: Environmental concentrations (top row), microbial net export rates (center row) and environmental net production rates (bottom row) over time for `NH4`, `NO2` and `NO3` (columns 1–3, respectively), in the nitrifier model with abiotic ammonification (section 2.9). Observe that as `NO3` accumulates, the system shifts from mostly nitrifying to a balance between nitrification and abiotic ammonification. In the latter state, nitrogen cycling is sustained by the presence of reducing sulfate green rust. Excerpts from the output files **`metabolite_concentrations.pdf`**, **`metabolite_net_environmental_production.pdf`** and **`metabolite_net_export_community_wide.pdf`**.

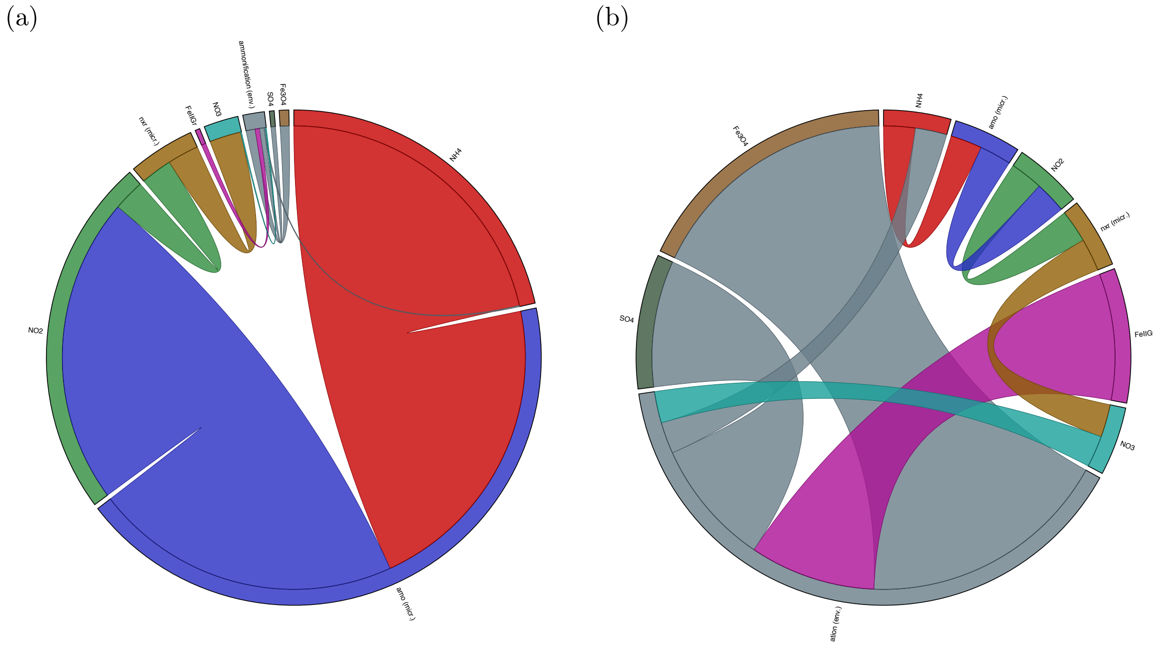

Figure 16: System-wide metabolic fluxes between reactions and metabolites after 2 days (a) and 14 days (b), in the nitrifier model with abiotic ammonification (section 2.9). Chords connect reactants to reactions and reactions to products. Chords are colored according to their origin. Chord widths are proportional to flux rates. Environmental and microbial rates are indicated by "env." and "micr.", respectively. Observe that on day 2 the system is dominated by nitrification, while on day 14 nitrification is balanced by abiotic (environmental) ammonification. Excerpts from the output files `metabolic_flux_diagrams/current_at_time_2.html` and `metabolic_flux_diagrams/current_at_time_14.html`.

3 Installation

3.1 System requirements and dependencies

MCM is mostly self-contained and should run without problems on any typical UNIX-like machine (including at least Mac OS X and Linux). Plotting graphs requires additional free 3rd party software (see section 3.5), the absence of which, however, does not interfere with MCM's computational functions. Note that compiling MCM from the sources (e.g. if a compiled executable is not available for your platform) also imposes additional requirements on the system (see section 3.4). MCM has been successfully compiled and tested on Mac OS 10.5 - 10.8, Ubuntu Linux 8.04 LTS and Fedora Linux 7.

Hardware requirements depend on model size. For example, memory requirements increase linearly with species number and average metabolic model size (number of non-zero entries in the stoichiometric matrix). Computing time increases linearly with species number and polynomially with average cell-model size[6]. A single-species simulation involving 1000 reactions and lasting 1000 time steps will typically take a few seconds on a modern laptop (as of 2014).

3.2 3rd party components

In addition to its own roughly 50 000 lines of code, MCM also includes the following 3rd party code:

- NLopt 2.4.2, an open source nonlinear optimization library [40, 75, 56, 65, 64]. NLopt is distributed under the GNU Lesser General Public License 2.1 or later.

[6]The latter is dictated by the complexity of the simplex algorithm used to solve the linear optimization problem for each species and at each time step.

- lpsolve 5.5.2, an open source linear programming solver [53]. lpsolve is distributed under the GNU Lesser General Public License 2.1 or later.
- D3, a free JavaScript library for data visualization, is distributed under the BSD 3-Clause license.
- Underscore, a free general-purpose JavaScript library, is distributed under the MIT license.

Detailed descriptions and license agreements for the above libraries can be found in their respective subdirectories. No separate installation or configuration is needed for the above libraries, since they come bundled with MCM.

3.3 Installing MCM

Compiled binaries (*executables*) of recent MCM releases might be available for your operating system at the project's website: `http://www.zoology.ubc.ca/MCM` (and might even be included with this manual). If that is the case, simply download the suitable binary and place it in a location of your choice. You might need to adjust the file's execute permissions to be able to run it as a program. On a typical UNIX-like machine, the required terminal commands might look as follows:

```
chmod ugo+x MCM          # make MCM executable by all users
mv MCM /usr/local/bin/   # make MCM accessible system-wide
```

Note that while MCM is mostly a standalone program, it does require certain (free) 3rd party software for graphing, described in detail in section 3.5.

3.4 Compiling MCM from the sources

If a recent binary is not available for your system you will need to compile MCM from the raw C++ sources. Compiling MCM requires a recent version of the GNU C++ compiler (`g++`), by default present on most modern UNIX-like systems. Note that earlier versions of `g++` (<4.2) do not support OpenMP, so MCM binaries compiled with such a compiler will not allow parallel (multithreaded) computations. Mac OS X users, please also see the FAQ in section 14.1.3.

The latest MCM sources are available at the project's website (or might even be included with this manual). If the downloaded file is a compressed `.tar.gz` file, you can unpack it via the terminal, e.g.

```
gunzip -c MCM_1.0.tar.gz | tar xopf -
```

(or by double-clicking on the file on a modern Mac). After unpacking, you should obtain a `source_code` directory containing at least the file `MAKEFILE` and the subdirectory `sources`. In the terminal, change to the `source_code` directory and run `make` to compile:

```
cd MCM_1.0/source_code
make clean
make
```

To compile MCM without support for parallel computing use '`make openmp=0`' instead of '`make`' in the last command.

Attention: The MCM sources include 3rd party code which is also automatically compiled (for a detailed list see section 3.2). Some of the included 3rd party code requires that during compilation the entire path to the MCM directory does not contain any whitespace characters. This may not be obvious from the errors you would otherwise get.

After a long list of warning messages (and potentially a lot of prayer), you should obtain an executable MCM binary. MCM has been successfully compiled with `g++` versions as old as 4.0.1 (released in 2005). Follow the instructions at the beginning of this section to install your freshly created binary to your favorite location. If you run into troubles while installing MCM please consult the FAQ (section 14) or contact MCM's developers (consult the MCM website for contact details).

3.5 Plotting

MCM plots graphics using gnuplot, a free and powerful command-line graphing utility (http://www.gnuplot.info). All data and plotting commands are transparently passed to gnuplot and plots are automatically saved into files. For each plot file, the raw plot data and gnuplot scripts are always saved as separate files (ending with `_data.txt` and `_plot_script.gp`, respectively), allowing the reproduction and manual adjustment of plots (e.g. for publication).

An installation of gnuplot is required for plotting. However, data and plot scripts are still saved even if gnuplot is absent. You can check if MCM was able to locate the correct gnuplot version on your system using the command `checkGnuplot` in the MCM command line (see section 4.1 for details on MCM's command line interface). MCM 1.3 is most compatible with gnuplot 4.6 (the latest release as of September 6, 2014), the sources and installation scripts of which should be included with the MCM package. If this is not the case, you can obtain gnuplot here.

Gnuplot has to be compiled and installed separately from MCM, which is typically done using the command line tools `config` and `make`[7]. On Mac OS X, this would look similar to

```
gunzip -c gnuplot-4.6.5.tar.gz | tar xopf -
cd gnuplot-4.6.5
./configure --with-readline=builtin --enable-qt
make
sudo make install
```

click here for source code

If you run into troubles during compilation or installation, consult the gnuplot FAQ. On Mac OS X, gnuplot can also be installed via the `homebrew` package manager (if available) using the command

```
brew install gnuplot
```

If you already have a gnuplot version other than 4.6 installed, it is recommended that you also install gnuplot 4.6 in a separate location and tell MCM where to find it using the `setGnuplot` command, e.g.

```
# explicitly specify path to gnuplot version 4.6
setGnuplot "/usr/local/bin/gnuplot4.6"
```

Omitting the above command, or calling `setGnuplot ""`, will result in MCM silently trying to locate and use the local gnuplot installation.

If a compatible gnuplot version is available, MCM generates plots in one of the following vector graphics formats:

PDF: Requires one of the three command line programs `epstopdf`, `ps2pdf` or `pstopdf`, all of which are free and at least one of which will typically be installed on modern UNIX-like systems. `epstopdf` can be retrieved at http://ctan.org/pkg/epstopdf. `ps2pdf` is part of the Ghostscript package which can be obtained at http://pages.cs.wisc.edu/~ghost/. `pstopdf` is part of standard Mac OS X installations (as of Mac OS 10.4). If neither `epstopdf`, `ps2pdf` nor `pstopdf` can be found by MCM, plots are saved in postscript (EPS) format instead.

Technical note: Heatmaps might appear blurred when viewed in some PDF/EPS viewers (such as Preview on Mac OS X). If the command line program `eps2eps` is installed, MCM automatically uses it to fix these badly rendered heatmaps.

SVG: Requires nothing but a compatible gnuplot installation. Hence, if PDF plotting is unavailable, this might be a good alternative, especially on Linux machines.

The plot file type is specified via the `plotType` variable, e.g.

```
set plotType SVG    # only other option is 'PDF'
```

[7]This in turn requires that GCC be installed on your system. You might also find pre-compiled gnuplot executables and installers for your operating system online.

4 MCM control

4.1 The MCM command line

MCM is controlled via its own command line interface (see section 4.4 for controlling MCM through scripts). Functions are explicitly invoked via MCM commands (throughout this document shown in `this format`), while their behavior is modified by previously adjusted boolean flags and control variables (shown in `this format`). Comments preceded by the "#" symbol are ignored. MCM's commands can be roughly divided into *householding* commands (e.g. for modifying control variables, getting help, organizing output directories or making file backups) and *functional* commands (involving simulations). Important householding commands are `set` and `unset` for changing control variables and flags and `setod` (and cousins) for changing the current output directory. A full list of householding commands follows below:

`set`: Either followed by the name of a flag to be enabled or followed by the name of a variable and a value to be assigned to the variable, e.g.

```
set parallel                         # this is a flag
                                     # it can only be 'set' or 'unset'
set maxTimeSeriesSize    100         # this is a numerical variable
                                     # it is always set to a particular value
set heatmapColorPalette  rainbow     # this is another variable,
                                     # which can only be set to certain keywords
```

`unset`: Disable a flag, e.g.

```
unset parallel   # disable parallel computation
```

`help`: Display an overview of available flags, macros, variables and commands. If followed by a name, displays an overview only for that particular flag, macro, variable or command. See section 4.2 on the format of syntax overviews.

`state`: Show the current values of all flags, macros and variables. If followed by a name, only shows the value of that particular flag, macro or variable.

`printod`: Print the current output directory. Note that the macro `$od$` also evaluates to the current output directory (see section 4.3 on macros).

`openod`: Open the current output directory in the default file browser.

`changeod`: Change output directory to the path following the command. If the path is a relative one, it is interpreted relative to the current output directory.

`setod`: Change output directory to the path following the command. For example, each of the following four variants result in the same output directory:

```
# variant 1
setod  outputs/example

# variant 2
setod     outputs
changeod  example

# variant 3
setod     outputs/another_example/sub
changeod  ../../example

# variant 4
setod  outputs
setod  $od$/example
```

`changeodnew`: Similar to `changeod`, but the target directory name is extended by a integer that ensures that the new output directory does not yet exist.

setodnew: Similar to **setod**, but the target directory name is extended by a integer that ensures that the new output directory does not yet exist.

saveContext: Save the current values of all flags, macros and variables as a script with the name `context` into the current output directory. If followed by a file path, `saveContext` saves the script to the specified path instead (relative to the current output directory, if the given path is relative).

loadContext: Followed by a path to a script previously saved by `saveContext`. Reverts all flags, macros and variables to the state saved in the script. For example, after the following commands the flag `parallel` will be set:

```
setod          my_output_dir
set            parallel
saveContext    context_initial       # save context to my_output_dir/context_initial
unset          parallel
loadContext    $od$/context_initial   # revert to previously saved context
```

execute: Execute an arbitrary shell command in the current shell working directory, for example

```
execute "mkdir a_new_directory"
```

Separate several commands with semicolons. Beware of the security risks inherent in this functionality.

executeinod: Execute an arbitrary shell command (e.g. `mkdir` or `rm`) in the current output directory. Separate several commands with semicolons. Beware of the security risks inherent in this functionality.

open: Open a file or directory. Beware of the security risks inherent in this functionality.

openinod: Open a file or directory, with the path given relative to the current output directory. For example, the following will create and open the directory `dir1/dir2`:

```
setod        dir1           # set and create output directory
executeinod  "mkdir dir2"   # create sub-directory in output directory
openinod     dir2           # open sub-directory in output directory
```

Beware of the security risks inherent in this functionality.

define: Define custom macros. See section 4.3.

loadScript: Load an MCM script. See section 4.4.

saveScript: Save a copy of the current MCM script into the current output directory, e.g. for archiving purposes. See section 4.4.

seed: Optionally followed by a non-negative integer (e.g. "`seed 1362`"), in which case it initializes MCM's random number generator to the given seed. Otherwise, randomizes the seed using the computer's internal clock.

checkGnuplot: Check version and location of gnuplot executable used by MCM. See section 3.5 for more details.

setGnuplot: Explicitly point MCM to the installation path of gnuplot. If this is empty, MCM tries to automatically locate gnuplot. See section 3.5 for more details.

saveMCmodel: Save a copy of the MC model configuration directory (as defined by the variable `MCmodelDir`) to the current output directory.

saveData: Save a copy of the data directory (as defined by the variable `MCdataDir`) to the current output directory, e.g. for archiving purposes (see section 7.2 on using data).

modelSyntax: Print an overview of the MC model configuration syntax.

expandMCmodelTemplates: Expand any templates in the MC model directory. Existing model files will not be replaced. See section 5.9.

`expandMCmodelTemplatesReplace`: Expand any templates in the MC model directory. Existing model files will be replaced without warning. See section 5.9.

`quit`: Quit MCM. This produces no further output.

A full list of functional commands, further explained in the remaining document, follows below:

`runMCM`: Run a single simulation and evaluate results against available data. See sections 6 and 7.

`RRMCM`: Run multiple simulations with randomly sampled parameters, evaluating results against available data. See sections 5.7, 6 and 7.

`fitMCM`: Fit free model parameters to data. See section 7.3.

`LSAMCM`: Perform local sensitivity analysis of the model. See section 8.1.

`GSAMCM`: Perform global sensitivity analysis of the model. See section 8.1.

`LSAMCM_LL`: Perform local sensitivity analysis of the log-likelihoods of model predictions. See section 8.2.

`GSAMCM_LL`: Perform global sensitivity analysis of the log-likelihoods of model predictions. See section 8.2.

`UAMCM`: Perform uncertainty analysis of an MC model. See section 9.

All command, flag, macro and control variable names are case sensitive, i.e. `QuiT` is not valid (but `quit` is). File paths given as values to a control variable name must be enclosed in quotes if they contain any whitespace.

4.2 Reading syntax overviews

MCM (and this user guide) provides simple overviews of the input syntax required for various commands and model configurations. These overviews are in turn shown in a particular format, which uses special brackets to differentiate between input structures. More precisely, curly brackets “`{}`” enclose a comma-separated list of alternative blocks, of which exactly one needs to be present. Angular brackets “`<>`” enclose the unformatted description (not to be taken literally) of a block. Everything outside of brackets is to be taken literally. For example,

```
set {<variable name> <some value>, <flag name>}
```

describes the general syntax of the `set` command (explained in section 4.1). Rectangular brackets “`[]`” enclose an optional block that can be either present or absent. Two dots “`..`” signify an optional list continuation (i.e. zero or more blocks similar to the preceding one). For example,

```
[{dog, cat, <a plant name>, <a plant name> <another plant name> ..}]
```

represents a block that can either be empty, “`dog`”, “`cat`” or an arbitrary space-separated list of plant names. Round brackets “`()`” enclose a comma-separated list of blocks, any non-empty subset of which can be present (in any order). For example,

```
A (B, C)
```

can either be “`A B`”, “`A C`”, “`A B C`” or “`A C B`”. To see the syntax of all MCM commands and control variables, call `help` in the MCM command line. To see the syntax of a particular MCM command or control variable, call `help` followed by the name of the command or control variable (e.g. “`help fit_objective`”).

4.3 MCM command line macros

Simple text macros can be defined using the `macro` command and evaluated thereafter by enclosing their name in “`$`” symbols. For example, the following commands set the MC model configuration directory to “`MC models/bioreactor`”:

```
macro  parent:MC models     # general syntax: define <name>=<value>
macro  child:bioreactor
set    MCmodelDir "$parent$/$child$"
```

The macros today (evaluating to the current date), now (evaluating to the current date and time) and od (evaluating to the current output directory) are reserved. For example, the following command sets the output directory to "simulations/2014.09.06_15.39.49.863" (at the time of this writing):

```
setod "simulations/$now$"
```

The current value of a macro can be viewed by calling state, followed by the macro name. See section 5.13.1 for macro naming rules. Command line macros also apply to MC models, and provide a way to modify MC models through the MCM command line (section 5.8).

4.4 MCM scripts

MCM is meant to be primarily controlled through human-readable scripts with a simple syntax. This facilitates archiving, task automation, high-throughput execution of multiple simulations and incorporation into other computational pipelines (e.g. through automatic MCM script generation). Scripts can be loaded using the command loadScript (followed by the script path), or by providing the script path as an optional argument when calling MCM from the terminal, e.g. as

```
./MCM my_favourite_script
```

Any MCM commands can be included in a script. In fact, scripts can call any number of other scripts. Once a script is finished executing, control returns to the calling script or command line, unless the script contains the quit command. The only command available exclusively in script mode is saveScript, which saves a backup of the script into the current output directory. Scripts can contain comments, preceded by the "#" symbol. Excessive white space is ignored. A basic MCM script might look as follows:

```
seed # initialize random generator seed

# set custom gnuplot installation path (or leave blank for automatic detection)
setGnuplot "/usr/local/bin/gnuplot4.6"

# specify MC model configuration directory
set MCmodelDir "MC_models/bioreactor/"

# create a new non-existing output directory
setodnew "simulations/run_"

saveScript    # save a copy of this script to the current output directory
saveMCmodel   # save a copy of the MC model to the current output directory

# other MCM commands that actually calculate something
..

quit
```

click here for source code

Note that scripts are loaded sequentially, so any commands or modifications of options, flags or macros will only have an effect on following MCM commands. If an error is encountered in a script, the execution of the remaining script halts and control is returned to the calling script or command line.

5 Defining a microbial community model

A microbial community (MC) model is defined by (i) an arbitrary number of metabolites, (ii) an arbitrary number of (reversible or irreversible) chemical reactions between any of the metabolites and (iii) an arbitrary number of (cellular) species, each species being able to perform any subset of the reactions and exchange any of the metabolites with its environment. Optionally, an additional set of numerical environmental variables (such as light intensity, temperature or pH) can also be included in the model, for example to model light-limitation of photosynthesis.

It is also possible to define a list of model objects (i.e. metabolites, reactions and species) that one wishes to particularly focus on in the analysis. Whether a metabolite, species or reaction is focal or not does only affect the amount of output produced by MCM but has no effect on the microbial community dynamics. This is very useful for large-scale metabolic models that can comprise several hundreds of metabolites and reactions [1, 44], only a few of which might be of real interest.

Environmental variables, metabolites, reactions, species and focals need to be defined in designated text files of a particular format, explained in detail in sections 5.1, 5.2, 5.3, 5.4 and 5.6 below. An overview of the MC model configuration syntax can be obtained using the command `modelSyntax`. The syntax is human readable and fairly easy to understand through the snippets given below (also see the complete examples in sections 10, 11 and 12). All configuration files need to be located in a single *MC model configuration directory*, to which MCM needs to be pointed using the `MCmodelDir` control variable, e.g. as follows

```
set MCmodelDir "microbial_community_models/bioreactor"
```

In addition, MC models can include *symbolic* (or *abstract*) parameters, with a predefined range, default value and optional probability distribution. Symbolic parameters play a central role in parameter estimation, sensitivity analysis and uncertainty analysis, and are elaborated on in section 5.7.

Technical note: All physical quantities specified in the model configuration are assumed to be given in specific physical units, summarized in section 5.13.4.

5.1 Environmental variables

5.1.1 Introduction

MCM allows the inclusion of any number of environmental variables. Environmental variables can be (i) *any* explicit function of time, metabolite concentrations and other environmental variables, (ii) interpolated from available data, (iii) a stochastic process or (iv) dynamic, whose rate of change is any of (i), (ii) or (iii). Environmental variables, in turn, can be specified to influence metabolite uptake/export limits of cells, reaction rate limits, environmental metabolite dynamics or even other environmental variables. For example, pH in a bioreactor might be set to a measured profile and in turn used as a parameter in ammonia-ammonium dissociation equilibria. Alternatively, pH might be specified as a function of organic acid concentrations, and in turn influence the growth rate of cells. Similarly, light might be defined as a periodic function of time which in turn influences maximum photosynthesis rates in photosynthesizing cells. Furthermore, environmental variables might be defined as convenient summary variables explicitly depending on the system's state. For example, the concentration ratio of two particular metabolites, if of interest, can be defined as an environmental variable the temporal profile of which can be included in a simulation's output.

5.1.2 Specifying environmental variables

All environmental variables need to be defined in the optional `environment` configuration file, located in the MC model directory. Each environmental variable definition consists of a unique name on a single line, followed by several key:value pairs given on consecutive lines (in any order):

- `description` (optional)
- `units` (optional): Physical units, for plotting purposes

- dynamics (required): Temporal profile of the variable
- sign (optional): Sign to be enforced
- scaling (optional): Typical scaling (linear or logarithmic), for plotting purposes
- error model (optional): Error structure to be used for data comparison

Any excessive white space, empty lines or comments preceded by the '#' symbol are ignored. The first non-empty line in the file must indicate the file version (1.3). An example environment file is shown below (see below for an elaboration):

```
file_version: 1.3 # do not remove, move or change this line

light
   description: total energy of visible spectrum
   units:    W/m^2
   scaling:  logarithmic  # plot on a logarithmic scale
   dynamics: value 6 and periodic 5 1 0.5 # light intensity oscillates diurnally
                                          # around mean = 6, with amplitude = 5
                                          # and first peak after half a day

pH
   dynamics: value interpolate "data/Nitrifiers/pH"  # use measured profile
                                                     # (interpolate time series data)

salinity
   units:    PSU
   sign:     positive   # make sure salinity stays positive
   dynamics: value 32 and OU 5 20   # salinity fluctuates as an OU stochastic process
                                    # with mean = 32, standard deviation = 5
                                    # and correlation time = 20

# temperature increases with light
# and drops to freezing point in the absence of light
temperature
   units:    K
   dynamics: initial 273 rate 15*light - 0.1*(temperature-273)
```

click here for source code

An environmental variable's description, units and scaling can be empty or omitted, and are only relevant to graphical output style. sign can either be omitted, or set to any (default), negative or positive. These choices will affect wether the sign of the environmental variable will be enforced during simulations, e.g. in the case of numerical inaccuracies. error model can either be omitted, or set to none, normal (default) or logNormal. These choices affect the assumed error structure when comparing the environmental variable's time course to experimental data (e.g. for model fitting, see section 7).

5.1.3 Dynamics

An environmental variable's dynamics can either specify a rate of change, or an explicit temporal profile. More precisely, dynamics may be one of the following:

(i) an arbitrary function of metabolite and cell concentrations, other environmental variables and time (t), possibly extended by an OU stochastic process [91, 27], a log-OU stochastic process [19] or a periodic (sinusoidal) component. The general syntax is

```
value <list of additive components separated by " and ">
```

where each of the additive components can be one of (without duplication):

```
OU       <standard deviation> <correlation time>
logOU    <positive mean> <standard deviation> <correlation time>
periodic <amplitude> <period> <phase lag>
<a custom function of time (t), metabolite concentrations,
```

```
                                      environmental variables
                                      and cell concentrations>
```

Examples are given below:

```
value 0
value 10 and periodic 1 365 60   # annually oscillating concentration
                                 # mean 10, amplitude 1 and maximum on day 60
value 1e-6*exp(-pH)*2^(-t/10)  # depends exponentially on pH
                               # and decays with time (half-life time = 10 days)
value 1e-6 and OU 1e-7 10   # fluctuates as an OU process
                            # around average=1e-6, with a standard deviation=1e-7
                            # and correlation time=10
value logOU 1e-6 1e-7 10  # fluctuates as a log-OU process
                          # around average=1e-6, with a standard deviation=1e-7
                          # and correlation time=10
value logOU 1e-6 1e-7 10 and periodic 1 365 60 # superimpose stochastic
                                               # and periodic
value Nitrosomonas*3e-13 + Nitrobacter*4e-13   # total nitrifier biomass
```

(ii) a piecewise linear interpolation of a given (e.g. measured) time series taken from a separate file, e.g.

```
value interpolate "data/measured_O2.txt"
```

File paths must either be absolute (e.g. `/users/mikey_mouse/my_time_series`) or given relative to the MC model directory (e.g. `../my_time_series`). See section 14.3.6 on format requirements for time series data files. A combination of (i) and (ii) is also possible, e.g.

```
value interpolate "measured_O2.txt" and OU 10 5 # superimpose artificial noise
                                                # on the measured data
```

(iii) a dynamical variable whose rate of change can depend, among others, on time (`t`), metabolite concentrations, cell concentrations, environmental variables and biomass death rate. The general syntax is

```
initial <initial concentration> rate <additive flux components separated by " and ">
```

where each of the rate components can be one of (without duplication):

```
OU       <standard deviation> <correlation time>
logOU    <positive mean> <standard deviation> <correlation time>
periodic <amplitude> <period> <phase lag>
biomass_death <change per (g dry weight dead biomass)/L>
<a custom function of time (t),
                      metabolite concentrations,
                      environmental variables,
                      cell concentrations,
                      reaction Gibbs free energies,
                      cell growth, birth and death rates,
                      environmental reaction rates,
                      environmental metabolite production rates,
                      community-wide reaction rates,
                      community-wide metabolite export rates,
                      cell-specific reaction rates,
                      cell-specific metabolite fluxes>
```

Examples are given below for the environmental variable TSS (total suspended solids):

```
initial 0 rate biomass_death 0.06 # TSS increases by 0.06 per g dry weight dead biomass
initial 100 rate biomass_death 0.06 and -0.1*TSS # account for sedimentation

initial 0 rate logOU 1 0.2 10   # rate of change is a log-OU stochastic process
                                # with mean=1, standard deviation=0.2
                                # and correlation time=10
```

```
initial 100 rate biomass_death 0.06 and logOU 1 0.2 10 and -0.1*TSS

initial 0 rate rate_pc.amo.Nitrosomonas * Nitrosomonas * DeltaG.amo
```

See table 1 for a complete list of model variables that can be included in the custom `rate` function. See section 5.13.2 for the general structure of custom functions.

Table 1: Model variables that can be included in an environmental variable's `rate` or a metabolite's `flux`, together with required prefixes (sections 5.1.3 and 5.2.2). The abbreviation "env." stands for environmental (i.e. extracellular), "cw." for community-wide, "pc." for per-cell.

variable kind	required prefix	example
time	*none*	`t`
environmental variable	*none*	`temperature`
cell concentration	*none*	`Ecoli`
cell growth rate (per capita)	`growth_rate.`	`growth_rate.Ecoli`
cell birth rate (per capita)	`birth_rate.`	`birth_rate.Ecoli`
cell death rate (per capita)	`death_rate.`	`death_rate.Ecoli`
cell mass	`cell_mass.`	`cell_mass.Ecoli`
gene concentration	`concentration.`	`concentration.amo`
gene concentration (dead+alive)	`concentration_da.`	`concentration_da.amo`
reaction rate (cw.)	`rate_cw.`	`rate_cw.amo`
reaction rate (per cell, single species)	`rate_pc.`	`rate_pc.amo.Nitrosomonas`
reaction rate (env.)	`rate_env.`	`rate_env.amo`
metabolite concentration	*none*	`NO2`
net metabolite export (cw.)	`net_export_cw.`	`net_export_cw.NO2`
net metab. export (per cell, single species)	`net_export_pc.`	`net_export_pc.NO2.Nitrosomonas`
net metab. production (env.)	`net_production_env.`	`net_production_env.NO2`
reaction Gibbs free energy (ΔG)	`DeltaG.`	`DeltaG.amo`
reaction std. Gibbs free energy (ΔG_0)	`DeltaG0.`	`DeltaG0.amo`

(iv) a dynamical variable whose rate of change is a piecewise linear interpolation of a given (e.g. measured) time series, e.g.

```
initial 0 rate interpolate "data/TSS_flux_through_pump.txt"
```

Note that the file path must either be absolute or given relative to the MC model directory. A combination of (iii) and (iv) is also possible, e.g.

```
initial 0 rate interpolate "TSS_flux.txt" and biomass_death 0.06
```

(v) the acid/base/acid+base component of a conjugate acid-base pair. This requires the specification of a paired metabolite and an acid dissociation constant, e.g.

```
base_of_acid HNO2 7.1e-4                # the dissociated amount corresponding to HNO2
acid_of_acid_plus_base HNO2_NO2 7.1e-4  # the undissociated fraction of HNO2+NO2
```

See section 5.2.2 on metabolite dynamics for more examples. In all cases, `pH` must be defined as an environmental variable. If `temperature` is also defined as an environmental variable (with units °K, °C or °F), then acid dissociation constants are corrected for non-standard temperatures.

Circular dependencies among environmental variables and metabolite concentrations are not allowed and will be detected automatically.

5.1.4 Temperature and pH as special cases

In general, environmental variables can have any meaning and their proper (or improper) use (e.g. for regulating reaction rates and metabolite uptake kinetics) is the responsibility of the user. However, if an environmental variable is named "`pH`", it is interpreted as such and is used by MCM to calculate acid-base dissociation equilibria between metabolites whenever applicable (see option v in section 5.1.3). Similarly, if `temperature` is defined as an environmental variable (with units `C`, `K` or `F`), it is used to correct acid-base

dissociation constants at non-standard conditions, as well as to calculate Gibbs free energies of reactions (section 5.3.2).

5.2 Metabolites

All considered metabolites need to be defined in the `metabolites` configuration file, located in the MC model directory. Each metabolite definition (*record*) consists of a unique metabolite name on a single line (see section 5.13.1 for naming rules), followed by several key:value pairs given on consecutive lines (in any order):

- `description` (optional)
- `active uptake objective` (optional)
- `active export objective` (optional)
- `max active uptake rate` (optional)
- `max active export rate` (optional)
- `max passive uptake rate` (required)
- `max passive export rate` (required)
- `DeltaGf0` (optional): Standard Gibbs free energy of formation (kJ/mol)

Any excessive white space, empty lines or comments preceded by the '#' symbol are ignored. The first non-empty line in the file must indicate the file version (1.3). An example `metabolites` file is given below (see thereafter for details):

```
file_version: 1.3 # do not remove, move or change this line

O2
   description: dissolved oxygen
   active uptake objective: 0
   active export objective: 0
   max active uptake rate: Monod 1.2e-13 3.9e-6 # Monod uptake kinetics, needs Vmax and Khalf
   max active export rate: unlimited
   max passive uptake rate: custom 1e-15 * O2   # proportional to O2 concentration
   max passive export rate: 0

   # oxygen concentration given as a periodic function of time (t, in days)
   environmental dynamics: concentration 280e-6 + 10*sin(2*pi*t)



NH3_NH4
   description: ammonia+ammonium

   # linear coefficients for active cell-environment transport in FBA objective function
   # (g dry-weight biomass per mol transported)
   active uptake objective: 0
   active export objective: 0

   # maximum uptake rate (mol/(cell*day)) depends on environmental NH3 concentration (mol/L)
   max active uptake rate: custom 1.2342e-13 * NH3/(NH3 + 26e-6)

   # if a cell has an active export mechanism, assume unlimited capacity
   max active export rate: unlimited

   # assume no passive (i.e. without explicit mechanism) uptake nor export
   max passive uptake rate: 0
   max passive export rate: 0
```

```
   # initial concentration changes according to microbial uptake/export
   environmental dynamics: initial 0.916e-3 flux microbial_export



NH3
   description:   # description can also be blank
   active uptake objective: 0
   active export objective: 0
   max active uptake rate:  1e-2
   max active export rate:  unlimited
   max passive uptake rate: 0
   max passive export rate: 0
   DeltaGf0:                -26.7  # standard Gibbs free energy of formation

   # NH3 concentration is determined by acid dissociation of ammonia
   # and therefore also depends on pH
   environmental dynamics: base_of_acid_plus_base NH3_NH4 5.62e-10
```

click here for source code

5.2.1 Metabolite transport kinetics

In the previous example you will notice that maximum uptake/export rates can be defined in several ways:

- As `unlimited` (i.e. unconstrained).
- As a non-negative numerical constant.
- According to `Monod` uptake kinetics, by specifying the maximum transport rate at saturation ("V_{max}") and half-saturation concentration ("K_{half}"), e.g.

```
   Monod 1e-14 1e-6
```

- As a `custom` function of time (`t`), metabolite and cell concentrations and environmental variables, e.g.

```
   custom 1e-3/(1e-3+NO2) * NH3*1e-13/(NH3+1e-6)  # Monod NH3 uptake kinetics
                                                  # with NO2 inhibition
   custom 1e-14*exp(pH-5)*NH3                     # 1st-order NH3 uptake kinetics
                                                  # with inhibition at low pH
```

  See section 5.13.2 for allowed custom functions.

Active metabolite transport limits apply to species for which the presence of an active transport mechanism is explicitly specified (see section 5.4), while *passive* metabolite transport limits apply to all other species. To prevent exchange of a metabolite in cells for which no active transport mechanism has been specified, set the `max passive uptake/export rate` to `0`.

It is possible to define multiple versions of active metabolite transport limits, for example corresponding to different alleles of the same transporter enzyme or different transport mechanisms. For example, the following record defines a metabolite with three different versions of `max active uptake rate`.

```
NH3
   max active uptake rate: 1e-2                 # wild type version
   max active uptake rate allele01: 1e-3        # alternative version 1
   max active uptake rate allele02: unlimited   # alternative version 2
   max active export rate: unlimited
   max passive uptake rate: 0
   max passive export rate: 0
```

Active transport versions follow similar naming conventions as metabolite names (section 5.13.1) and are distinguished using the "." separator, e.g. `NH3` (wild type), `NH3.allele01` and `NH3.allele02`. This allows

for inter-specific variations of uptake constraints for the same metabolites (see section 5.4 for details).

5.2.2 Environmental metabolite dynamics

The dynamics of environmental metabolite concentrations are specified via `environmental dynamics`, using a syntax similar to that of an environmental variable (section 5.1.3). More precisely, `environmental dynamics` can be one of the following:

(i) an arbitrary function of other metabolite and cell concentrations, environmental variables and time (`t`), possibly extended by an OU stochastic process, a log-OU stochastic process or a periodic (harmonic) component. The general syntax is

```
concentration <list of additive components separated by " and ">
```

where each of the additive components can be one of (without duplication):

```
OU       <standard deviation> <correlation time>
logOU    <positive mean> <standard deviation> <correlation time>
periodic <amplitude> <period> <phase lag>
<a custom function of time (t), metabolite concentrations,
                                environmental variables
                                and cell concentrations>
```

Examples are given below:

```
concentration 0
concentration HNO2 + NO2  # sum of dissociated and undissociated nitrous acid
concentration 10 and periodic 1 365 60   # annually oscillating concentration
                                         # mean 10, amplitude 1 and maximum on day 60
concentration 1e-6*exp(-pH)*2^(-t/10)  # concentration depends exponentially on pH
                                       # and decays with time (half-life time = 10 days)
concentration 1e-6 and OU 1e-7 10   # concentration fluctuates (as an OU process)
                                    # around average=1e-6, with a standard deviation=1e-7
                                    # and correlation time=10
concentration logOU 1e-6 1e-7 10  # concentration fluctuates as a log-OU process
                                  # around average=1e-6, with a standard deviation=1e-7
                                  # and correlation time=10
concentration logOU 1e-6 1e-7 10 and periodic 1 365 60 # superimpose stochastic
                                                       # and periodic component
```

(ii) a piecewise linear interpolation of a given (e.g. measured) time series taken from a separate file, e.g.

```
concentration interpolate "data/measured_O2.txt"
```

Note that file paths must either be absolute or given relative to the MC model directory. See section 14.3.6 on time series data file format requirements. A combination of (i) and (ii) is also possible, e.g.

```
concentration interpolate "measured_O2.txt" and OU 10 5 # superimpose artificial noise
                                                        # on the measured data
```

(iii) a dynamical variable whose rate of change (`flux`) can be an explicit function of, among others, time (`t`), metabolite concentrations, cell concentrations, environmental variables, reaction Gibbs free energies and others (see table 1 for a complete list of model variables that can be included). Microbial uptake/export, production/consumption by environmental (abiotic) reactions (section 5.3.5) as well as biomass recycling can also be included. The general syntax is

```
initial <initial concentration> flux <additive flux components separated by " and ">
```

where each of the flux components can be one of (without duplication):

```
OU       <standard deviation> <correlation time>
logOU    <positive mean> <standard deviation> <correlation time>
periodic <amplitude> <period> <phase lag>
microbial_export
```

```
environmental_production
biomass_death <mol of metabolite released per g dry weight dead biomass>
<a custom function of time (t),
                      metabolite concentrations,
                      environmental variables,
                      cell concentrations,
                      reaction Gibbs free energies,
                      cell growth, birth and death rates,
                      environmental reaction rates,
                      environmental metabolite production rates,
                      community-wide reaction rates,
                      community-wide metabolite export rates,
                      cell-specific reaction rates,
                      cell-specific metabolite fluxes>
```

Fluxes into the system are counted positive. Examples are given below for the metabolite NH3:

```
initial 0   flux microbial_export and environmental_production

initial 280 flux biomass_death 0.06 # 0.06 mol released per g dry weight dead biomass

initial 0   flux 2e-3 and periodic 1e-3 365 0    # annually oscillating influx
                                                 # peak influx 3e-3 on day 0
                                                 # lowest influx 1e-3 on day 182.5

initial 0   flux logOU 1 0.2 10 # influx is a log-OU stochastic process
                                # with mean=1, standard deviation=0.2
                                # and correlation time=10

initial 100 flux microbial_export and -0.1*NH3 # runoff proportional to concentration

initial 100 flux -light*NH3*O2   # degradation by light
                                 # but only in the presence of oxygen

initial 280 flux microbial_export and biomass_death 0.06 and logOU 1 0.2 10 and -NH3/10
```

See section 5.13.2 for allowed custom functions.

(iv) a dynamical variable whose rate of change is a piecewise linear interpolation of a given (e.g. measured) flux time series, e.g.

```
initial 0 flux interpolate "data/O2_flux_through_pump.txt"
```

Note that the file path must either be absolute or given relative to the MC model directory. A combination of (iii) and (iv) is also possible, e.g.

```
initial 0 flux interpolate "fertilization.txt" and microbial_export and biomass_death 0.06
```

(v) the acid/base/acid+base component of a conjugate acid-base pair. Requires the specification of a paired metabolite and an acid dissociation constant at standard temperature (25°C), e.g.

```
base_of_acid HNO2 7.1e-4               # environmental dynamics for NO2
base_of_acid_plus_base HNO2_NO2 7.1e-4 # environmental dynamics for NO2
acid_of_base NO2 7.1e-4                # environmental dynamics for HNO2
acid_of_acid_plus_base HNO2_NO2 7.1e-4 # environmental dynamics for HNO2
acid_plus_base_of_base NO2 7.1e-4      # environmental dynamics for HNO2_NO2
acid_plus_base_of_acid HNO2 7.1e-4     # environmental dynamics for HNO2_NO2
```

In all of these cases, pH must be defined as an environmental variable (section 5.1.4). If temperature is also defined as an environmental variable (in °C, °K or °F), then acid dissociation constants are corrected for non-standard temperatures.

Circular dependencies among environmental variables and metabolite concentrations are not allowed and will be detected automatically.

5.3 Reactions

Reactions are defined in the `reactions` configuration file, located in the MC model directory. Each reaction definition consists of a unique reaction name on a single line (see section 5.13.1 for naming rules), followed by several key:value pairs given on consecutive lines (in any order):

- `description` (optional)
- `equation` (required)
- `max forward rate` (optional, defaults to `unlimited`)
- `max reverse rate` (optional, defaults to `unlimited`)
- `DeltaG0` (optional): Standard Gibbs free energy (kJ/mol)
- `objective` (optional, defaults to `0`)
- `environmental rate` (optional, defaults to `0`): Extracellular reaction rate ($\text{mol}/(\text{L} \cdot \text{day})$)

Excessive whitespace, empty lines and comments preceded by the "#" symbol are ignored. The first non-empty line in the file must indicate the file version (1.3). An example `reactions` file with 2 reactions is given below:

```
file_version: 1.3 # do not remove, move or change this line

biomass_Nitrosomonas
   description:      Nitrosomonas biomass synthesis via ATP consumption
   equation:         15 ATP + 12 NADH + 0.2 protein + 5 CO2 -> 15 ADP + 10 NAD + 15 Pi + 4 O2
   max forward rate: unlimited
   max reverse rate: 1e-10      # reversible reaction
   objective:        113        # g dry-weight biomass per mol reaction

amo
   description:      ammonium oxidation to nitrite
   equation:         NH4 + 1.5 O2 -> NO2 + H2O
   max forward rate: 1e-14 inhibited_by NO2 1e-6
   max reverse rate: 0          # non-reversible reaction
   DeltaG0:          -215.8     # Standard Gibbs free energy
   objective:        0
```

click here for source code

The `description` of a reaction can be left blank or omitted. `equation` and `max forward/reverse rates` are explained in more detail below.

5.3.1 Chemical equation

The general format for chemical equations is

```
<list of reactants> -> <list of products>
```

as demonstrated in the example above. At least one metabolite needs to be included in the reaction. All involved metabolites need to be defined in the `metabolites` file as described in section 5.2. Reactants are to be separated by single "+" signs, and may optionally be preceded by a numerical stoichiometric coefficient. The same holds for products. Stoichiometric coefficients and metabolite names need to be separated either by empty space (" ") and/or an asterisk ("*"). Examples of valid and invalid chemical equations are given below:

```
C6H12O6 + 6 O2 -> 6 CO2 + 6 H2O          # valid
C6H12O6 + 6 O2 -> 6 CO2 + 6H2O           # invalid
1   *   C6H12O6 + 6 O2-> 6 CO2 + 6*H2O   # valid
0.1 protein + 0.5 DNA + 0.01 lipids ->   # valid
X + Y + E -> Z + E                       # valid
->                                       # invalid
```

To define bidirectional reactions, do not use "<->". Instead, use a positive value for `max reverse rate`.

5.3.2 Gibbs free energy

The Gibbs free energy of biocatalyzed redox reactions plays a central role in microbial spatial zonation [6] and has been quantitatively related to microbial growth rates [73]. The Gibbs free energy of any reaction is given by

$$\Delta G = \Delta G_0 + RT \ln Q, \tag{7}$$

where ΔG_0 is the standard Gibbs free energy of the reaction, R is the gas constant, T is the temperature and Q is the reaction quotient [18].

MCM supports the incorporation of reaction energetics in microbial metabolism, by allowing reaction rate limits or objective coefficients to depend on ΔG (sections 5.3.4 and 5.3.3). MCM calculates a reaction's ΔG according to Eq. (7), using the current metabolite concentrations, current temperature and the reaction's ΔG_0. A reaction's ΔG_0 can be specified using the reaction's `DeltaG0` attribute. If the latter is omitted, MCM attempts to calculate ΔG_0 using the standard Gibbs free energies of formation (`DeltaGf0`, see section 5.2) of all involved metabolites.

Technical note: In order to use a reaction's `DeltaG` in an MC model, `temperature` must be defined as an environmental variable in one of the units °K, °C or °F.

5.3.3 Reaction rate limits

Cell-specific reaction rate limits (in either direction) are specified through the `max forward rate` and `max reverse rate` values. These can be any of the following:

- `unlimited` (i.e. unconstrained). This is the default if unspecified.
- A non-negative number, optionally followed by a list of limiting and/or inhibiting metabolites and corresponding half-saturation/half inhibition constants, e.g.

```
1e-14
1e-14 inhibited_by NH2OH 1e-6
1e-14 inhibited_by NH2OH 1e-6 inhibited_by NO2 1e-7 limited_by O2 1e-5
```

  Multiple limitations and inhibitions are combined in a multiplicative manner. Hence, the last case corresponds to a maximum reaction rate given by

$$10^{-14} \times \frac{10^{-6}}{10^{-6} + [\mathrm{NH_2OH}]} \times \frac{10^{-7}}{10^{-7} + [\mathrm{NO_2^-}]} \times \frac{[\mathrm{O_2}]}{[\mathrm{O_2}] + 10^{-5}}. \tag{8}$$

- A `custom` function of metabolite concentrations, environmental variables, cell concentrations, time (`t`) and the reaction's Gibbs free energy (`DeltaG`, if available). Examples follow below:

```
# inhibited by salinity and light
custom 1e-14*exp(-salinity)*exp(-light/10)

# reaction deactivated at NH3 concentrations below 1e-6
custom 1e-14*theta(NH3 - 1e-6)

# Limited by thermodynamic potential factor [LaRowe et al. 2012]
custom 1e-14/(exp((DeltaG + 96.5*0.12)/(8.3e-3*temperature)) + 1)
```

  See section 5.13.2 for allowed custom functions.

It is also possible to define multiple versions of reaction rate limits, e.g. corresponding to different alleles of the same enzyme. For example, the following record defines a reaction with three different versions of `max forward rate`:

```
amo
   equation:                    NH3_NH4 + O2 + Q8H2 -> NH2OH + H2O + Q8
   max forward rate:            1e-14 inhibited_by NH2OH 1e-6    # wild type
   max forward rate order1:     custom 1e-14 * NH2OH             # alternative version 1
   max forward rate constant:   1e-14                            # alternative version 2
   max reverse rate:            0
```

Reaction rate limit versions follow similar naming conventions as reaction names (section 5.13.1) and are distinguished to using the "." separator, e.g. `amo` (wild type), `amo.order1` and `amo.constant`. This allows for inter-specific variations of reaction rate constraints (see section 5.4 for details).

5.3.4 Objective coefficients

The `objective` of a reaction is its contribution to biomass synthesis per intracellular reaction flux (g dry weight yield per mol). It can be any explicit function of time (`t`), metabolite and cell concentrations, environmental variables and the reaction's Gibbs free energy (`DeltaG`, if available). It's syntax is similar to a cell's `metabolic modulation` (section 5.4.2). Examples of an `objective` are given below:

```
# constant objective coefficient
objective: 4.2

# Monod-function of NH3 concentration with half-saturation 0.1
objective: 1 limited_by NH3 0.1

# proportional to the reaction's Gibbs free energy
objective: custom 0.02 * DeltaG
```

Omitting a reaction's `objective` is equivalent to setting it to zero. Conventionally, FBA objectives are proportional to the flux through a single formal *biomass reaction* (as exemplified above) [93, 38, 25]. In that case, all other reactions have a zero objective coefficient but are required for the synthesis of biomass precursors.

Technical note: In certain circumstances a reaction's `DeltaG` can be $+\infty$ or $-\infty$ (e.g. if some products or reactants are absent, respectively). In these cases MCM may be unable to calculate cell metabolism, since infinite objective coefficients can lead to undefined FBA problems. It is therefore recommended to avoid such singularities using the functions `max`, `min`, `escapeInf`, `escapeInf2`, `escapeNAN` (see section 5.13.2). For example, `escapeInf2(DeltaG,1e5,-1e5)` evaluates to `DeltaG` or $\pm 10^5$ if `DeltaG`$\neq \pm\infty$ or `DeltaG`$= \pm\infty$, respectively.

5.3.5 Environmental rates

Some reactions can occur both as part of a cell's metabolism as well as abiotically in the environment. For example, the oxidation of H_2S via reduction of NO_3^- to NO_2^- can be catalyzed by certain microbial clades but can also occur abiotically, albeit very slowly [77]. Similarly, nitrate reduction to ammonium in soils can occur biologically using organic carbon as electron donor as well as abiotically in the presence of sulfate Green rust [29, 28]. In MCM, the `environmental rate` of a reaction can be, for example, a function of substrate concentrations or temperature. Some examples follow below:

```
environmental rate: 0
environmental rate: 1e-5 * exp(-temperature/290) # temperature-dependent
environmental rate: NH4 * O2/(O2 + 1e-8) # linear in NH4 and Michaelis-Menten in O2
environmental rate: NH4 * O2/(O2 + 1e-8) and OU 10 100 # same as previous + correlated noise
```

In general, population growth specifications can comprise any of the following additive components:

- `interpolate`: A piecewise linear interpolation of a given (e.g. measured) time series, e.g.

```
interpolate "data/anammox_rate_measurements.txt"
```

Note that the file path must either be absolute or given relative to the MC model directory.

- An arbitrary custom function of time ('t'), metabolite concentrations, environmental variables, cell concentrations and the reaction's Gibbs free energy (DeltaG, if available), e.g.

```
1/(exp((DeltaG + 96.5*0.12)/(8.3e-3*temperature)) + 1)
```

See section 5.13.2 for general rules on custom functions.

- An Ornstein-Uhlenbeck stochastic process [91, 27] of given standard deviation and autocorrelation time (zero mean), e.g.

```
OU 10 100       # standard deviation 10/L/day, autocorrelation time 100 days
```

- A log-Ornstein-Uhlenbeck stochastic process with given mean, standard deviation and autocorrelation time, e.g.

```
logOU 1 10 100  # mean 1, standard deviation 10/L/day, autocorrelation time 100 days
```

- A periodic component of given amplitude, period and phase offset, e.g.

```
periodic 1e12 1 0  # amplitude 1e12/L/day, period 1 day, phase offset 0
```

At most one of each of the above components is allowed. Components are separated by " and ". Note that in order to account for the effects of environmental reactions on environmental metabolite concentrations, you need to include environmental_production as a component in the metabolite's environmental dynamics (section 5.2.2). See section 2.9 for an introductory example on how to include environmental reactions in a microbial ecosystem model.

5.4 Species

Cell species are defined in the species configuration file, located in the MC model directory. Each species is defined by a unique name (see section 5.13.1 for allowed names), a set of reactions it can perform, a set of available active metabolite uptake and export mechanisms, its dry cell mass and its expected life time under metabolic inactivity. In addition, an environment-dependent modulation of metabolic activity, as well as custom population growth terms, can be defined. Species records thus consist of a species name on a single line, followed by several key:value pairs on consecutive lines (in any order).

- initial concentration (required)
- mass (required)
- life time (required)
- metabolic modulation (optional)
- population growth (optional)
- active uptakes (optional)
- active exports (optional)
- reactions (required)

Any excessive white space, empty lines and comments preceded by the '#' symbol are ignored. The first non-empty line in the file must indicate the file version (1.3). An example species file is given below:

```
file_version: 1.3 # do not remove, move or change this line

Nitrosomonas
   initial concentration: 0
   mass: 3e-13           # cell mass in g dry weight
```

```
   life time: infinite   # cell-death not modeled
   population growth: birth and death and pulse(t-10,1)   # inoculate at day 10
   active uptakes: O2, NH3_NH4, Pi, H_c, H2O, ADP, NAD, CO2
   active exports: NO, N2O, H2O, NH3_NH4, NOH, HNO2, HNO3, H_c, NH2OH, Pi
   reactions: R_biomass_Nitrosomonas, R_maint, R_protein, 5 * amo, R_HAO_noh, R_HAO_no

Nitrobacter
   initial concentration: 1.96e7
   mass: 4e-13
   life time: 100*exp(-(temperature-273)/10)       # temperature-dependent life expectancy
   metabolic modulation: exp((temperature-273)/10) # temperature-dependent metabolic activity
   active uptakes: O2, HNO2, Pi, H_c, H2O, ADP, NAD, CO2
   active exports: NO, N2O, H2O, NH3_NH4, NOH, HNO2, HNO3, H_c, NH2OH, Pi
   reactions: R_biomass_Nitrobacter, R_biomass, R_protein, R_nirBD, 10 nxr
```

click here for source code

The `initial concentration` gives the cell concentration at time 0 of a simulation and `mass` is the cell's mass in g dry weight (needed to convert biomass synthesis to cell production rates). The species' `life time`, `metabolic modulation` and `population growth` are explained in detail in the next section.

Metabolites specified as `active uptakes` or `active exports` are separated by a comma. Uptake/export rate limits for these metabolites are determined during simulation by their `max active uptake/export rates` as specified in the `metabolites` configuration file (see section 5.2.1). For all other metabolites, uptake/export rate limits are determined by their `max passive uptake/export rates`. Active uptake/export versions (if available) are specified using the "." separator, e.g.

```
active uptakes: O2, NH3            # use wild type (if defined) for NH3 uptake
active uptakes: O2, NH3.allele01   # use allele01 for NH3 uptake
```

Reactions available to each species are separated by a comma. Their forward and reverse rate limits are determined during simulation by their `max forward/reverse rates`, as specified in the reactions configuration file (see section 5.3.3). If a reaction has multiple reaction rate limit versions, these are specified using the "." separator, e.g. as

```
reactions: amo            # use wild type (if defined) for amo forward rate
reactions: amo.allele01   # use allele01 for amo forward rate
reactions: amo.allele01.alleleA   # use allele01 for amo forward rate
                                  # and alleleA for amo reverse rate
reactions: amo..alleleA   # use wild type for amo forward rate
                          # and alleleA for amo reverse rate
```

Each reaction can be preceded by an optional gene copy number (GCN), separated from the reaction name by either an asterisk ("`*`") or white space (" "). The default gene copy number is 1. In the examples above, `Nitrosomonas` has 5 copies of the `amo` gene, while `Nitrobacter` has 10 copies of the `nxr` gene. Non-positive GCNs are interpreted as gene absence (and thus reaction unavailability), but GCNs have no effect on metabolic activity otherwise. GCNs are merely used to translate predicted cell concentrations into gene concentrations, for example for comparison with metagenomic data (see section 7). Of course, the very assumption that each reaction corresponds to one gene via one enzyme can be questioned, e.g. when protein complexes are encoded by several genes. Hence, the final interpretation or use of MCM's predictions about gene densities should be case-dependent and subject to the user's judgement.

Note: You should include at least one reaction with non-zero `objective` per species. This will typically be a formal species-specific biomass synthesis reaction [93, 38, 25].

5.4.1 Cell life time

Cell death is modeled as an exponential decay process. The (average) cell `life time` can be `infinite` (no cell death), a constant positive number, possibly modulated by *inhibiting* or *limiting* metabolites, or a `custom` function of time (`t`), metabolite concentrations, environmental variables and cell concentrations

(see section 5.13.2 on custom functions). The syntax is similar to that used for specifying reaction rate limits (see section 5.3.3). Some examples follow below:

```
infinite                            # no cell death
10                                  # die at a rate of 0.1/day
10 inhibited_by ethanol 1.68e-4    # doubled mortality at 1.68e-4 mol/L = 1% alcohol ABV
custom 10 * exp(-light) * 1e-6/(1e-6 + O2)   # increased mortality
                                              # under strong light and high O2
custom 1/Nitrobacter    # mortality increases at higher Nitrobacter concentrations
```

Any expression for a cell's `life time` can optionally be followed by the `no_death_above_production_rate` keyword and a number, which means that mortality only applies when the cell production rate is lower or equal to the given value. For example,

```
life time: 10 no_death_above_production_rate 0.001
```

specifies that mortality shall not apply if the cell production rate (i.e. it's biomass production rate divided by its cell mass) exceeds 0.001 day^{-1}, but should otherwise be 0.1 day^{-1}. The line

```
life time: 10 no_death_above_production_rate 0
```

specifies that cell death shall only occur in the complete absence of metabolism. Practically, this can occur when the cell-metabolic FBA problem is not solvable or if required nutrients are completely depleted.

5.4.2 Metabolic modulation

Apart from from reaction and metabolite transport rate constraints, a general inhibition of cell metabolism depending on environmental conditions can be specified in a species' `metabolic modulation`. The latter is a factor applied to the cell's growth rate, reaction rates and metabolite fluxes post-FBA, i.e. after calculation of the optimal metabolic activity. For example, the following modulation factor specifies that cells become inactive once lactate concentrations exceed the threshold 1 mM:

```
metabolic modulation: custom theta(1e-3 - lactate)
```

The syntax for `metabolic modulation` is almost identical to that of `life time` (section 5.4.1), with the exception that `infinite` is not permissible. In particular, any function of time, metabolite concentrations, environmental variables and cell concentrations can be used. By default, `metabolic modulation` is 1 (i.e. no modulation).

5.4.3 Population growth

By default, cell population growth dynamics comprise cell production and exponential decay ("birth and death"). However, arbitrary dynamics can be specified instead through `population growth`. Some examples follow below:

```
population growth: birth                                     # birth but no death
population growth: birth and death                           # default
population growth: birth and death and -Nitrobacter*amoebae/10 # include predation by amoebae
population growth: birth and death and comb(t,0.1,10)         # inoculate every 10 days
```

In general, population growth specifications can comprise any of the following additive components:

- `birth`: Growth by biomass production.
- `death`: Exponential death at a rate equal to the inverse `life time`.
- `interpolate`: A piecewise linear interpolation of a given (e.g. measured) time series, e.g.

```
interpolate "data/Ecoli_inoculation.txt"
```

Note that the file path must either be absolute or given relative to the MC model directory.

- An arbitrary custom function of time ('t'), metabolite concentrations, environmental variables, cell concentrations, perCapitaBirthRate and perCapitaDeathRate. See section 5.13.2 for general rules on custom functions. For example, population growth for Nitrobacter might be

```
birth and -Nitrobacter * amoebae/10
```

to model grazing by protozoa, or

```
(perCapitaBirthRate - perCapitaDeathRate) * Nitrobacter
```

Technical note: While the 2nd example is equivalent to "birth and death", it is advised to use the latter formulation instead, because certain quantities like biomass recycling or dead cell accumulation can only be calculated in the latter case. Hence, only use the variables perCapitaBirthRate and perCapitaDeathRate if you know what you're doing.

- An Ornstein-Uhlenbeck [91, 27] stochastic process of given standard deviation and autocorrelation time (zero mean), e.g.

```
OU 10 100       # standard deviation 10/L/day, autocorrelation time 100 days
```

- A log-Ornstein-Uhlenbeck stochastic process with given mean, standard deviation and autocorrelation time, e.g.

```
logOU 1 10 100  # mean 1, standard deviation 10/L/day, autocorrelation time 100 days
```

- A periodic component of given amplitude, period and phase offset, e.g.

```
periodic 1e12 1 0  # amplitude 1e12/L/day, period 1 day, phase offset 0
```

At most one of each of the above components is allowed. Components are separated by " and ".

5.4.4 The role of cell mass

MCM measures all reaction and metabolite transport rate limits on a per-cell basis. In contrast, measured metabolite uptake rates are often reported with respect to dry biomass (i.e. without any reference to actual cell counts). The same holds for reaction rate limits given in many cell-metabolic models, such as the ones published by the Model SEED project [32]. This technical difference might seem inhibitory to the parameterization of the model because all per-dry-biomass rates need to be converted to per-cell rates. However, due to the underlying model structure (Eq. (3) and (4)), the model predictions are in fact invariant to the choice of cell mass used to perform the transformation, as long as predicted cell concentrations (if of interest at all) are converted back to dry biomass in retrospect.

5.4.5 Compartmentalization within cells

While not immediately apparent, MCM can accommodate compartmentalized cell-metabolic models of arbitrary complexity. To compartmentalize reactions within cells, one can define formal variants of the same metabolite, corresponding to different compartments, and define formal transport reactions between compartments. For example, to differentiate between protons in the cytosol, the periplasm and the environment, one would define three formal metabolites, H_c, H_p and H_e, that participate in different reactions. Proton pumping from the cytosol to the periplasm might then be represented by a reaction of the form

```
0.5 O2 + 4 H_c + 2 Cyt552e -> H2O + 2 H_p + 2 Cyt552
```

(ETC proton pump by cytochrome aa3 quinol oxidase, oxygen as final electron acceptor [60]).

5.4.6 Species-specialization

Enzymatic capacities and transport efficiencies can differ among microbial strains, even if these share homologous genes. For example, a microbial community might include *E. coli* strains with different terminal oxidases of varying oxygen affinities.

MCM allows the differentiation of reactions, regulatory mechanisms and metabolite transport kinetics between cell species. To specialize reactions (such as biomass production) to individual species, one can define multiple versions of a reaction (using different names) and then selectively make these reactions available to different species. To specify different metabolite exchange or reaction rate limits for different species, there are two options:

Option 1 (recommended): Define multiple alleles (versions) of the enzyme performing the reaction or of the active metabolite transporter. For example, to specify different `O2` uptake rate limits for two *E. coli* strains, define two versions of the active `O2` uptake mechanism, e.g.

```
O2
  max active uptake rate v1: 1e-14
  max active uptake rate v2: 2e-14
```

and then include the appropriate active transporter version (`O2.v1` or `O2.v2`) in each strain (section 5.2.1). A similar approach is possible for reaction rate limits (section 5.3.3).

Option 2: One can define an *intracellular* and *extracellular* (or *environmental*) variant of the metabolite, e.g. `NH3_i` and `NH3_e`, with the conversion between the two limited by species-specific transport reactions. For example, uptake of `NH3_e` might be set to `unlimited`, and `NH3_e` is transformed into `NH3_i` (which is used in the cell's internal reactions) by a reaction whose maximum rate depends on `NH3_e` concentration, i.e.

```
transport_NH3_Nitrosomonas
   description: Nitrosomonas-specific NH3 transport reaction
   equation: NH3_e -> NH3_i
   max forward rate: 1e-10 limited_by NH3_e 26e-6   # Monod-like uptake kinetics
   max reverse rate: unlimited                      # export not explicitly constrained
   objective: 0
```

5.4.7 Enforcing individual reactions

Experimental data might suggest that a particular reaction is activated during growth, contrary to FBA predictions. For example, certain *S. enterica* strains have evolved the capacity to secrete methionine during growth, a trait not captured by standard *S. enterica* FBA models [31]. To enforce the performance of reactions at a rate proportional to biomass production (with fixed proportionality constant), one can include in the reaction a dummy reactant produced exclusively by the biomass synthesis reaction. Maximum uptake/export rates for that dummy metabolite should be set to zero. For example, suppose that

```
X + Y -> Z + W
..
0.1 DNA + 0.2 protein + 0.01 lipids ->
```

are the equations for the enforced and biomass synthesis reactions, respectively. Then modifying these to

```
X + Y + dummy -> Z + W
..
0.1 DNA + 0.2 protein + 0.01 lipids -> 100 dummy
```

will result in 100 flux units through the first reaction per flux unit through the biomass synthesis reaction. To selectively deactivate this coupling in an other species, set the `max active uptake/export rates` for `dummy` to `unlimited` and include `dummy` in the species' `active uptakes/exports` lists.

Alternatively, you can also enforce the performance of a reaction at a constant rate, for example representing minimum maintenance requirements in the form of ATP *leakage*: Simply specify a positive `max forward rate` and set the `max reverse rate` to the negative `max forward rate`, e.g. as follows:

```
ATPM_E_coli
    description:      ATP maintenance requirement
    equation:         ATP + H2O -> ADP + H + Pi
    max forward rate: 5.64e-14
    max reverse rate: -5.64e-14
    objective:        0
```

Of course, the value `5.64e-14` can be replaced by a symbolic parameter or even an arbitrary function of metabolite concentrations and environmental variables. If the resulting FBA problem cannot be solved anymore (e.g. if energy metabolism is too weak to maintain the above reaction in terms of ATP production), MCM will assume zero metabolic activity and thus zero biomass production.

5.5 Observables

5.5.1 Introduction

Within MCM's model framework, at any given point in time the state of a microbial community is fully described by its environmental variables, metabolite concentrations and cell concentrations. However, in practice these so called state space variables (or independent variables) can often not be observed directly, but only inferred from other response or *observable* variables. While observables do not define a system's state per se, they can provide valuable information on the actual state of an ecosystem. For example, the cell concentrations of the ammonia oxidizers *Nitrosomonas* and *Nitrosospira* might not be separately measurable, but the total number of ammonia oxidizing bacteria might be.

To address the conceptual and practical difference between state variables and observations MCM allows the definition of separate observables. Observables can be functions of several state space variables such as metabolite or cell concentrations, or derived variables such as reaction rates and gene concentrations. Observables can even be functions of other observables. While observables do not themselves influence the course of a simulation, they can be used for model fitting (sections 2.6 and 7), sensitivity analysis (section 8) and statistical analysis (section 9).

5.5.2 Defining observables

Observables are are defined in the optional `observables` file, located in the MC model directory. Each observable has a unique name (see section 5.13.1 for allowed names), followed by several key:value pairs given on consecutive lines (in any order):

- `description` (optional)
- `units` (optional): Physical units, for plotting purposes
- `value` (required): Functional definition in terms of other model variables
- `scaling` (optional): Typical scaling (linear or logarithmic), for plotting purposes
- `error model` (optional): Error structure to be used for data comparison

Any excessive white space, empty lines or comments preceded by the '`#`' symbol are ignored. The first non-empty line in the file must indicate the file version (1.3). An observable's `description`, `units` and `scaling` can be empty or omitted, and are only relevant to graphical output style. `error model` can either be omitted, or set to `none`, `normal` (default) or `logNormal`. These choices affect the assumed error structure when comparing the observable's time course to experimental data (e.g. for model fitting, see section 7).

For example, the following `observables` file defines the observable `AOB` as the sum of `Nitrosomonas` and `Nitrosospira` cell concentrations:

```
file_version: 1.3 # do not remove, move or change this line

AOB
```

```
    description: total ammonia oxidizing bacteria
    value:       Nitrosomonas + Nitrosospira
    scaling:     logarithmic
    error model: logNormal
```

Observables can also depend on non-state variables such as reaction rates and gene concentrations. For example, the following observable is defined as the community-wide ammonia oxidation rate multiplied by the reaction's Gibbs free energy and divided by the `amo` gene concentration:

```
amo_energy_flux_per_gene
    value:   rate_cw.amo * DeltaG.amo / concentration.amo
    units:   kJ/(gene*day)  # units only relevant for graphics
    scaling: linear
```

Notice that when specifying an observable's `value`, some variables (e.g. reaction rates or Gibbs free energies) require the addition of a prefix (e.g. "`rate_cw.`" or "`DeltaG.`") to avoid ambiguities. A detailed list of available variables and required prefixes is provided in table 2. For cell-specific reaction rates or cell-specific metabolite export rates the species name is also required, e.g.

```
amo_energy_flux_per_Nitrosomonas_cell
    value:   rate_pc.amo.Nitrosomonas * DeltaG.amo
    units:   kJ/(cell*day)

NO2_exported_per_Nitrosomonas_biomass
    value:   net_export_pc.NO2.Nitrosomonas/cell_mass.Nitrosomonas
    units:   mol/(g*day)
```

Table 2: Model variables that can be included in a variable's `value`, together with required prefixes (section 5.5.2). The abbreviation "cum." stands for cumulative (i.e. time integrated), "env." for environmental (i.e. extracellular), "cw." for community-wide, "pc." for per-cell.

**variable kind**	**prefix**	**example**
time	*none*	`t`
environmental variable	*none*	`temperature`
cell concentration	*none*	`Ecoli`
cell concentration (dead+alive)	`concentration_da.`	`concentration_da.Ecoli`
cell growth rate (per capita)	`growth_rate.`	`growth_rate.Ecoli`
cell birth rate (per capita)	`birth_rate.`	`birth_rate.Ecoli`
cell death rate (per capita)	`death_rate.`	`death_rate.Ecoli`
cell generation	`generation.`	`generation.Ecoli`
cell mass	`cell_mass.`	`cell_mass.Ecoli`
gene concentration	`concentration.`	`concentration.amo`
gene concentration (dead+alive)	`concentration_da.`	`concentration_da.amo`
reaction rate (cw.)	`rate_cw.`	`rate_cw.amo`
reaction rate (pc.)	`rate_pc.`	`rate_pc.amo`
reaction rate (pc., single species)	`rate_pc.`	`rate_pc.amo.Nitrosomonas`
reaction rate (cum., all cells)	`rate_cw_cum.`	`rate_cw_cum.amo`
reaction rate (env.)	`rate_env.`	`rate_env.amo`
reaction rate (cum., env.)	`rate_env_cum.`	`rate_env_cum.amo`
metabolite concentration	*none*	`NO2`
net metabolite export (cw.)	`net_export_cw.`	`net_export_cw.NO2`
pure metabolite export (cw.)	`pure_export_cw.`	`pure_export_cw.NO2`
net metabolite export (cum., cw.)	`net_export_cw_cum.`	`net_export_cw_cum.NO2`
net metabolite export (pc.)	`net_export_pc.`	`net_export_pc.NO2`
net metab. export (pc., single species)	`net_export_pc.`	`net_export_pc.NO2.Nitrosomonas`
net metab. production (env.)	`net_production_env.`	`net_production_env.NO2`
pure metab. production (env.)	`pure_production_env.`	`pure_production_env.NO2`
net metab. production (cum., env.)	`net_production_env_cum.`	`net_production_env_cum.NO2`
reaction Gibbs free energy (ΔG)	`DeltaG.`	`DeltaG.amo`
reaction std. Gibbs free energy (ΔG_0)	`DeltaG0.`	`DeltaG0.amo`

5.6 Focals

Genome-scale metabolic models typically comprise several hundreds of reactions and metabolites [1, 44], rendering the indiscriminate visualization of all dynamical variables impractical. MCM allows the specification of *focal* environmental variables, metabolites, reactions, species and observables of interest, the visualization of which has a higher priority over the rest. Focals are listed by name (one per line) in the optional `focals` file, which should be located in the MC model directory. Each focal environmental variable, metabolite, reaction, species or observable can have an optional information tag (separated from the name by a colon), which is used in MCM visualizations for easier navigation. The structure of this file is exemplified below. Any excessive white space, empty lines or comments preceded by the '#' symbol are ignored. The first non-empty line in the file must indicate the file version (1.3). An example follows below:

```
file_version: 1.3 # do not remove, move or change this line

FOCAL_ENVIRONMENT  # signifies start of focal environmental variables
   pH
   temperature
   salinity

FOCAL_METABOLITES     # signifies start of focal metabolites
   HNO2: nitrite      # this focal metabolite has an optional information tag
   HNO3               # this one does not
   N2O
   NH4
   O2: oxygen

FOCAL_REACTIONS
   amo:   ammonia oxidation to hydroxylamine
   nirBD: nitrite reduction to ammonium

FOCAL_SPECIES
   Nitrosomonas: ammonium oxidizers
   Nitrobacter

FOCAL_OBSERVABLES
   AOB
```

click here for source code

Focal names not corresponding to any defined environmental variable, metabolite, reaction, species or observable are ignored. Each of the five focal lists shown above (`FOCAL_ENVIRONMENTALS`, `FOCAL_METABOLITES`, `FOCAL_REACTIONS`, `FOCAL_SPECIES` and `FOCAL_OBSERVABLES`) is optional and can be empty or completely absent.

Focal names can also include the wildcard symbol "`*`", which represents any possible text (including empty). For example,

```
FOCAL_REACTIONS
   amo: ammonia oxidation to hydroxylamine
   nirBD: nitrite reduction to ammonium
   *
```

sets all reactions to focal, and additionally specifies information tags for the reactions `amo` and `nirBD`. Alternatively,

```
FOCAL_METABOLITES
   *N*
```

specifies all metabolites containing "`N`" as focals.

Technical note: Specifying everything as focal in large models can significantly increase the output size and computation time.

5.7 Symbolic model parameters

MCM allows the use of so called *symbolic* (or *abstract*) parameters, which are represented by ordinary names and can be used in place of numerical constants in the model. The actual value of a symbolic parameter may vary between simulations, for example during sensitivity analysis. Symbolic parameters can replace any numerical constant in the MC model files `environment`, `metabolites`, `reactions`, `species` and `observables` (see sections 5.1, 5.2, 5.3, 5.4 and 5.5), such as reaction rate limits, stoichiometric coefficients, gene copy numbers, cell life expectancies or constants used to define environmental variables. For example, the maximum uptake rate of a metabolite (see section 5.2.1) might be specified as

```
Monod $Vmax_ammonium$ $Khalf_ammonium$
```

instead of

```
Monod 4e-13 2.6e-5
```

The actual value of the symbolic parameters `Vmax_ammonium` and `Khalf_ammonium` can vary depending on case and might even be random. Aside from their practical convenience, symbolic parameters are a prerequisite for higher level model analysis, such as automatic parameter fitting (section 7.3), sensitivity analysis (section 8) and uncertainty analysis (section 9). Furthermore, they provide an easy interface to automated MC model specification (e.g. by 3rd party software).

Symbolic parameters are defined in the optional file `parameters`, located in the MC model directory. Each parameter is characterized by (i) a unique name (see section 5.13.1 for allowed names), (ii) an optional description, (iii) a default numerical value, (iv) numerical lower and upper bounds, (v) an optional probability distribution (used for uncertainty analysis and randomly initialized fitting) and (vi) whether it is fixed, i.e. should always evaluate to its `default` value. Parameter records thus consist of a name on a single line, followed by several key:value pairs given on consecutive lines (in any order):

- `description` (optional)
- `default` (required)
- `minimum` (required)
- `maximum` (required)
- `distribution` (optional)
- `fixed` (required)

Any excessive white space, empty lines and comments preceded by the '#' symbol are ignored. The first non-empty line in the file must indicate the file version (1.3). An example `parameters` file follows below:

```
file_version: 1.3 # do not remove, move or change this line

Khalf_ammonium
   default: 26e-6
   minimum: 1e-8
   maximum: 1e-3
   fixed:   no

Vmax_ammonium
   description: maximum active ammonium/ammonia uptake rate
   default: 1.2e-13  # start from this value when fitting
   minimum: 1e-14    # don't go lower than this during fitting or global sensitivity analysis
   maximum: 1e-11    # don't go higher than this during fitting or global sensitivity analysis
   distribution: uniform # assume uniform distribution for uncertainty analysis
   fixed:   yes      # always use default value
                     # (don't fit and don't vary in sensitivity or uncertainty analysis)
```

click here for source code

The `default` value of a symbolic parameter must be between its `minimum` and `maximum`, and the latter must always be finite. A symbolic parameter's `distribution` can be one of the following:

- Empty (not specified)
- uniform (i.e. uniformly distributed between minimum and maximum),
- logUniform (i.e. log-uniformly distributed between minimum and maximum),
- triangular (i.e. with a triangular probability density between minimum and maximum, maximized at default and zero at minimum and maximum),
- normal, followed by the standard deviation (i.e. parameter follows a truncated-normal distribution between minimum and maximum, with mode at default), e.g.

```
    normal 2e-14
```

- QF, followed by an arbitrary quantile function (i.e. inverse cumulative distribution function) of "p", e.g.

```
    QF -20*log(1-p)        # exponentially distributed with expectation 20
```

  This option allows the specification of an arbitrary probability distribution, as long as its quantile function can be expressed in basic mathematical terms (see section 5.13.2 on valid mathematical functions).

- RG, followed by an arbitrary random generator, i.e. a mathematical expression involving one or more independent random variables, e.g.

```
    RG 20*runiform(1,10)   # uniformly distributed between 20 and 200
```

  See section 5.13.3 on valid random generator expressions.

Symbolic parameters can be used in any of the MC model configuration files environment, metabolites, reactions and species in place of any numerical value, by enclosing the parameter name between two dollar signs. For example, the symbolic parameters Vmax_ammonium and Khalf_ammonium might be used in the specification of ammonium uptake kinetics in the metabolites file:

```
NH4
   description: ammonium
   active uptake objective: 0
   active export objective: 0
   max active uptake rate:  Monod $Vmax_ammonium$ $Khalf_ammonium$
   max active export rate:  unlimited
   max passive uptake rate: 0
   max passive export rate: 0
   environmental dynamics:  initial 0.916e-3 flux microbial_export
```

5.8 Macros

MCM allows the definition of text macros in MC models, i.e. fragments of code represented by a short name. For example, consider an MC model comprises several cell species with the same life time expression (section 5.4.1), e.g.

```
Ecoli_M23
   life time: 10 limited_by O2 1e-9 inhibited_by ethanol 1e-5 inhibited_by light 30
   ...

Ecoli_K12
   life time: 10 limited_by O2 1e-9 inhibited_by ethanol 1e-5 inhibited_by light 30
   ...

Acetobacter
   life time: 10 limited_by O2 1e-9 inhibited_by ethanol 1e-5 inhibited_by light 30
   ...
```

Then this can be simplified to

```
Ecoli_M23
   life time: $generic_life_time$
   ...

Ecoli_K12
   life time: $generic_life_time$
   ...

Acetobacter
   life time: $generic_life_time$
   ...
```

with the macro `generic_life_time` defined as

```
generic_lifetime: 10 limited_by O2 1e-9 inhibited_by ethanol 1e-5 inhibited_by light 30
```

Note how the colon ':' separates the macro name from its value, and that macros are referred to by the name enclosed in '$' signs. Macro values can contain any text sequence except for line breaks, and can even contain references to symbolic parameters (which are evaluated after macro replacement). Macros can be referred to in the any of the MC model files (`environment`, `metabolites`, `reactions`, `species` or `observables`).

Macros can be defined in an optional `macros` file (one macro per line), located in the MC model directory. Any excessive white space, empty lines and comments preceded by the '#' symbol are ignored. The first non-empty line in the file must indicate the file version (1.3). Macro names must meet similar criteria to other MC objects, as explained in section 5.13.1. An example `macros` file is given below:

```
file_version: 1.3 # do not remove, move or change this line

# generic life time for Ecoli and acetobacter
generic_lifetime: 10 limited_by O2 1e-9 inhibited_by ethanol 1e-5 inhibited_by light

# pH inhibition for non-acidophilus
# Adopted from [Presser et al 1997, E. coli M23]
inhibition_by_pH: custom max(0, 1-10^($pHmin$ - pH))
```

click here for source code

Macros can also be defined in the MCM command line (section 4.3). Command line macros overwrite any homonymous MC model macros.

5.9 Model templates

MC models containing multiple variants of a cell species, environmental variables etc, can be constructed using so called *templates*. A template consists of a template *body*, which defines a text block to be replicated, and a set of template variables the range of which determines the variation of the template body across replicates. For example, the template

```
template{V:1-10}{
  Nitrosomonas_$V$
    initial concentration: 1e7
    mass:             $V$e-12
    life time:        infinite
    reactions:        amo
    active uptakes:   O2, NH4
}
```

has one template variable (V) ranging from 1 to 10, and a template body defining a *Nitrosomonas* cell variant for each value of V. Both the cell name and cell mass depend on the value of V, which is represented in the template body by V. The template thus defines 10 *Nitrosomonas* variants with names Nitrosomonas_1, .., Nitrosomonas_10 and cell masses 1 pg, .., 10 pg, respectively.

Similarly, the following template defines three metabolites (NH4, O2 and NO3) with similar properties:

```
template{name:NH4,O2,NO3}{
   $name$
      max active uptake rate:  1e-14
      max passive uptake rate: unlimited
      max passive export rate: unlimited
      environmental dynamics:  initial 0 flux microbial_export
}
```

As the above examples show, a template variable's range can either be an integer interval (e.g. 1-10) or a comma-separated list of text values (e.g. NH4, O2 and NO3). To include arbitrary text in a variable's value[8], enclose it in single or double quotes.

Any arbitrary text block can be templated in an MC model[9]. Text not included in a template is kept as-is. Template variables follow certain naming rules (explained in section 5.13.1) and are evaluated before symbolic parameters (section 5.7) and macros (section 5.8).

A template may specify multiple template variables (separated by ";"), in which case each possible combination of their values defines a separate replicate of the template body. For example, the following block defines a metabolite with 15 versions of active uptake rate limits (section 5.2.1):

```
NO2
    template{A:1-5;B:1-3}{max active uptake rate: Monod $Vmax_NO2_$V$$ $Khalf_NO2_$B$$}
    max passive uptake rate: 0
    max passive export rate: 0
    environmental dynamics:  initial 0 flux microbial_export
```

In the above case, Vmax_NO2_1,..,Vmax_NO2_5 and Khalf_NO2_1,..,Khalf_NO2_3 need to be defined as symbolic parameters or macros (since after evaluation of the template variable V, they are still enclosed in a pair of '$' signs).

MC model files containing templates must have the name suffix _template and be located within the MC model directory. These files and are not themselves valid MC model files, and are generally ignored by MCM. Instead, they are used to generate MC models using the MCM command expandMCmodelTemplates. For example, an MC model directory containing the files

```
metabolites
species
observables
reactions_template
environment_template
```

will, following execution of expandMCmodelTemplates, contain the files

```
metabolites
species
observables
reactions_template
environment_template
reactions
environment
```

The files reactions and environment have been generated using the corresponding template files, and together with the pre-existing metabolites, species and observables files, define a complete MC model.

[8]Except for curly brackets "".
[9]With the exception that the templated text block may not contain curly brackets "{}".

Technical note: MC model files generated by expandMCmodelTemplates can be manually edited afterwards. Re-executing expandMCmodelTemplates will not replace them. Alternatively, calling the variant expandMCmodelTemplatesReplace replaces existing model files without warning.

5.10 Periodic dilutions

MCM allows the specification of periodic dilutions of microbial communities, e.g. to simulate long-term experiments involving batch culture growth with periodic dilutions into identical media. Dilutions are performed at regular time intervals and result in a reduction of cell concentration by a fixed ratio. Metabolites whose environmental concentration is a dynamic variable, i.e. specified via a rate of change (see options iii and iv in section 5.2.2), can also optionally be diluted at the same ratio. Diluted metabolites are diluted into ambient medium in which their concentration is their initial concentration at time 0. Hence, a dilution factor of 1/10 will reduce all cell concentrations by a factor of 10 and set dynamical metabolite concentrations to $0.1 \times C + 0.9 \times C_\text{i}$ (where C and C_i are the current and initial metabolite concentrations, respectively). Dynamic environmental variables, i.e. specified via a rate of change (see options iii and iv in section 5.1.3), can be diluted in a similar way.

Dilutions can be specified using the following commands, preceding any simulation:

```
set dilute                    # enable periodic dilutions
set dilutionPeriod 1          # dilute every day
set dilutionFactor 0.01       # dilute by a factor 1/100

# also dilute dynamic environmental variables
set includeEnvironmentalsWhenDiluting

# also dilute metabolites with dynamic concentrations
set includeMetabolitesWhenDiluting

# alternatively, to not dilute any metabolites, use
unset includeMetabolitesWhenDiluting
```

click here for source code

5.11 Compartmentalized ecosystem models

MCM can accommodate microbial ecosystem models consisting of multiple compartments, each of which is characterized by different cell and metabolite concentrations as well as environmental variables. For example, a simplified lake ecosystem model could consist of three compartments corresponding to the oxic, suboxic and anoxic zones (Fig. 17). Compartments can interact through inter-compartmental fluxes of cells and metabolites, such as oxygen diffusing from the oxic layer to the suboxic layer or cells sinking from each layer to the layer below.

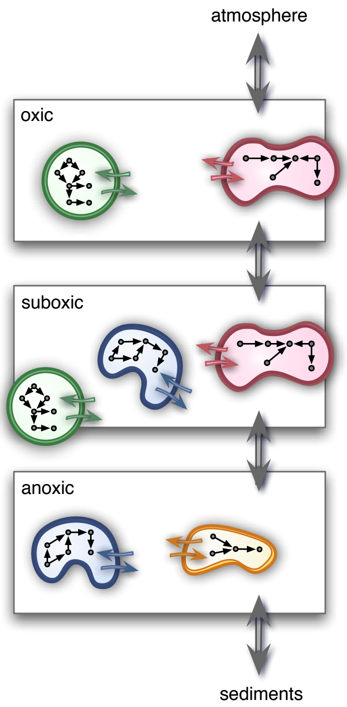

Figure 17: Illustration of a lake ecosystem model with 3 compartments corresponding to the oxic, suboxic and anoxic layer. Each compartment is a non-perfectly closed microbial ecosystem with dynamic cell and metabolite concentrations. Grey arrows represent metabolic and cell fluxes between the compartments, atmosphere and sediments.

To construct a compartmentalized ecosystem model, each metabolite and cell species must be defined separately for each compartment in which they are present. In the above example, each metabolite would have up to three variants corresponding to the oxic, suboxic and anoxic layer. Environmental variables (such as temperature) that differ between compartments must also be defined separately for each compartment. Fluxes between compartments are then specified as environmental metabolite fluxes (section 5.2.2) or additional terms in cell population growth (section 5.4.3). For example, the following `metabolites` file defines oxygen for each of the three compartments, subject to exchange with the atmosphere as well as exchange between compartments:

```
file_version: 1.3 # do not remove, move or change this line

O2_ox
   max active uptake rate:  Monod 1e-14 1e-6
   max passive uptake rate: 0
   max passive export rate: unlimited
   environmental dynamics:  initial 2.2e-4 flux microbial_export and (2.2e-4 - O2_ox)*100

O2_sub
   max active uptake rate:  Monod 1e-14 1e-6
   max passive uptake rate: 0
   max passive export rate: unlimited
   environmental dynamics:  initial 2.2e-4 flux microbial_export and (O2_ox - O2_sub)*0.1

O2_an
   max active uptake rate:  Monod 1e-14 1e-6
   max passive uptake rate: 0
   max passive export rate: unlimited
   environmental dynamics:  initial 2.2e-4 flux microbial_export and (O2_sub - O2_an)*0.1
```

Reactions can either (i) be the same for all compartments, or (ii) be specified separately for each compartment. In case (i), cells will need to include reactions that formally convert between compartment-specific metabolites and the metabolite names used in the reactions. In case (ii), each cell species variant will

need to include the appropriate reaction variants. This approach has the benefit that community-wide reaction rates are predicted separately for each compartment. For example, aerobic glucose catabolism may be defined by the reactions

```
glc_catabolism_ox
   equation:         glucose_ox + 6 O2_ox -> 6 CO2 + 6 H2O
   max forward rate: unlimited
   max reverse rate: 0

glc_catabolism_sub
   equation:         glucose_sub + 6 O2_sub -> 6 CO2 + 6 H2O
   max forward rate: unlimited
   max reverse rate: 0

glc_catabolism_an
   equation:         glucose_an + 6 O2_an -> 6 CO2 + 6 H2O
   max forward rate: unlimited
   max reverse rate: 0
```

Accordingly, the following `species` file specifies that the species *Nitrobacter* catabolizes glucose and sinks from the oxic to the suboxic layer at a per-capita rate of 0.1/day:

```
file_version: 1.3 # do not remove, move or change this line

Nitrobacter_ox
   initial concentration: 1e7
   population growth:     birth and death and -0.1*Nitrobacter_ox
   mass:                  2.8e-13
   life time:             20
   reactions:             glc_catabolism_ox
   active uptakes:        O2_ox, glucose_ox

Nitrobacter_sub
   initial concentration: 0
   population growth:     birth and death and 0.1*Nitrobacter_ox
   mass:                  2.8e-13
   life time:             20
   reactions:             glc_catabolism_sub
   active uptakes:        O2_sub, glucose_sub
```

The construction of compartmentalized MC models can be significantly streamlined using model templates (see section 5.9). For example, the above reactions may be defined through the template

```
template{C:ox,sub,an}{
   glc_catabolism_$C$
      equation:         glucose_$C$ + 6 O2_$C$ -> 6 CO2 + 6 H2O
      max forward rate: unlimited
      max reverse rate: 0
}
```

5.12 Non-stationary cell models

Conventional FBA assumes internally quasi-stationary cells, i.e. in which all fluxes are stoichiometrically balanced by metabolite uptake/export rates, and whose metabolic activity only depends on the external environment but not on intracellular dynamics. This assumption is usually justified by the general observation that intracellular metabolic transients are much shorter than the time scales associated with cell growth [51, 14].

However, in certain cases it may be desirable to extend conventional FBA models to include intracellular dynamical state variables, for example to account for delays in enzyme synthesis during response to

external perturbations [14, 4]. Non-stationary cell models also enable so called regulatory FBA (rFBA) approaches [14, 13, 33], in which dynamical regulatory constraints restrict the FBA solution space depending on the abundance of internally produced inhibitors or activators.

MCM allows the incorporation of cell-internal dynamical variables whose rate of change can depend, for example, on the cell's current metabolic activity or external factors such as temperature and metabolite concentrations. In turn, cell-internal variables can, for example, influence a cell's metabolism or death rate. Dynamical cell internal variables are formally defined as environmental variables (section 5.1), as illustrated in the examples below.

Example 1: Let us consider a hypothetical enzyme produced at a rate proportional to the rate of reaction `amo` in species `Nitrosomonas`. The environmental variable representing the cell-internal abundance of that enzyme could be similar to the following:

```
amo_enzyme_Ns
   dynamics: initial 0 rate 0.1 * rate_pc.amo.Nitrosomonas - amo_enzyme_Ns/5
   units:    mol/cell
   sign:     positive
```

Observe that we have included an exponential decay rate (0.2/day), so that in the absence of `amo` activity `amo_enzyme_Ns` gradually drops to zero. We also specify the `sign` as `positive` to make sure `amo_enzyme_Ns` never becomes negative, e.g. due to numerical errors. Just like any other environmental variable, `amo_enzyme_Ns` could influence microbial metabolism or population dynamics. For example, if `amo_enzyme_Ns` accelerates `Nitrosomonas` cell death, then one might specify the `Nitrosomas` `life time` as follows:

```
Nitrosomonas
   life time: 10*exp(-amo_enzyme_Ns)
   ...
```

Residual enzymes can also contribute to post-transcriptional or post-translational regulation [4]. For example, if `amo_enzyme_Ns` acts as an inhibitor to the reaction `nir`, then one might specify a `Nitrosomonas`-specific version of `nir` whose maximum forward rate decreases with increasing `amo_enzyme_Ns`:

```
nir
   max forward rate:    1e-14                           # default rate limit
   max forward rate Ns: 1e-14/(1e-10 + amo_enzyme_Ns)   # rate limit in Nitrosomonas
   max reverse rate:    0
   ...
```

Care should be taken to assign the appropriate `nir` version to the appropriate cell species (i.e. `nir.Ns` only to `Nitrosomonas`; see section 5.4). See section 5.3.3 for details on defining reactions.

Example 2: Let us consider a hypothetical freeze-shock resistance protein that is produced by Nitrosomonas during exposure to low temperatures at a small cost (in terms of ATP). We shall assume that in the absence of this protein, a cell's metabolism is reduced at temperatures below 10°C. The appropriate environmental variable might look as follows:

```
freeze_protein_Ns
   dynamics: initial 0 rate max(0, 10-temperature)*1e-15 - freeze_protein_Ns/5
   units:    mol/(cell*K)
   sign:     positive
```

Observe that `freeze_protein_Ns` is only produced at temperatures below 10°C, and at a rate proportional to the difference 10-`temperature`. Furthermore, `freeze_protein_Ns` decays at an exponential rate (0.2/day) following production. Also note that the environmental variable `temperature` needs to be defined separately (in this case in °C; see section 5.1.4 for details and section 2.7 for an example).

To account for `freeze_protein_Ns` production costs, we specify an additional reaction (section 5.3) that consumes ATP at a rate proportional to the protein's production rate:

```
freeze_protein_production_cost_Ns
   equation: 15*ATP + 15*H2O -> 15*ADP + 15*H + 15*Pi
   max forward rate: +max(0, 10-temperature)*1e-15
   max reverse rate: -max(0, 10-temperature)*1e-15
   ...
```

Observe that by choice of the reactions rate limits, we are enforcing the performance of this reaction at a rate equal to the rate of freeze_protein_Ns production. We assumed that 15 mole ATP are needed to produce one mole of freeze_protein_Ns. To model the opposite effects of temperature and freeze_protein_Ns on cell metabolism, we can adjust the metabolic modulation of Nitrosomonas cells, e.g. as follows:

```
Nitrosomonas
   metabolic modulation: exp(-max(0,10-temperature) * 1e-15/(1e-15+freeze_protein_Ns))
   ...
```

Observe that in the absence of freeze_protein_Ns, a cell's metabolic activity drops exponentially with every °C below 10°C, however the strength of that effect is weakened by freeze_protein_Ns. Alternatively, freeze_protein_Ns could also influence specific reactions, e.g. by modifying their rate limits (section 5.3.3).

5.13 General notes

5.13.1 Naming rules

Environmental variables, metabolite, reaction, species, observable, symbolic parameter and macro names must only contain letters (a–z, A–Z), digits (0-9) and/or underscores (_), must not begin with a digit and must not be "and" or "end". Environmental variable, metabolite, reaction, species and observables names must not clash and cannot be "t" (which is reserved for time) nor "DeltaG". Metabolites, reactions, species and observables must also not be named "pH" nor "temperature" (which are reserved for environmental variables).

Active metabolite transport and reaction limit version names (sections 5.2.1 and 5.3.3) must only contain letters (a–z, A–Z), digits (0-9) and/or underscores (_). Template variable names (section 5.9) must only contain letters (a–z, A–Z), digits (0-9) and/or underscores (_), and must not begin with a number.

All names are case-sensitive (i.e. NH3 is not the same as nh3).

5.13.2 Custom functions

Many biochemical and physiological specifications (e.g. maximum metabolite uptake rates, maximum reaction rates) can include custom analytic functions of time, environmental variables or metabolite concentrations (e.g. see sections 5.2.1, 5.2.2, 5.3.3 and 5.4.1). The general syntax for analytic functions follows standard mathematical conventions, as known for example from C-like programming languages or MATLAB [50]. Only round bracket types ("()") are allowed. Variables and function names are separated by spaces and/or binary operators (implicit multiplication is not supported). Recognized standard function names are sin, cos, tan, cot, acos, asin, atan, acot, cosh, sinh, tanh, coth, exp, log, log10, sqrt, abs, min, max, atan2, pulse, comb, theta, ceil and floor. The functions ceil and floor return the next-largest and next-lowest integer to their argument, respectively. theta(x) returns +1 for positive x and 0 otherwise. The function atan2 takes 2 variables and is similar to the homonymous C++ function. pulse(x, y) evaluates to 1 if $0 \leq x \leq y$ and 0 otherwise, while comb(x, w, p) evaluates to 1 if $0 \leq (x \mod w) \leq p$ and 0 otherwise. min(x, y) and max(x, y) return the minimum and maximum of x and y, respectively. Supported binary operators are +, -, *, /, % (modulo) and ^ (exponentiation). The constant pi (= 3.141592..) is also recognized. Example expressions follow below:

```
NH3^3/(NH3^3 + 1e-6^3)            # valid. Monod-like function of NH3 with Hill coefficient 3
0.1 * theta(NH3 - 1e-6)           # valid. Will be 0.1 for NH3>1e-6, and 0 otherwise
NH3 * [1 + sin(t/10) * exp(pH+10)] # valid. A function of NH3, t, and pH
NH3[1 + sin(t/10) * exp(pH+10)]    # invalid. NH3 is erroneously interpreted as a function
```

```
max(O2, CO2, H2)                  # invalid. Use max(O2, max(CO2, H2)) instead
```

In addition to standard mathematical functions, several functions associated with acid dissociation and pH are also supported. A detailed list is given in Table 3. These functions can be used, for example, to define the environmental variable pH (section 5.1) as a function of various acid concentrations in water:

```
pH:
  dynamics: value twoAcids2pH(lactic_acid, acetic_acid, 1.38e-4, 1.753e-5, 293.15)
```

The function escapeNAN(x, y) can be used to define an alternative expression (y) in case the first expression (x) evaluates to NaN. For example the function

```
escapeNAN(NH3/O2, 1)
```

will evaluate to 1 if both NH3 and O2 are zero. More generally, the function escapeNAN2(x, y_1, y_2) can be used to choose between y_1 (if x is not NaN) or y_2 (if x is NaN). Similarly, the function escapeInf(x, y) can be used to define an alternative expression (y) in case x evaluates to $\pm\infty$. More generally, the function escapeInf2(x, y, z) evaluates to x, y or z if $x \neq \pm\infty$, $x = +\infty$ or $x = -\infty$, respectively.

Table 3: List of functions for calculating pH (section 5.13.2). All functions apply to aqueous solutions of monoprotonic acids at temperature T (in Kelvin). Acid dissociation constants must be given in mol/L for standard temperature (25°C).

name	parameters	description
oneAcid2pH	acid name & K_a, T	pH of one acid with dissociation constant K_a
twoAcids2pH	acid names & K_a^1, K_a^2, T	pH of two acids with dissociation constants K_a^1, K_a^2
threeAcids2pH	acid names & $K_a^1, .., K_a^3$, T	pH of three acids with given dissociation constants
fourAcids2pH	acid names & $K_a^1, .., K_a^4$, T	pH of four acids with given dissociation constants
fiveAcids2pH	acid names & $K_a^1, .., K_a^5$, T	pH of five acids with given dissociation constants

5.13.3 Random generators

Random generators (e.g. for the random distribution of symbolic parameters, section 5.7) follow a similar syntax to custom functions (section 5.13.2), but can additionally contain one or more independent random variables, e.g.

```
rchisquared(2)                   # chi-squared with 2 degrees of freedom
rnormal(1,0)^2 + rnormal(1,0)^2  # also chi-squared with 2 degrees of freedom
asin(runiform(-1,1))             # arcsin of a random value picked uniformly within [-1,1]
```

Table 4 lists all available random variables.

Table 4: Overview of available random variables for the construction of random generators (section 5.13.3).

name	params.	description
rnormal	2	normal with given mean and standard deviation
rchisquared	1	χ^2 with given degrees of freedom
runiform	2	uniform within the given interval
rpoisson	1	Poisson with given mean
rbernoulli	1	Bernoulli trial with given probability of success
rbinomial	2	binomial with given number of trials and probability of success
rloguniform	2	log-uniform within the given interval
rcauchy	2	Cauchy with given median and scale parameter (γ)
rtriangular	3	triangular with given mode, minimum and maximum

5.13.4 Physical units

MCM expects certain quantities in specific physical units, summarized in table 5 below.

Table 5: Overview of physical units used by MCM.

quantity	unit
time	days
cell dry mass	g
cell and gene concentration	1/L
cell growth rates	1/day
frequency	1/day
metabolite concentration	mol/L
metabolite fluxes (uptake/export) per cell	mol/(cell · day)
community-wide and environmental metabolite fluxes	mol/(L · day)
metabolite release via biomass recycling	mol/(g dry biomass)
reaction rate per cell	mol/(cell · day)
community-wide reaction rates	mol/(L · day)
acid dissociation constants	mol/L
objective coefficients of reactions	(g dry biomass)/(mol flux)
objective coefficients of metabolite transports	(g dry biomass)/(mol transported)
Gibbs free energy of formation of a metabolite	kJ/mol
Gibbs free energy of a reaction	kJ/mol
Gibbs free energy rate (community-wide)	kJ/(L · day)
temperature	°C, °K or °F

An overview of physical units can also be obtained with the MCM command `modelSyntax`. In general, environmental variables can have arbitrary units and their interpretation is the responsibility of the model designer. In fact, MCM has no way of checking whether an environmental variable makes any physical sense. The sole exception is `pH`, which is interpreted as $-\log_{10}[H^+]$. Furthermore, for some models `temperature` can only be defined in one of the units °K, °C or °F (an error message will indicate so otherwise).

Technical note: By convention, MCM considers microbial communities in a 3D setting, so that cell concentrations and metabolite concentrations are measured with respect to 3D volume units (L). However, the model can also be adapted to other dimensions (e.g. two), by formally reinterpreting the volume unit L accordingly (e.g. as surface area). Of course, then, all volume-specific model parameters (e.g. metabolite half-saturation concentrations, environmental metabolite fluxes, acid dissociation constants, pH) will also need to be adapted to the new volume unit.

5.14 `micog`: Converting conventional FBA models into MC models

Together with MCM, you should have obtained a `python` script called `micog.py` (**mi**crobial **co**mmunity **g**enerator). This script converts conventional FBA models in SBML file format[10] [37], such as generated by the Model SEED pipeline [32], into a draft MC model that can be used with MCM. Multiple input SBML files can be specified simultaneously using conventional shell wildcards (in quotes); all metabolites and reactions are pooled together and cell metabolic potentials are defined according to where the different metabolites and reactions were found[11]. Metabolite uptake/export mechanisms are detected through reactions with either no reactants or no products[12]; for the involved metabolites the cell is assumed to have active uptake or export mechanisms, limited according to the original reaction rate limits. Note that `micog` will correct all non-MCM compliant names (in SBML referred to as "IDs") by replacing invalid characters with and inserting underscores ("_") where necessary. `micog` will print out several reports documenting the conversion process. The generated draft MC model typically requires further manual curation, for example to adjust uptake/export rate limits. A detailed description of `micog` options can be obtained by calling

```
./micog.py --help
```

[10] `micog` currently supports SBML Level 2 version 1, and has been tested on `python` 2.7. `micog` requires the `libsbml` module.
[11] This assumes that metabolite and reaction IDs are consistent across SBML files.
[12] Non-flux balanced metabolites, i.e. for which SBML specifies `boundaryCondition=true`, are omitted from the model.

in the terminal. Some usage examples follow below.

Note: You might want to install `micog` to `/usr/local/bin/` (or similar) for system wide access. It might also be necessary to give `micog` execute permissions before running, e.g. using

```
chmod u+x micog.py
```

in the terminal.

Technical note: There exists no standardized way to save cell-metabolic FBA models in SBML format (despite its widespread use), so you might need to modify the `micog` script to convert your favorite files. `micog` has been successfully used to convert SBML files published by the Systems Biology Research Group [70, 24], the BiGG metabolic reconstruction database [79] and files generated by the Model SEED pipeline [32].

5.14.1 Example 1

In this example we will be converting a cell metabolic model of *M. tuberculosis*, obtained from the BiGG database [79]. Let us download the `compartmentalized M. tuberculosis iNJ661` model in SBML format from the BiGG wegpage. You should obtain the file `SBML_export.xml`. We can now convert the file to a draft MC model using the command:

```
micog.py -i SBML_export.xml \
         -o MC_model_tuperc \
         --species_naming name_tag \
         --cell_concentration 1e3 \
         --life_time "infinite" \
         --cell_mass 1e-13 \
         --objective original \
         --environmental_metabolite_dynamics "concentration 0" \
         --verbose
```

click here for source code

("\" denotes a line continuation). The first two options (`-i` and `-o`) define the input SBML file(s) and output directory, respectively. The option `--species_naming` defines how names are assigned to cell species in the MC model: In our case, the choice `name_tag` specifies that `micog` should use the model '`name`' tag in the SBML file (which in our case is "`M. tuberculosis iNJ661`", and which will be converted to the MCM-compatible name "`M__tuberculosis_iNJ661`"). We arbitrarily set the initial cell concentration to 1×10^3 cells/L. The cell life time is set to `infinite` (i.e. no cell death), although this can be changed in the draft MC model later on.

Since the `iNJ661` model defines rates with respect to dry biomass, we need to provide `micog` with the dry cell mass (here arbitrarily chosen to be 1×10^{-13} g) in order to convert these to per-cell rates (as required by MCM). The model already defines an objective function in terms of biomass synthesis (g dry weight per mmol reaction flux), which is why we chose `--objective original`.

Technical note: The alternative choice to `original` is `biomass`, in which case `micog` tries to detect a *biomass* metabolite (whose name is specified using the `--biomass_name` option) and a biomass synthesis function, and sets the objective to the production of the biomass metabolite. This is more suitable for SBML files generated using the Model SEED pipeline [32] (see the example in section 5.14.2).

We set the concentration of all metabolites to zero using the `--environmental_metabolite_dynamics` option. However, this will not affect the cell's metabolism, because the iNJ661 model specifies all metabolite uptake/export constraints as constants regardless of substrate concentrations. It is then our responsibility to change these to substrate-dependent functions. Furthermore, individual metabolite dynamics can of course be adjusted in the draft MC model. The `--verbose` option tells `micog` to be *verbose*, i.e. print out detailed information along the way.

The generated draft MC model (comprising 937 reactions, 826 metabolites and 1 species) will be written to the `MC_model_tuperc` directory. Simulating the model in MCM without any further adjustments (see section 6) predicts a per-capita growth rate of 1.285 day^{-1}.

5.14.2 Example 2

In this example, we shall convert two cell-metabolic models for *Nitrobacter winogradskyi* Nb-255 and *Nitrosomonas europaea* obtained as SBML files from the Model SEED pipeline [32] (models `Seed323098.3` and `Seed228410.1`, respectively).

Recall that the cell mass will influence the conversion from mass-specific constraints in the original models to cell-specific constraints in the MC model. Since the model will include different cell species, you should specify their mass individually for each species. To do so, rename the two downloaded SBML files, `Seed323098.3.xml` and `Seed228410.1.xml`, to

```
Nitrobacter_mass=4e-13.xml
Nitrosomonas_mass=3e-13.xml
```

respectively. As a result, `micog` will infer cell masses from file names. Place the two SBML files into a new directory (let's call it `nitrifier_sbmls`).

Converting the two models into an MC model using `micog` is straightforward:

```
micog.py -i "nitrifier_sbmls/*.xml" \
         -o MC_model_nitrifiers \
         --species_naming file_name \
         --life_time "infinite" \
         --cell_concentration 1e3 \
         --environmental_metabolite_dynamics "initial 0 flux microbial_export" \
         --objective biomass \
         --biomass_name Biomass_b \
         --verbose
```

click here for source code

Note the necessary quotes around the input file path and the wildcard "`*`" representing *any* file name. Via the `--species_naming` option we tell `micog` that species names are to be inferred from the SBML file names (i.e. `Nitrobacter` and `Nitrosomonas`). We set `--life_time` to `infinite` since we're not interested in modeling cell death and we set the initial cell concentration for both species arbitrarily to 1×10^3 cells/L. In contrast to the previous example, we use the `--environmental_metabolite_dynamics` option to specify all metabolites as dynamical with an initial concentration of zero and a rate of change determined by the net microbial export (see section 5.2.2 for other options).

In contrast to the previous examples, `--objective` is now set to `biomass`, which means that `micog` will try to identify a *biomass* metabolite and define the objective function according to which reactions produce that metabolite. The name by which the latter is identified, is specified using the `--biomass_name` option (in our case "`Biomass_b`", which is used by all SEED models). This is the appropriate choice for Model SEED FBA models, because the latter do not define an objective function per-se.

The generated draft MC model will comprise 1001 metabolites, 1009 reactions and 2 species. You will likely see a long list of warnings, telling you that reaction rate limits are inconsistent between `Nitrobacter` and `Nitrosomonas` (e.g. `9.6e-12` vs `7.2e-12`). This is because we used different cell masses to convert from mass-specific rate limits to cell-specific rate limits, while the two original models define the same (arbitrary) rate limits for all reactions. Note that this (common) practice is advised against for MC models, i.e. unconstrained reaction rates should be set to `unlimited` rather than just a *large* number representing infinity. You should therefore change all `max forward rates` and `max reverse rates` of values `9.6e-12` or `7.2e-12` to `unlimited` in the draft MC model.

Simulating the resulting draft MC model using MCM (see section 6) predicts growth rates of 1412 day^{-1} for `Nitrobacter` and 4976 day^{-1} for `Nitrosomonas` (or 1003 day^{-1} and 1990 day^{-1} for `Nitrobacter` and `Nitrosomonas`, respectively, if reaction rate limits are left unchanged). Obviously these growth rates are unrealistically high, and result from mostly arbitrary reaction rate and metabolite exchange rate limits set by the Model SEED pipeline. It is the responsibility of the MC model designer to curate the resulting draft model and introduce physiologically realistic limits.

6 Running a single MCM simulation

Single simulations of a microbial community model can be performed using the `runMCM` command. Running a simulation requires the specification of an MC model in terms of `environment`, `metabolites`, `reactions`, `species` and `observables` files as described in section 5.

MCM will generate predictions of the time course of several output variables at the community level, such as reaction rates (total as well as average per cell), metabolite concentrations in the environment, cell concentrations (alive or dead+alive), gene concentrations (corresponding to cell concentrations) and metabolite fluxes (total as well as average per cell). If the control variable `output_timeSeriesAnalysis` is set to `save` (`saveAndPlot`), MCM also saves (and plots) all periodograms and autocovariances for all time courses [61, 76]. In addition, MCM can plot the time course of several biodiversity measures and rank-abundance relationships for cells as well as genes (depending on the control variables `output_cellBiodiversity` and `output_geneBiodiversity`). The predicted time courses can optionally be statistically evaluated against available time series data, which can be provided in the form of simple tabular text files (see section 7).

At the beginning of or during a simulation, MCM can optionally produce several additional outputs:

- If the control variable `output_speciesSpecificMetabolism` is set to `save` (`saveAndPlot`), MCM will also save (and plot) the time courses of reaction rates and metabolite fluxes for each individual species[13].
- If the flag `plotMetabolicPotentialHeatmap` is set, MCM will generate heatmaps of the metabolic potential of each species. If the flags
  - `metabolicHeatmaps_onlyFocalMetabolites`,
  - `metabolicHeatmaps_onlyFocalReactions` and/or
  - `metabolicHeatmaps_onlyFocalSpecies`

  are set, only focal metabolites, reactions and/or species are included, respectively.
- MCM can also generate heatmaps of the current metabolic activity of each species at arbitrary times specified via the control variable `plotMetabolicActivityHeatmaps`, e.g. as

```
# plot metabolic activity heatmaps at times 5, 15, 25, ..
set plotMetabolicActivityHeatmaps start 5 step 10

# plot metabolic activity heatmaps at times 80, 90, 100
set plotMetabolicActivityHeatmaps start 100 step -10 end 80

# plot metabolic activity heatmaps at times 5 and 15
set plotMetabolicActivityHeatmaps at 5 15

# plot metabolic activity heatmaps at times 5 and 15, as well as 50, 60, 70, ..
set plotMetabolicActivityHeatmaps at 5 15 and start 50 step 10

# don't plot any metabolic activity heatmaps
set plotMetabolicActivityHeatmaps never
```

  Similarly, MCM can also generate heatmaps of the cumulative metabolic activity per species, i.e. integrated over time and all cells, or of the average metabolic activity per cell, i.e. averaged over time. The times at which these heatmaps are generated are defined using the control variables `plotCumulativeMetabolicActivityHeatmaps` and `plotAverageMetabolicActivityHeatmaps`, respectively. See Figures 27 and 28 for example metabolic potential and activity heatmaps. See section 14.3.4 on modifying the appearance of heatmaps. Together with metabolic activity heatmaps, MCM will also write detailed reports to the files

  - `metabolic_activity.txt`,

[13] Note that for large models this might use up a lot of disk space. All reaction rates and metabolite fluxes are saved, but only focal reactions and metabolites are plotted.

  - cumulative_metabolic_activity.txt, or
  - average_metabolic_activity.txt.

- MCM can also generate chord diagrams of metabolic fluxes across reactions (or cell species). These so called metabolic flux diagrams (or microbial flux diagrams) can be viewed and interacted with in any modern web browser. See Fig. 29 for an example. Similarly to metabolic activity heatmaps (see above), the times at which metabolic flux diagrams are generated are specified via the control variables

  - plotMetabolicFluxDiagrams,
  - plotMetabolicFluxDiagrams, and
  - plotAverageMetabolicFluxDiagrams.

  Similarly, for microbial flux diagrams

  - plotMicrobialFluxDiagrams,
  - plotMicrobialFluxDiagrams and
  - plotAverageMicrobialFluxDiagrams.

  Technical note: By default, chord widths are proportional to fluxes and arc lengths are determined by the total chord width within them. Alternatively, arc widths can be normalized to have equal length using the control flags normalizeArcsInMetabolicFluxDiagrams and normalizeArcsInMicrobialFluxDiagrams.

- MCM can save cell-metabolic FBA models with calculated constraints and fluxes in SBML file format [37] at times specified via the control variable saveCellMetabolicNetworksSBML (same syntax as for plotMetabolicActivityHeatmaps).
- If the flag saveNetworkStructure is set, MCM saves the community wide metabolic network as well as the implied gene-gene and metabolite-metabolite interaction networks, into common file formats (ARFF, BIOM [52], GEXF and SBML [37]). This can be useful for visualization with 3rd party tools [3, 94].
- If the flag checkTheoreticalSystemLimits is set, MCM tries to estimate maximum cell growth rates based on the cell-metabolic model structure[14].

The following MCM commands demonstrate typical choices regarding simulation output:

```
set output_timeSeriesAnalysis saveAndPlot # save and plot periodograms and autocorrelations
                                          # alternative choices are `skip' and `save'

set output_speciesSpecificMetabolism save   # save (but don't plot) reaction rates
                                            # and metabolite fluxes individually
                                            # for each focal species

set maxRARank 20               # maximum species rank to show in rank-abundance curves
set maxACRShift 10             # maximum time-lag to show in autocorrelations
set maxFourierFrequency 10     # maximum frequency to show in periodograms
set statisticsInterval [20:*]  # only use times>20 for statistical evaluation

set checkTheoreticalSystemLimits     # try to estimate maximum possible cell growth rates
                                     # might be useful for spotting bad FBA models

unset saveNetworkStructure             # don't save overall metabolic network structure

set   plotMetabolicPotentialHeatmap    # save heatmap of metabolic potential per species
set   plotMetabolicActivityHeatmaps start 0 step 5   # save metabolic activity heatmap
                                                     # every 5 days starting at day 0

set saveCellMetabolicNetworksSBML at 10  # save FBA models in SBML format at day 10
```

[14]These estimates are to be taken with a grain of salt.

```
set saveCellMetabolicNetworksSBML_onlyFocalSpecies  # only do so for focal cell species
```

The duration of the simulation, the size of the time series (i.e. the maximum number of recorded points during the simulation) as well as the integration time step can be controlled as follows:

```
set integrationTimeStep 0.01   # this controls the numerical accuracy of the simulation
set maxTimeSeriesSize   100    # only record at most that many points
set maxSimulationTime   20     # simulate 20 days
```

It is recommended to keep `integrationTimeStep` well below the typical time scales at which you expect your model variables (i.e. cell concentrations, metabolite concentrations and environmental variables) to change.

Technical note: MCM uses the explicit two-stage Heun integration scheme for all simulations. Particularly short time steps might be required near the boundary of the permissible state space, e.g. when substrate concentrations rapidly approach zero (discontinuous jumps of metabolic activity might be observed in that case). Near such boundaries, MCM will temporarily and iteratively refine (halve) the integration time step as needed. The maximum number of permissible refinements is set using the variable `maxIntegrationTimeStepRefinements`.

Technical note: Computation can be accelerated if MCM is allowed to use multiple CPUs (or CPU cores) for solving multiple FBA problems simultaneously, if available. This, of course, is only possible if the model comprises multiple species. To enable parallel (or *multithreaded*) computation, set the flag `parallel`. But see section 14.1.7 for possible issues.

7 Comparing and calibrating models to data

7.1 Introduction

Following a simulation, model predictions for the following quantities can be statistically evaluated against available time series data:

- metabolite concentrations,
- net community wide metabolite export rates,
- metabolite export rates per cell (averaged over cells using or producing them),
- net environmental (i.e. extracellular) metabolite production rates,
- gene concentrations,
- gene concentrations (dead+alive),
- community wide reaction rates,
- reaction rates per cell (averaged over cells capable of performing them),
- environmental (i.e. extracellular) reaction rates,
- cell concentrations,
- cell concentrations (dead+alive),
- cell (per capita) growth rates,
- environmental variables,
- observables.

Statistical comparisons are performed in terms of the log-likelihood of the observed data, but other measures of *goodness of fit* are also available. The log-likelihood is the probability density at the observed data, assuming a particular underlying stochastic model, typically split into a *deterministic* and an *error* (or *random*) part.

Each variable is assumed to exhibit either (a) a normal or (b) a log-normal error distribution, as specified beforehand by user. The deterministic part (or zero-noise limit) of a variable is given by the model prediction. More concretely, case (a) assumes that at any point in time t, any model variable V_i (e.g. NO_2 concentration) is related to its measured value $\tilde{V}_i$ via

$$\tilde{V}_i(t) = V_i(t) + \sigma_i \cdot \mathcal{E}_i(t), \tag{9}$$

where $\mathcal{E}_i(t)$ are uncorrelated standard-normal random variables and σ_i is the constant (but unknown) standard deviation of measurement errors (henceforth *error amplitude*). Similarly, case (b) assumes that

$$\ln \tilde{V}_i(t) = \ln V_i(t) + \sigma_i \cdot \mathcal{E}_i(t), \tag{10}$$

i.e. errors are normally distributed on a logarithmic scale. The latter error model is recommended for positive variables possibly spanning multiple orders of magnitude over time (such as cell concentrations), and is motivated by the central limit theorem (in the logarithmic domain) in the case of multiplicative products of several positive random variables. For example, measurements retrieved through a sequence of multiple (e.g. electronic, chemical, biological) amplification steps are subject to random errors at any point in the amplification sequence that are subsequently amplified further downstream [46]. In contrast to the widely used normal error model, the log-normal error model accounts for the heteroscedastic error structure expected for quantities that can range multiple orders of magnitude. In the above model, the standard deviation of the measurements $\tilde{V}_i$ scales linearly with the deterministic value V_i, with the scaling factor determined by σ_i. In both error models, the deterministic part is in fact the median of the random measurment.

The log-likelihood of a particular variable V_i, with measured values $\tilde{V}_{i1}, .., \tilde{V}_{iM}$ and predicted to have values $V_i(t_1), .., V_i(t_M)$ at times $t_1, .., t_M$, is given by

$$\mathrm{LL}_i = -M \ln \sqrt{2\pi\sigma_i^2} - \frac{1}{2\sigma_i^2} \sum_{m=1}^{M} \left[\tilde{V}_{im} - V_i(t_m) \right]^2 \tag{11}$$

in case of a normal error structure, and by

$$\mathrm{LL}_i = -M \ln \sqrt{2\pi\sigma_i^2} - \sum_{m=1}^{M} \ln \tilde{V}_{im} - \frac{1}{2\sigma_i^2} \sum_{m=1}^{M} \left[\ln \tilde{V}_{im} - \ln V_i(t_m) \right]^2 \tag{12}$$

in case of a log-normal error structure. MCM calculates the log-likelihood LL_i for each model variable for which time series data are available. The unknown error amplitudes σ_i are automatically estimated using maximum-likelihood estimation, i.e. by maximizing LL_i. Note that the last terms in Eq. (11) (or Eq. (12)) resemble the classical sum of squared errors between model predictions and data on a linear (or logarithmic) scale. Hence, the normalized log-likelihood, $\mathrm{NLL}_i = \mathrm{LL}_i / M$, might be seen as an average *goodness of fit* for the variable V_i evaluated on a linear (or logarithmic) scale. The overall log-likelihood of the model is the sum of log-likelihoods of all model predictions for which data is available. Maximizing the log-likelihood by suitable choice of unknown (or *free*) model parameters yields an estimate for those parameters (see section 7.3 on maximum-likelihood fitting).

It is also possible to provide data in a priori unknown (or *arbitrary*) units, such as cell concentrations measured in terms of optical densities with unknown calibration to actual cell counts. In that case, V_i is conceptually replaced by V_i/α_i in the above error models, where α_i is an unknown scaling factor also estimated using maximum-likelihood estimation. Hence, MCM also allows the calibration of unknown linear measurement units by fitting of cell-metabolic models. Obviously, such calibrations are only as reliable as the model is suitable for the system at hand.

Technical note: Likelihoods are, a priori, probability densities over permissible value space and hence depend on measurement units (see Eq. (11) and (12)). To allow for a comparison of log-likelihoods between different variables, MCM makes likelihoods unit-less by multiplying them with the arithmetic mean of the provided time series data. This rescaling has no effect on maximum-likelihood parameter estimation (see section 7.3).

7.2 Comparing simulations to data

Model predictions generated by a simulation (invoked by the runMCM command) are automatically compared to available time series data. Error models (either normal or logNormal, see the previous section) are specified in the MCM command line, e.g. as

```
# specify the error model for each variable class
# only 'normal' error structure is allowed for variables possibly becoming negative
set errorModel_MetaboliteConcentrations             logNormal
set errorModel_MetaboliteExportCommunityWide        normal
set errorModel_MetaboliteExportPerCell              none  # disable data comparison
set errorModel_MetaboliteProductionEnvironmental    normal
set errorModel_GeneConcentrations                   logNormal
set errorModel_GeneConcentrationsDeadAlive          logNormal
set errorModel_ReactionRatesCommunityWide           normal
set errorModel_ReactionRatesPerCell                 normal
set errorModel_ReactionRatesEnvironmental           normal
set errorModel_CellConcentrations                   logNormal
set errorModel_CellConcentrationsDeadAlive          logNormal
set errorModel_CellGrowthRates                      normal
```

click here for source code

The error model of environmental variables and observables is specified separately for each of them in the MC model files environment and observables, respectively (see sections 5.1.2 and 5.5.2).

Output variables can be excluded from any data comparison by specifying their error model as none. Available time series data must be provided to MCM in a specific format, in a data directory specified by the MCdataDir option, e.g.

```
set MCdataDir "data/bioreactor"
```

Time series data must be provided in a separate plain text file for each variable. Each data file must be named exactly as the corresponding environmental variable, metabolite, reaction, species or observable (e.g. "NH4", no extension) and placed within a subdirectory in MCdataDir corresponding to the general observable class (see Table 6).

Table 6: Data sub-directories in which time series data must be placed, depending on the kind of variable. In the examples, MCdataDir is assumed to be data. See section 7.2 for details.

sub-directory	example
MetaboliteConcentrations	data/MetaboliteConcentrations/NH4
MetaboliteExportCommunityWide	data/MetaboliteExportCommunityWide/NH4
MetaboliteExportPerCell	data/MetaboliteExportPerCell/NH4
MetaboliteProductionEnvironmental	data/MetaboliteProductionEnvironmental/NH4
GeneConcentrations	data/GeneConcentrations/amo
GeneConcentrationsDeadAlive	data/GeneConcentrationsDeadAlive/amo
ReactionRatesCommunityWide	data/ReactionRatesCommunityWide/amo
ReactionRatesPerCell	data/ReactionRatesPerCell/amo
ReactionRatesEnvironmental	data/ReactionRatesEnvironmental/amo
CellConcentrations	data/CellConcentrations/Ecoli
CellConcentrationsDeadAlive	data/CellConcentrationsDeadAlive/Ecoli
CellGrowthRates	data/CellGrowthRates/Ecoli
Environment	data/Environment/pH
Observables	data/Observables/AOB

For example, time series for NH4 concentrations, community-wide NH4 export, the environmental variable pH and the observable AOB should be saved in the following locations, respectively:

```
data/bioreactor/MetaboliteConcentrations/NH4
data/bioreactor/MetaboliteExportCommunityWide/NH4
```

```
data/bioreactor/Environment/pH
data/bioreactor/Observables/AOB
```

See section 14.3.6 for data file format requirements. Data values are assumed to be given in the same physical units as used by MCM for the corresponding quantities (see section 5.13.4) or, in the case of environmental variables and observables, as specified by the MC model. Alternatively, as mentioned in section 7.1, data can also be provided in arbitrary (unknown) units, in which case the postfix " [au]" must be appended to their file name. For example, if `Nitrosomonas` cell concentrations are measured in optical density units, these should be saved in the following location instead:

```
data/bioreactor/CellDensities/Nitrosomonas [au]
```

If both data files (e.g. `Nitrosomonas` as well as `Nitrosomonas [au]`) are provided, the latter is ignored. Data directories may also contain other unrelated files. Setting `MCdataDir` to empty (this is the default) will disable any data comparisons.

Model predictions are linearly interpolated between recorded time points during the simulation and compared at the exact time points for which data are provided. No extrapolation and thus no comparison is done for data points outside the simulated time frame. Data points can also be excluded using the control variable `statisticsInterval`. The latter option might be useful if one only wants to evaluate the model within a particular time interval, for example after reaching an equilibrium. Multiple values at identical time points are allowed and are either evaluated as independent data points or averaged, depending on the flag `averageAmbiguousData`. In the case of a log-normal error structure, all non-positive data points or time points at which the model prediction is non-positive, are ignored.

For each evaluated model observable V_i, MCM estimates the error standard deviation σ_i (and data units, if unknown), calculates the log-likelihood and plots an overview of the time series data and the simulated time course. MCM also shows the estimated 95% centile region around the model prediction. MCM calculates the overall model log-likelihood and plots an overview histogram of the normalized log-likelihoods for all variables. Similarly, MCM calculates and plots an overview histogram of the average normalized squared residuals[15] as well as the coefficients of determination (R^2) for each observable (see Fig. 35 for an example). All related output is written to the directory `comparison_to_data`. For easy further processing by other pipelines (e.g. likelihood optimizers calling MCM), the overall log-likelihood and the number of evaluated data points are also written into the separate file `loglikelihood.txt`.

7.3 Estimating (fitting) unknown model parameters

In the presence of measured data, the overall log-likelihood (LL) of the model (see section 7.1) is a measure for the *goodness of fit* to the data. Maximizing the LL by suitable choice (*fitting*) of unknown model parameters yields statistical estimates for these parameters, so called *maximum-likelihood* (ML) *estimates*. In general, i.e. under mild regularity conditions, ML estimates approach an increasingly peaked normal distribution around the *true* (but unknown) parameter values. The inverse observed Fisher information, $\left(-\frac{\partial^2 \mathrm{LL}}{\partial \mathbf{q}^2}\right)^{-1}$ (where LL is the overall log-likelihood and $\mathbf{q}$ are the free parameters), evaluated at the fitted parameter values, is a consistent estimator of the asymptotic covariance matrix of the ML estimates [17, §10.4]. As such, the observed Fisher information can be used to estimate confidence intervals for each fitted parameter [8].

Technical note: For the normal and log-normal error models (see section 7.1), ML estimation resembles conventional least-squares estimation on a linear and logarithmic scale [59], respectively, if one assumes a common error amplitude for all measurements. While convenient (because numerical minimization of squared errors is easy), this assumption is unjustified as soon as one considers multiple quantities, potentially with different physical units. ML estimation weights errors with respect to the estimated intrinsic error amplitude σ_i of each observable, while also penalizing overestimates for σ_i.

MCM automates the maximum-likelihood parameter estimation as well as the calculation of confidence intervals. Parameters to be fitted need to be specified as symbolic, as described in section 5.7. MCM uses generic optimization routines to maximize the fitting objective via small stepwise variations of symbolic

[15]That is, the sum of squared residuals normalized by the squared arithmetic data mean and the number of evaluated data points.

parameters and repeated simulations. If the flag `fit_calculateConfidenceIntervals` is set, MCM will also estimate confidence intervals for the fitted parameters, as described above. The observed Fisher information is calculated by approximating the second derivative of the LL (known as the *Hessian*) using finite differences [55]. Finite difference steps are repeatedly halved (*refined*) as required to achieve a satisfactory accuracy[16].

By default, MCM maximizes the normalized log-likelihood (NLL) instead of the regular log-likelihood, to account for varying numbers of evaluated data points across different simulations. Typically, however, the number of evaluated data points will be constant over multiple fitting iterations, particularly in the proximity of an optimum, yielding the same parameter estimates as a maximization of the log-likelihood would. To maximize the non-normalized log-likelihood instead, set the control variable `fit_objective` to `LL`. Alternatively, if `fit_objective` is set to `meanR2`, fitting is performed by maximizing the mean coefficient of determination (R^2) of model predictions (this objective function, however, does not support the estimation of confidence intervals).

Parameter fitting is performed using the `fitMCM` command. `fitMCM` requires the presence of time series data for at least one model observable (see section 7) and at least one symbolic parameter to fit. Observables whose error model is set to `none` are not considered. Parameters specified as `fixed` are not fitted; rather, their default value is used throughout. Fitted parameters are only varied within their `minimum` and `maximum` bounds (which must be finite). Only data points within the interval specified by `statisticsInterval` are considered.

Note that parameter fitting, the so called *inverse problem*, can be littered with surprises and technical obstacles, particularly for nonlinear high-dimensional models [90]. MCM provides several technical fitting options to experiment with if necessary. To avoid the danger of reaching a locally but not globally optimal fit, it is advised to start the fitting at different parameter values. By default, MCM starts at the `default` parameter values. If the control variable `fit_randomRepeats` is set to a positive number, MCM will repeat the fitting that many times starting at random parameter values, chosen independently and according to their probability distribution (see section 5.7). MCM will then choose the fitting outcome that yielded the best fit. If `fit_randomRepeats` is `0`, the probability distributions of parameters are irrelevant.

Fitting options (additional to standard simulation options) are exemplified below:

```
# the following options control the maximum effort put into fitting
set   fit_maxSimulations 500     # stop fitting algorithm beyond this number of simulations
set   fit_randomRepeats  5       # additional fitting attempts
                                 # starting at random parameter values
set   fit_maxTime        1000    # stop after 1000 seconds of execution time



# the following options control the targeted fitting accuracy
set   fit_objAbsTolerance   1e-5   # find NLL maximum until within this tolerance
set   fit_PrelativeTolerance 1e-5   # fit parameters to within this
                                    # relative distance from their optimal value
set   fit_PrelativeInitialStep 1e-2   # first attempted parameter variation
                                      # (relative to their value)


# the following options control the calculation of confidence intervals after fitting
set   fit_calculateConfidenceIntervals   # also include confidence intervals when fitting
set   fit_confidenceLevel 0.95           # choose confidence level for confidence intervals


# change the fitting algorithm (the default choice is usually the best one)
# available options are SBPLX, COBYLA, BOBYQA, NelderMead
# see http://ab-initio.mit.edu/wiki/index.php/NLopt_Algorithms
set   fit_algorithm SBPLX
```

[16]However, finite difference steps for parameters will not be refined to below about the fitting error tolerance (as determined by `fit_PrelativeTolerance`). Note that machine precision also sets a lower bound on the permissible finite differences step [66, chapter 5.7].

```
# define objective function for fitting
set fit_objective NLL # available options are NLL, LL, meanR2

# the following options control the amount of output during fitting
unset fit_saveIntermediateSimulations   # don't save simulation results for
                                        # intermediate (i.e. non-fitted) parameter values
unset fit_saveIntermediateDataComparisonResults  # don't save data comparison reports for
                                                 # intermediate (non-final) parameter values

set   fit_saveFinalOfEachRepeat  # for each fitting attempt, save the final simulation
set   fit_plotProgress   # for each fitting attempt, plot an overview of the fitting progress

set   fit_verbosity 1    # control verbosity (i.e. amount of details printed during fitting)
                         # a higher number (<=4) means more details
                         # 0 means minimal reports
```

click here for source code

The following two parameters affect the finite difference approximation of derivatives, used to calculate the observed Fisher information:

```
set FD_PrelativeStep 1e-4     # initially attempt to estimate partial derivatives
                              # with this relative finite difference step
set FD_maxRefinements 10      # don't refine finite difference steps more than 10 times
```

In principle, any number of parameters can be estimated simultaneously. Furthermore, fitted parameters need not be directly connected to the data used for calibration, as long as a change in the parameters influences the variables that are being compared to the data. Nevertheless, typical limitations of inverse problems [90] apply: (i) The fitting problem should not be degenerate (i.e. all parameters must influence the "goodness of fit" measure being optimized). While such parameters might still be fitted, the calculation of confidence intervals will likely fail. (ii) Fitted parameters should be independent, i.e. alternative similar parameter combinations must not yield the same optimum. Mathematically, this means that the gradient matrix at the optimum must have full rank. (iii) In order to avoid over-fitting, the number of data points should be much higher than the number of parameters to be fitted. In many cases good knowledge of the system or previous literature may be required to identify implausible calibrations.

The fitting routine writes a complete report into the file `fit_report.txt`. The fitted MC model is simulated one last time and simulation results and data comparison reports are saved into the `run_final` directory. A new `parameters` file (named `parameters_fitted`) is also generated, with the original `default` values replaced by the fitted values. This file can directly replace the original `parameters` file for subsequent simulations of the fitted MC model. If the flag `fit_plotProgress` is set, the LL, NLL and mean R^2 values encountered during fitting are plotted to the file `fit_progress.pdf` (see Fig. 33 for an example). If confidence intervals are calculated, MCM also plots a heatmap of the estimated parameter covariance matrix (see Fig. 34 for an example). The appearance of the latter can be adjusted as described in section 14.3.4. For a simple fitting example see section 2, for a more advanced example see section 12.

8 Sensitivity analysis

8.1 Sensitivity of model predictions

MCM can perform local sensitivity analysis (LSA) of an MC model with respect to symbolic parameters using normalized local sensitivity coefficients (NLSC) [11]. NLSCs compare the relative changes (averaged over time) in model variables (V_i) to the relative changes of individual parameters (p_j) by means of partial derivatives, evaluated at some default parameter choice:

$$\mathrm{NLSC}_{ij} = \left\| \frac{p_j}{V_i} \cdot \frac{\partial V_i}{\partial p_j} \right\|_2 . \tag{13}$$

Here, $\|f\|_q = \sqrt[q]{\frac{1}{t_2-t_1}\int_{t_1}^{t_2}|f(t)|^q\ dt}$ is the L^q-norm[17] of any function of time, $f(t)$, over a considered time interval $[t_1, t_2]$ (note that MCM predictions are time courses of particular quantities). Hence, NLSC_{ij} is a measure for the relative effects of small changes of the parameter p_j on the observable V_i. Note that in Eq. (13) every perturbation of the observable V_i is compared to its original unperturped value at the same time point (this is called a *pointwise normalization*), making NLSCs particularly sensitive to changes at lower values of V_i (e.g. near the onset of growth, if V_i is a cell concentration). Alternatively, one can consider the modified version

$$\text{NLSC}_{ij} = \frac{|p_j|}{\|V_i\|_1} \cdot \left\| \frac{\partial V_i}{\partial p_j} \right\|_2, \tag{14}$$

where changes of the observable are first averaged and then compared to the averaged observable magnitude, $\|V_i\|_1$. To toggle between the two NLSC versions, use the MCM flag `SA_normalizePointwise`.

Similarly, MCM can perform global sensitivity analysis (GSA) of an MC model using normalized global sensitivity coefficients (NGSC). The latter measure the relative changes of model predictions as individual symbolic parameters range from their `minimum` to their `maximum`, while all other parameters are fixed to their `default` values:

$$\text{NGSC}_{ij} = \left\| \frac{V_i(\text{at maximum } p_j) - V_i(\text{at minimum } p_j)}{V_i(\text{at default } p_j)} \right\|_2. \tag{15}$$

Similarly to LSA, one can modify the flag `SA_normalizePointwise` to toggle between pointwise or non-pointwise normalization.

LSA and GSA are performed using the commands `LSAMCM` and `GSAMCM`, respectively. Both LSA and GSA require the specification of an MC model with at least one non-`fixed` symbolic parameter (see section 5.7). In the case of LSA, non-fixed parameters must have a non-zero `default` value. In the case of GSA, non-`fixed` parameters need to vary within a non-trivial interval (as specified by their `minimum` and `maximum`). The number of simulations required increases linearly with the number of perturbed parameters.

For MCM-generated time series, $\|\cdot\|_2$ is approximated using a trapezoid integration scheme over the considered time period. Using the control variable `statisticsInterval` one can control the time range within which to evaluate the model's sensitivity:

```
set statisticsInterval [10:20] and [100:*]   # days 10 to 20, as well as day 100 and after
```

Partial derivatives, as used for the NLSCs, are approximated numerically by finite differences [55]. Finite difference steps are repeatedly halved (*refined*) if needed to achieve satisfactory accuracy. The following two parameters affect the finite difference approximation of derivatives:

```
set FD_PrelativeStep 1e-3     # initially attempt to estimate partial derivatives
                              # with this relative finite difference step
set FD_maxRefinements 10      # don't refine finite difference steps more than 10 times
```

Any of the model predictionslisted in section 7.1 can be included in a sensitivity analysis using appropriate flags and control variables, as demonstrated below:

```
set    SA_includeMetaboliteConcentrations
set    SA_includeMetaboliteExportCommunityWide
set    SA_includeMetaboliteExportPerCell
set    SA_includeMetaboliteProductionEnvironmental
set    SA_includeReactionRatesCommunityWide
set    SA_includeReactionRatesPerCell
set    SA_includeReactionRatesEnvironmental
unset  SA_includeCellConcentrations           # do not include cell concentrations
unset  SA_includeCellGrowthRates              # nor cell growth rates
set    SA_includeCellConcentrationsDeadAlive
set    SA_includeGeneConcentrations
```

[17] A generalization of the euclidean metric.

```
set    SA_includeGeneConcentrationsDeadAlive
unset  SA_includeEnvironment                  # do not include environmental variables
unset  SA_includeObservables                  # do not include observables

set    SA_cellBiodiversity all                # include biodiversity of all cell species
set    SA_geneBiodiversity focals             # include biodiversity of focal genes
```

click here for source code

MCM only considers focal environmental variables, metabolites, reactions or species for sensitivity analysis (see section 5.6 on declaring focals). The following options control the amount of output during sensitivity analysis:

```
set    SA_verbosity 2   # set this to 0 to suppress all output (except errors)
unset  SA_saveIntermediateSimulations  # do not save any intermediate simulation results
                                       # except with the default parameter values
```

A summarizing report is written to the file `LSA_report.txt`. MCM also generates a heatmap of the sensitivity matrix, the appearance of which can be modified as described in section 14.3.4. See figures 18 and 23 for example LSA and GSA heatmaps generated by MCM. GSA will also produce time series plots of focal environmental variables, metabolites, reactions, species and observables and their responses to parameter changes (see Fig. 24 for an example).

Technical note: Some MC models can be very sensitive to certain parameters, to the point where numerical differentiation becomes problematic. Similarly, if an observable depends very weakly on a parameter, numerical errors can mask any existing linear responses, particularly at small difference steps. In both cases, the finite differences scheme can fail to produce an accurate estimate of derivatives. An affine-linear extrapolation of previous finite difference approximations is then used to estimate the *true* derivatives. This will be indicated in the LSA or GSA report file.

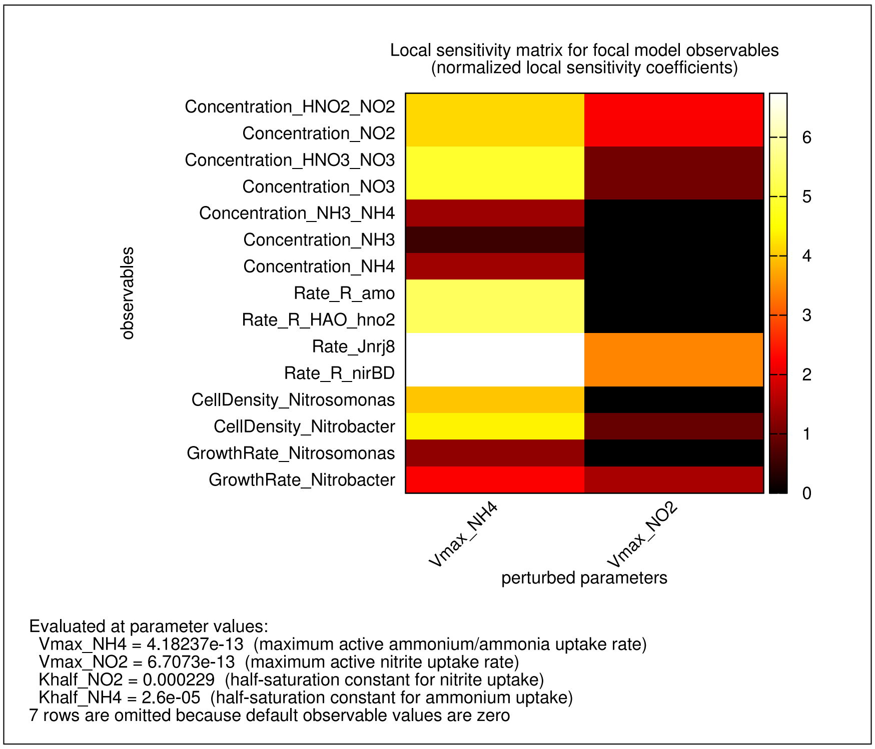

Figure 18: Example of a local sensitivity heatmap generated by MCM for a two-species microbial nitrifier community feeding on ammonium and nitrite. A brighter color corresponds to a greater normalized local sensitivity coefficient.

8.2 Sensitivity of log-likelihoods

When time series data are available for comparison, MCM can perform local and global sensitivity analysis of the log-likelihoods of model predictions (see section 7 on log-likelihoods and data comparisons), similarly to the sensitivity analysis described for model predictions in section 8.1. Local sensitivity analysis of the log-likelihoods, performed using the command `LSAMCM_LL`, involves the calculation of the partial derivatives with respect to symbolic model parameters, evaluated at some default parameter choice:

$$|p_j| \cdot \frac{\partial \, \mathrm{LL}_i}{\partial p_j}. \tag{16}$$

Partial derivatives are rescaled by the `default` value of the perturbed parameter to account for different scales among parameters. Hence, the values (16) define a local sensitivity matrix which quantifies the change of the log-likelihoods LL_i as a result of small relative changes of model parameters p_j. Partial derivatives are calculated using finite differences with repeated step refinements, as described in section 8.1.

Similarly, global sensitivity analysis of the log-likelihoods is performed using the command `GSAMCM_LL` and quantifies the changes of the LL_i as model parameters are varied from their `minimum` to their `maximum` values:

$$\mathrm{LL}_i(\text{at maximum } p_j) - \mathrm{LL}_i(\text{at minimum } p_j) \tag{17}$$

Both `LSAMCM_LL` and `GSAMCM_LL` require an MC model with at least one free (i.e. non-`fixed`) symbolic parameter (see section 5.7). `LSAMCM_LL` requires that non-fixed parameters have non-zero `default` values. `GSAMCM_LL` requires that non-fixed parameters have a non-trivial range (as defined by their `minimum` and `maximum`). The `distribution` of symbolic parameters is irrelevant. Both `LSAMCM_LL` and `GSAMCM_LL` require that data be available for at least one of the quantities listed in section 7.1 for which the error model is not set to "`none`". For example, the following commands tell MCM to only consider log-likelihoods of metabolite concentrations (for which data is available):

```
set errorModel_MetaboliteConcentrations          logNormal
set errorModel_MetaboliteExportCommunityWide     none
set errorModel_MetaboliteExportPerCell           none
set errorModel_MetaboliteProductionEnvironmental none
set errorModel_GeneConcentrations                none
set errorModel_GeneConcentrationsDeadAlive       none
set errorModel_ReactionRatesCommunityWide        none
set errorModel_ReactionRatesPerCell              none
set errorModel_ReactionRatesEnvironmental        none
set errorModel_CellConcentrations                none
set errorModel_CellConcentrationsDeadAlive       none
set errorModel_CellGrowthRates                   none
```

Note that the `error model` of environmental variables and observables is specified separately for each of them (see sections 5.1.2 and 5.5.2).

The overall log-likelihood of the model is always included in the analysis. The entire analysis is repeated for the normalized log-likelihoods NLL_i (the log-likelihoods divided by the number of evaluated data points, per observable), allowing a comparison of the effects of parameter variation on the *goodness-of-fit* of various model observables. A summarizing report is written to `LSA_LL_report.txt` or `GSA_LL_report.txt`. MCM also generates heatmap plots of the local or global sensitivity matrix (see Fig. 31 for an example). The appearance of heatmaps can be modified as described in section 14.3.4. Intermediate simulation are saved if the flag `SA_LL_saveIntermediateSimulations` is set. The amount of information printed out by MCM during computation is set using the control variable `SA_LL_verbosity`. See section 12 for a complete example that includes an LSA and GSA of the log-likelihoods.

9 Uncertainty analysis

In certain cases the parameters of an MC model are not precisely known, but are assumed to be distributed according to some probability distribution. The latter can be, for example, the posterior of a preceding bayesian parameter estimation. In that case, the behavior of the microbial community is stochastic as well. Stochasticity in model predictions can also emerge from intrinsic stochastic dynamics, e.g. when environmental variables or environmental metabolite fluxes are stochastic processes (see sections 5.1 and 5.2.2, respectively)

The projection of parameter uncertainty or dynamic stochasticity to stochasticity in model predictions is called *uncertainty analysis* (UA). MCM performs UA using repeated independent simulations while randomly sampling uncertain parameters. Generated simulations are statistically evaluated to estimate the resulting probability distribution of observables across time. Any non-`fixed` symbolic parameter is considered uncertain, and its distribution is specified via the `distribution` tag in the `parameters` file (see section 5.7). For example, the following entries define two uncertain symbolic parameters, assumed to be log-uniformly and uniformly distributed, respectively, between their `minimum` and `maximum`:

```
Vmax_NH3
    description:  maximum active ammonium/ammonia uptake rate
    default:      4.6e-13
    minimum:      1e-14
    maximum:      1e-11
    distribution: logUniform
    fixed:        no

Vmax_NO2
    description:  maximum active nitrite uptake rate
    default:      1.1e-12
    minimum:      1e-13
    maximum:      1e-10
    distribution: uniform
    fixed:        no
```

UA is invoked using the command `UAMCM` and requires only an MC model. Of course, UA only makes sense if the model either includes uncertain symbolic parameters and/or dynamic stochasticity. The number of Monte Carlo trials used for UA is specified by the control variable `UA_MonteCarloTrials`. Individual groups of variables can be included or excluded using appropriate flags, as demonstrated below:

```
set    UA_includeMetaboliteConcentrations
set    UA_includeMetaboliteExportCommunityWide
set    UA_includeMetaboliteExportPerCell
set    UA_includeMetaboliteProductionEnvironmental
set    UA_includeReactionRatesCommunityWide
set    UA_includeReactionRatesPerCell
set    UA_includeReactionRatesEnvironmental
unset  UA_includeCellConcentrations            # do not include cell concentrations
unset  UA_includeCellConcentrationsDeadAlive   # nor cell concentrations (dead+alive)
unset  UA_includeCellGrowthRates               # nor cell growth rates
set    UA_includeGeneConcentrations
set    UA_includeGeneConcentrationsDeadAlive
unset  UA_includeEnvironment                   # do not include environmental variables
unset  UA_includeObservables                   # do not include observables

set    UA_cellBiodiversity focals              # include biodiversity of focal cell species
set    UA_geneBiodiversity none                # omit biodiversity of genes
```

click here for source code

The estimated distribution for each variable is plotted in the file `UA.pdf` in the form of discrete percentiles around the median (see figures 13 and 25 for examples). The maximum shown percentile (between 0 and 1) is controlled via `UA_maxCentile`, while the color palette is controlled via `UA_colorPalette`. MCM will highlight the case corresponding to the default parameter values, unless the flag `UA_showDefault` is

unset. Individual Monte Carlo simulations are only saved if the flag `UA_saveIntermediateSimulations` is set. The amount of information printed out during UA is controlled via `UA_verbosity`.

Technical note: Since UA is based on random Monte Carlo simulations, the outcome will vary each time UA is performed, particularly when `UA_MonteCarloTrials` is low. To ensure independent results among repeated UAs, you should randomize MCM's random number generator prior to `UAMCM` using the command `seed`.

10 Example 1 - Simulating a single-species model

In the following step-by-step example, we will setup and run a single-species simulation of *Escherichia coli* strain K-12 MG1655 growing on minimal medium. We will be using the metabolic model iAF1260 by Feist et al. [24], obtained from the group's website (file `Ec_iAF1260_flux1.xml` as of Sept. 13, 2014; potentially also included with this manual). Oxygen will be the limiting resource, and in the course of its depletion cell metabolism will shift from aerobic respiration to fermentative growth, producing ethanol as a byproduct. Eventually, increased ethanol levels will lead to stagnation of growth and culture death.

10.1 Configuring the MC model

We start by converting the *E. coli* SBML model file (`Ec_iAF1260_flux1.xml`) into a draft MC model compatible with MCM, using the included python script `micog` (see section 5.14 for details). Since we are only converting one SBML, our MC model will consist of one cell species including all reactions. In the terminal the appropriate command might look as follows:

```
micog.py -i Ec_iAF1260_flux1.xml\
         -o microbial_community_01\
         --output_rate_units mol_per_cell_per_day\
         --objective original\
         --cell_mass 2.8e-13\
         --cell_concentration 1e3\
         --environmental_metabolite_dynamics "concentration 0"
```

click here for source code

The first two options (`-i` and `-o`) define the input file and output directory, respectively. The option `-output_rate_units` specifies the rate units to be used for the MC model (this must always be `mol_per_cell_per_day`). We set `-objective` to `original` because iAF1260 already defines the objective in terms of biomass synthesis. Since iAF1260 defines rates with respect to dry biomass, we need to provide `micog` with the dry cell mass (2.8×10^{-13} g) in order to convert these to cell-specific rates (as required by MCM). The initial cell concentration is set to 10^3, although this can easily be changed later on. Finally, we're providing a default value for the `environmental dynamics` of metabolites: In our case all metabolites are by default absent from the environment (see section 5.2.2 for more options). However, several metabolites can still be taken up by cells, as defined in the original SBML file. Individual metabolite dynamics and uptake kinetics will be manually adjusted below.

The above command should produce a directory `microbial_community_01` with a draft MC model configuration, including the files `environment`, `metabolites`, `reactions` and `species`. Let us adjust this model to our needs. In the `species` file, make the cell's `life time` dependent on ethanol concentration (maximum life time of 19.9 days [71] and a doubled mortality at 1.68×10^{-4} mol/L ethanol, i.e. 1% alcohol ABV), and let's also improve the species' name:

```
E_coli
    initial concentration: 1e3
    mass: 2.8e-13
    life time: 19.9 inhibited_by M_etoh_e 1.68e-4
..
```

In the metabolites file, adjust the glucose's max active uptake/export rate, environmental dynamics and environmental dynamics as follows:

```
M_glc_D_e
    description: M_D_Glucose_C6H12O6
    max active uptake rate: Monod 7.056e-14 0.38e-6
    max active export rate: unlimited
    max passive uptake rate: 0
    max passive export rate: 0
    environmental dynamics: initial 0.0556 flux microbial_export
```

click here for source code

The initial glucose concentration is chosen to match minimal growth medium with 10 g glucose/L, while the uptake kinetic parameters are taken from Varma and Palsson [93] and Owens and Legan [58]. Note that the iAF1260 FBA model includes two formal metabolites representing glucose, M_glc_D_e (*external*) and M_glc_D_p (*periplasmic*). Such a distinction is common for transported metabolites in compartmentalized FBA models, and the "_e" version is conventionally (but not always) the one that is actually exchanged with the environment.

Similarly, adjust the oxygen uptake rate limits and environmental dynamics:

```
M_o2_e
    description: M_O2_O2
    max active uptake rate: Monod 1.008e-13 121e-9
    max active export rate: unlimited
    max passive uptake rate: 0
    max passive export rate: 0
    environmental dynamics: initial 0.217e-3 flux microbial_export
```

click here for source code

Oxygen is initially at 100% saturation with the atmosphere (at 37° C), while uptake kinetic parameters are taken from Varma and Palsson [93] and Stolper et al. [86]. Because we want to keep track of the produced acetate (M_ac_e), formate (M_for_e), ethanol (M_etoh_e) and succinate (M_succ_e), set the environmental dynamics for each of these metabolites to "initial 0 flux microbial_export".

Finally, let us specify the metabolites and reactions that we're particularly interested in (see section 5.6), by adjusting the focals file to something like the following:

```
file_version: 1.3 # do not remove, move or change this line

FOCAL_METABOLITES
    M_glc_D_e:glucose
    M_o2_e:oxygen
    M_etoh_e:ethanol
    M_ac_e:acetate
    M_for_e:formate
    M_succ_e:succinate

FOCAL_REACTIONS
    R_Ec_biomass_iAF1260_core_59p81M: biomass synthesis
    R_PFK:phosphofructokinase, glycolisis
    R_PFL:pyruvate formate lyase, fermentation
    R_FRD2:fumarate reductase, fermentation
    R_ALCD2x:ethanol dehydrogenase, fermentation (when reversed)


FOCAL_SPECIES
* # all species are focal
```

click here for source code

10.2 Setting up the MCM control script

Now that we've set up our MC model, we proceed by creating a simple MCM script that will contain all necessary commands to run our simulation. Next to the `microbial_community_01` directory (which contains our MC model), create a plain text file (let's call it `script_01`) containing the following:

```
# specify the MC model directory
set MCmodelDir "microbial_community_01"

# create a new non-existing output directory
setodnew "simulations_01/run_"

saveScript    # save a backup copy of this script
saveMCmodel   # save a backup copy of the MC model directory

# adjust simulation parameters
set integrationTimeStep 0.01              # integration time step in days
set maxIntegrationTimeStepRefinements 2   # allow up to 2 time step refinements
set maxTimeSeriesSize   100               # record about 100 time points
set maxSimulationTime   4                 # simulate four days

# run simulation
runMCM

# open output directory after simulation has finished quit MCM
openod
quit
```

click here for source code

See section 4 for details on MCM control scripts, and section 6 for details on running simulations.

10.3 Running the simulation

Now that we have configured our MC model and prepared our MCM script, it's time to cut to the chase: In your command line, call MCM with the script path as an additional argument:

```
MCM script_01
```

This should execute all commands in the script and result in the output directory `simulations_01/run_01` (or `simulations_01/run_<N>` if you repeat this action N times). All simulation results and reports are saved to this output directory (see section 13 for an overview of output files). Temporal profiles of summary quantities such as community-wide reaction rates are saved into the subdirectory `simulation_summary`, while species-specific output (irrelevant for our single-species model) is saved into `simulation_species_specific`. The predicted microbial export rates (per cell), metabolite concentrations, reaction rates (per cell) and cell population dynamics are shown in figures 19, 20, 21 and 22, respectively.

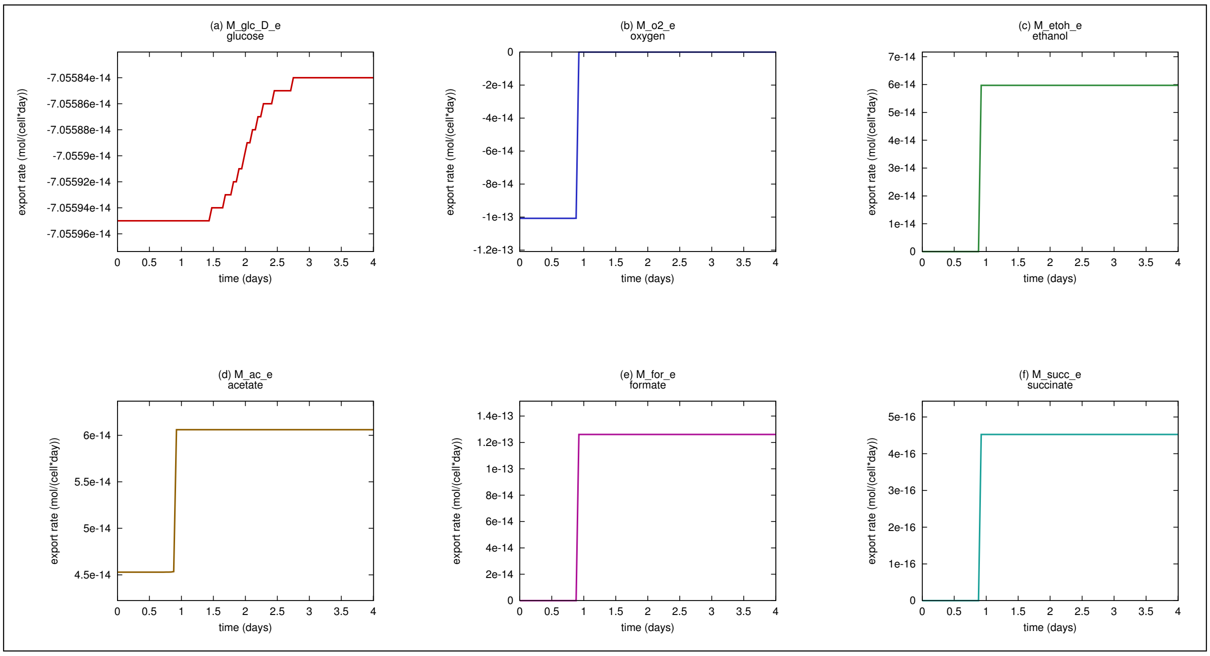

Figure 19: Metabolite export rates (per cell), predicted for the *E. coli* culture model described in section 10. Observe the transition to ethanol-producing fermentative growth after 1 day, triggered by oxygen depletion (see Fig. 20b). Excerpt from the output file `metabolite_net_export_per_cell.pdf`.

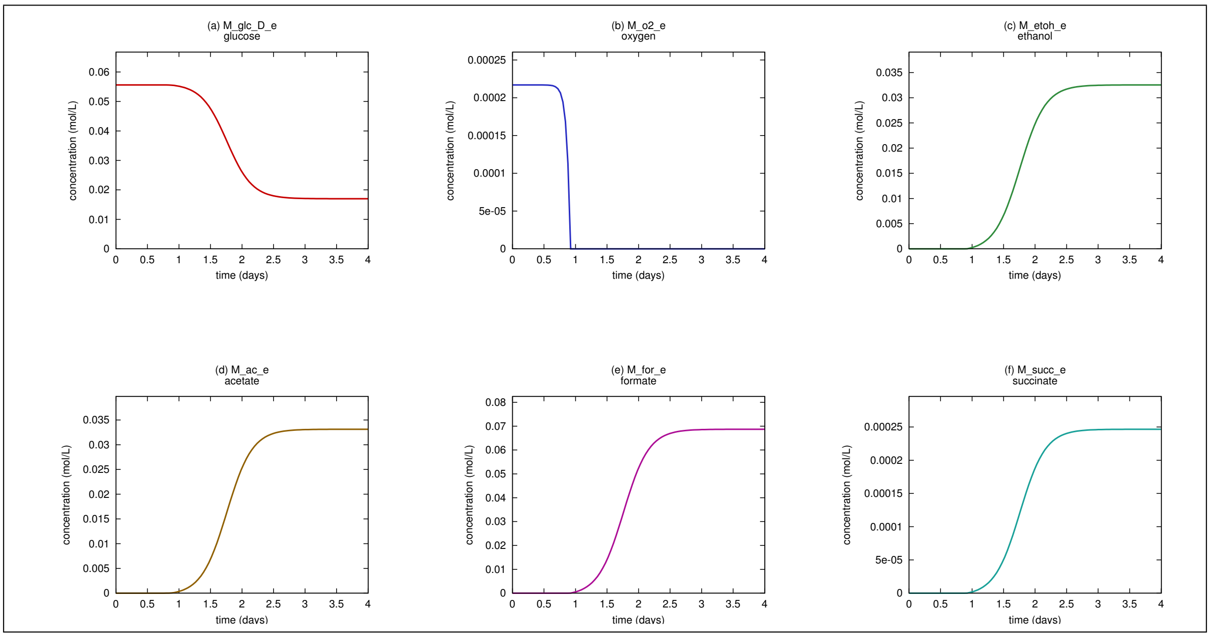

Figure 20: Metabolite concentrations, predicted for the *E. coli* culture model described in section 10. Observe the onset of fermentative ethanol production as oxygen gets depleted. Excerpt from the output file `metabolite_concentrations.pdf`.

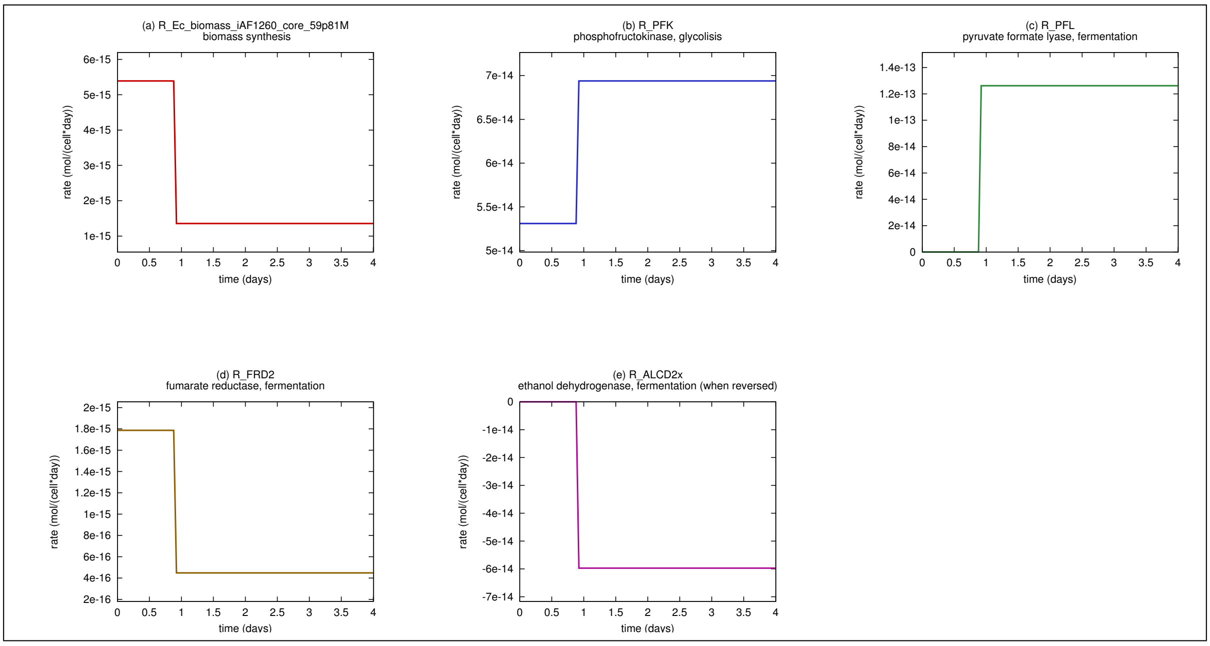

Figure 21: Reaction rates (per cell), predicted for the *E. coli* culture model described in section 10. Excerpt from the output file `reaction_rates_per_cell.pdf`.

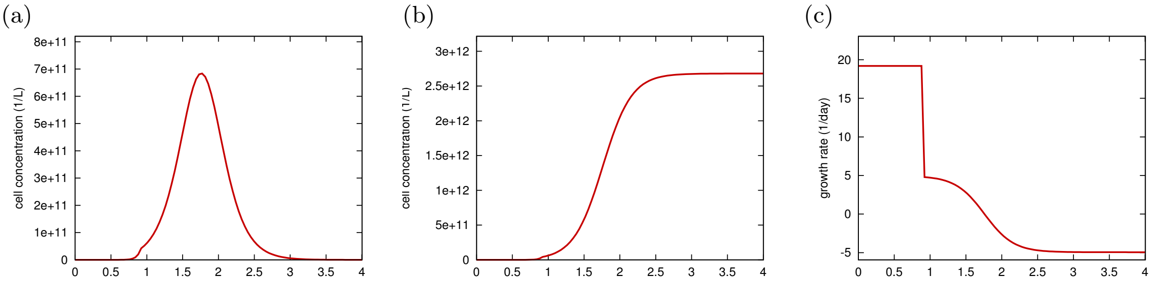

Figure 22: Cell concentrations (a), cell concentrations dead+alive (b) and cell growth rates (c), predicted for the *E. coli* culture model described in section 10. Observe the diauxic growth phase until day 1.5, characterized by a transition from aerobic to fermentative growth after 1 day (Fig. a and c). The rapid decline of living cells after day 1.5 is triggered by the sudden ethanol accumulation (Fig. 20c), resulting from fermentative ethanol production (Fig. 19c). While glucose concentration does decrease (Fig. 20a), it is not a limiting factor to cell growth, as seen from the unchanged glucose uptake rate limits (plot not shown). Excerpts from the output files `cell_concentrations.pdf`, `cell_concentrations_dead_alive.pdf` and `cell_growth_rates.pdf`.

11 Example 2 - Sensitivity and uncertainty analysis of the *E. coli* model

In this example we demonstrate the use of symbolic parameters (see section 5.7) to perform an automatic local and global sensitivity analysis, as well as an uncertainty analysis of the *E. coli* model from the previous example (section 10). More precisely, we will look at the responses of several model predictions (e.g. cell concentrations, final ethanol concentration) to variations of the maximum glucose uptake rate, the cell's maximum life expectancy and the ethanol half-inhibition concentration. For general information on sensitivity analysis and uncertainty analysis see sections 8 and 9, respectively.

11.1 Defining the symbolic parameters

Create a copy of the previous MC model directory (section 10) and call it `microbial_community_02`. Inside the latter, modify the copied `parameters` file to define the following three symbolic parameters:

```
file_version: 1.3

Vmax_glucose
    description:  maximum glucose uptake rate
    default:      7.056e-14
    minimum:      5e-14
    maximum:      9e-14
    fixed:        no


life_expectancy
    description:  expected life duration for starving cells
    default:      19
    minimum:      10
    maximum:      30
    fixed:        no


Kinh_ethanol
    description:  ethanol half-inhibition constant
    default:      1.68e-4
    minimum:      1.3e-4
    maximum:      1.9e-4
    fixed:        no
```

click here for source code

Note that all three parameters have been defined as non-`fixed`. The default value for the maximum glucose uptake rate, `Vmax_glucose`, has been chosen according to Varma and Palsson [93]. The default value for the maximum cell life time under starvation has been chosen according to Reeve et al. [71]. The ethanol half-inhibition values have been chosen somewhat arbitrarily within plausible bounds. In the `metabolites` file, replace the numerical kinetic parameter for maximum glucose uptake rate with the symbolic parameter `Vmax_glucose`:

```
M_glc_D_e
    description: M_D_Glucose_C6H12O6
    max active uptake rate: Monod $Vmax_glucose$ 0.38e-6
    max active export rate: unlimited
    max passive uptake rate: 0
    max passive export rate: 0
    environmental dynamics: initial 0.0556 flux microbial_export
```

click here for source code

Make sure to modify the *external* metabolite version (i.e. `M_glc_D_e` and not `M_glc_D_p` or `M_glc_D_c`). Similarly, in the `species` file modify the cell's `life time` to

```
life time: $life_expectancy$ inhibited_by M_etoh_e $Kinh_ethanol$
```

11.2 Sensitivity analysis

Create a new MCM control script (let's call it `script_02`), and set its contents to the following:

```
# specify the MC model directory
set MCmodelDir "microbial_community_02"

# create a new output directory
```

```
setodnew "simulations_02/run_"

saveScript    # save a backup copy of this script
saveMCmodel   # save a backup copy of the MC model directory

# adjust simulation parameters
set integrationTimeStep 0.01             # integration time step in days
set maxIntegrationTimeStepRefinements 2  # allow up to 2 time step refinements
set maxTimeSeriesSize   100              # record about 100 time points
set maxSimulationTime   4                # simulate four days

# adjust finite-differences scheme
set FD_PrelativeStep  1e-2 # try to approximate derivatives
                           # with this relative finite difference step
set FD_maxRefinements 10   # refine finite differences step
                           # up to 10 times if needed


# SENSITIVITY ANALYSIS RELATED

# only include cell concentrations and metabolite concentrations in sensitivity analysis
set    SA_includeMetaboliteConcentrations
set    SA_includeCellConcentrations
set    SA_includeCellConcentrationsDeadAlive
unset  SA_includeEnvironment
unset  SA_includeMetaboliteExportCommunityWide
unset  SA_includeMetaboliteExportPerCell
unset  SA_includeReactionRatesCommunityWide
unset  SA_includeReactionRatesPerCell
unset  SA_includeCellGrowthRates
unset  SA_includeGeneConcentrations
unset  SA_includeGeneConcentrationsDeadAlive

# disable hierarchical clustering in sensitivity heatmaps
unset SA_clusterParameters
unset SA_clusterVariables

# set a high verbosity to get plenty of progress reports
set SA_verbosity 3

# use a sub-output directory for LSA
changeod LSA

# perform local sensitivity analysis
LSAMCM

# change sub-output directory for GSA
changeod ../GSA

# perform global sensitivity analysis
GSAMCM

quit
```

click here for source code

The above script performs two separate computational tasks: a local sensitivity analysis using `LSAMCM`, followed by a global sensitivity analysis using `GSAMCM`. Because MCM overwrites any existing output files without warnings, we change the output directory between the two commands using `changeod`.

Having adjusted our MC model and our script, we are now ready to perform a sensitivity analysis of the model with respect to the three perturbed parameters, `Vmax_glucose`, `life_expectancy` and `Kinh_ethanol`. Execute the script `script_02` in your command line, e.g. by calling

```
MCM script_02
```

MCM will first perform a simulation of the MC model with the default parameter values, the results of which are saved into the `LSA/run_default` subdirectory. What follows is a series of almost identical simulations of the model, with slightly varied parameter values for the LSA or broadly varied parameter values for the GSA. This is a computationally expensive process; the number of required simulations might range anywhere between 10, 100 or more simulations, depending on the number of perturbed parameters, the *smoothness* of the model dynamics and the control variable `FD_maxRefinements`. In our particular case and on a modern laptop (as of 2014), this might take 10–20 minutes. Intermediate simulation results are not saved unless you set the flag `SA_saveIntermediateSimulations`. Once all sensitivity coefficients have been calculated, MCM creates heatmaps of the LSA and GSA sensitivity matrices (see Fig. 23) and saves detailed reports in `LSA_report.txt` and `GSA_report.txt`. For example, the report written to `LSA_report.txt` should be similar to the following (numbers might differ slightly):

```
Finished local sensitivity analysis after 30 simulations
Final relative finite difference steps:
   Vmax_glucose: finite differences prematurely stopped at relative step 9.765625e-06
   life_expectancy: achieved 0.02 relative finite difference accuracy, at relative step 0.005
   Kinh_ethanol: achieved 0.02 relative finite difference accuracy, at relative step 0.005



# Local normalized sensitivity matrix
                                    Vmax_glucose    life_expectancy   Kinh_ethanol
Concentration_M_glc_D_e             2.47221385932   1.40522728573     1.38477035684
Concentration_M_o2_e                1.47168171122   0.00747004715045  0
Concentration_M_etoh_e              5.23774771504   0.817403632243    0.802015132164
Concentration_M_ac_e                4.96111827703   0.705469564575    0.69186144949
Concentration_M_for_e               5.25214832259   0.817403428565    0.802014931089
Concentration_M_succ_e              5.40166639413   0.817399614998    0.802011575626
CellConcentration_E_coli            10.1041647893   1.71827517753     1.8090542909
CellConcentrationDeadAlive_E_coli   3.62240978094   0.681748713157    0.667723975034



# Did finite-difference quotients estimate partial derivative
# to satisfactory relative accuracy (0.02)?
# If not, then derivative was estimated through
# affine-linear extrapolation of finite differences
                                   Vmax_glucose   life_expectancy   Kinh_ethanol
Concentration_M_glc_D_e            no             yes               yes
Concentration_M_o2_e               no             yes               yes
Concentration_M_etoh_e             no             yes               yes
Concentration_M_ac_e               no             yes               yes
Concentration_M_for_e              no             yes               yes
Concentration_M_succ_e             no             yes               yes
CellConcentration_E_coli           no             yes               yes
CellConcentrationDeadAlive_E_coli  no             yes               yes
```

As part of GSA, MCM also produces the files

- `GSA_responses_to_Vmax_glucose.pdf`,
- `GSA_responses_to_life_expectancy.pdf` and
- `GSA_responses_to_Kinh_ethanol.pdf`,

that show the detailed responses of model predictions to changes in `Vmax_glucose`, `life_expectancy` and `Kinh_ethanol`, respectively (see Fig. 24).

Technical note: Note that finite differentiation might fail to estimate the true partial derivatives with respect to certain parameters, especially if the model's dependency on that parameter is highly non-linear. In that case MCM will try to extrapolate available finite-difference approximations to estimate the true derivatives (as seen in the case of `Vmax_glucose`).

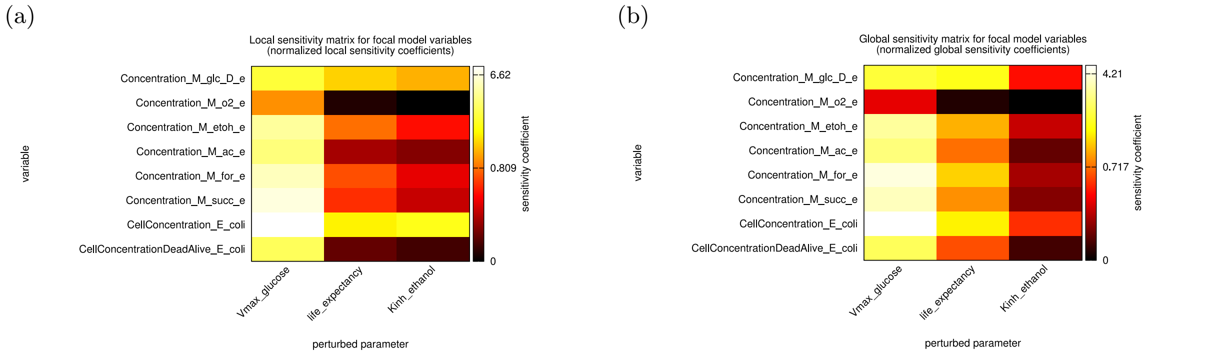

Figure 23: Sensitivity heatmaps generated during (a) local and (b) global sensitivity analysis of the *E. coli* model (see section 11) with respect to three parameters: `Vmax_glucose`, `life_expectancy` and `Kinh_ethanol`. A brighter color corresponds to a greater sensitivity.

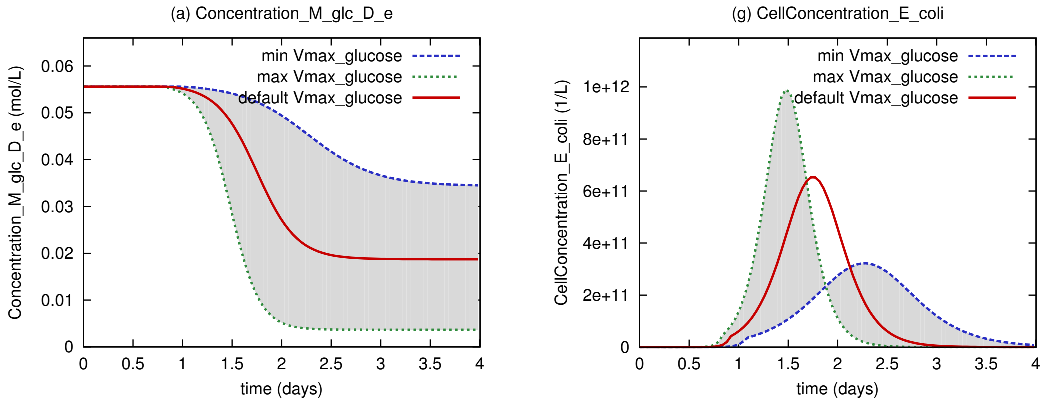

Figure 24: Responses of (a) glucose concentration and (g) cell concentration changes of the parameter `Vmax_glucose`, computed during global sensitivity analysis of the *E. coli* model (see section 11). The red continuous curve corresponds to the default value of `Vmax_glucose`, while the shaded region spans the variation of the model's predictions as `Vmax_glucose` changes from its minimum to maximum value.

11.3 Uncertainty analysis

The sensitivity analysis demonstrated in the previous section allows for a rough evaluation of the influence of parameters on the microbial community. However, neither LSA nor GSA will take into account possible additional information on the probability distribution of uncertain parameters. In this section, we shall use uncertainty analysis (UA) to translate uncertainties of input parameters into uncertainties of model predictions. Let us specify probability distributions for the symbolic parameters by modifying the `parameters` file to the following:

```
file_version: 1.3

Vmax_glucose
    description:  maximum glucose uptake rate
    default:      7.056e-14
    minimum:      5e-14
    maximum:      9e-14
    distribution: triangular
    fixed:        no


life_expectancy
```

```
    description:  expected life duration for starving cells
    default:      19
    minimum:      10
    maximum:      30
    distribution: normal 5 # truncated-normal distribution with sigma=5 days
    fixed:        no


Kinh_ethanol
    description:  ethanol half-inhibition constant
    default:      1.68e-4
    minimum:      1.3e-4
    maximum:      1.9e-4
    distribution: uniform  # uniformly distributed between minimum and maximum
    fixed:        no
```

click here for source code

See section 5.7 for available `distribution` options. In the MC script from section 11.2 replace anything from "`# SENSITIVITY ANALYSIS RELATED`" and below with the following commands:

```
# UNCERTAINTY ANALYSIS RELATED

set UA_MonteCarloTrials  100     # number of random simulations to perform
set UA_colorPalette      parrot  # other options include `heat', `grey', `rainbow'..
set UA_maxCentile        0.95

# choose which observables to show
# note that only focal observables are considered
unset  UA_includeEnvironment                # do not include environmental variables
set    UA_includeMetaboliteConcentrations
set    UA_includeMetaboliteExportCommunityWide
set    UA_includeMetaboliteExportPerCell
set    UA_includeReactionRatesCommunityWide
set    UA_includeReactionRatesPerCell
unset  UA_includeCellConcentrations              # do not include cell concentrations
unset  UA_includeCellGrowthRates            # nor cell growth rates
unset  UA_includeCellConcentrationsDeadAlive
unset  UA_includeGeneConcentrations
unset  UA_includeGeneConcentrationsDeadAlive

# randomly initialize random number generator
seed

# modify output subdirectory
changeod "UA"

# perform UA
UAMCM

quit
```

click here for source code

Executing the new script will result in about 100 simulations (Monte Carlo trials) with parameter values drawn randomly from the distributions specified in the `parameters` file. Upon completion, the sample distributions of model predictions will be plotted into the file `UA/UA.pdf` (an excerpt of which is shown in Fig. 25).

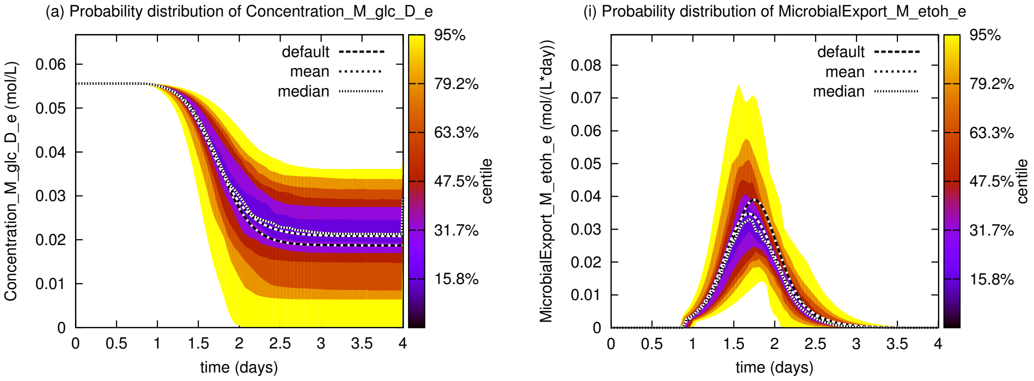

Figure 25: Probability distributions (percentiles centered around medians) of (a) glucose concentration and (i) ethanol export rates estimated through uncertainty analysis of the *E. coli* model (section 11.3), when the three parameters `Vmax_glucose`, `life_expectancy` and `Kinh_ethanol` are chosen randomly.

12 Example 3 - Comparing a two-species model to data

In this example we will examine a two-species model of the nitrifying bacteria *Nitrosomonas* spp. and *Nitrobacter* spp.. The main initial substrate will be ammonium, leading to the chemoautotrophic aerobic growth of *Nitrosomonas* and the production of nitrite as a by-product. The accumulation of nitrite triggers the delayed growth of *Nitrobacter*, which oxidizes the nitrite to nitrate. We will be using a combination of the core (i.e. energy metabolism) cell-metabolic models published by Poughon et al. [63], Starkenburg et al. [85] and Perez-Garcia et al. [60] [18], comprising 24 reactions and 39 metabolites. We will be comparing the predictions of our model to experimental time series data (see section 7), measured by de Boer and Laanbroek [5] in an ammonium-fed mixed *Nitrosospira*[19] and *Nitrobacter* culture. The example will also demonstrate sensitivity analysis of the log-likelihoods (see section 8.2) and the use of a custom environmental variable (pH) defined through a time series measured during the same experiment (see section 5.1).

12.1 Configuring the MC model

The example's MC model and data files should be included with this document. If not, they may be obtained at MCM's official website. Observe that the `parameters` file defines four symbolic parameters, `Vmax_NH3`, `Vmax_NO2`, `init_Ns_concentration` and `init_Nb_concentration`, i.e. the maximum per-cell ammonia and nitrite uptake rates as well as the initial cell concentrations for *Nitrosomonas* and *Nitrobacter*, respectively [72, 5]:

```
file_version: 1.3 # do not remove, move or change this line

Vmax_NH3
    description:  maximum active ammonium/ammonia uptake rate
    default:      1.2342e-13
    minimum:      1e-14
    maximum:      1e-11
    fixed:        no

Vmax_NO2
    description:  maximum active nitrite uptake rate
```

[18]More precisely, the biomass synthesis functions of both species are taken from [60], the energy metabolism of *Nitrosomonas* is taken from [60] and the energy metabolism of *Nitrobacter* is taken from [63]. In addition, an assimilatory nitrite reduction to ammonium reaction, needed for biomass synthesis, was added to *Nitrobacter* [85].

[19]A betaproteobacterium in the same order as *Nitrosomonas*.

```
    default:      3.257e-13
    minimum:      1e-13
    maximum:      1e-10
    fixed:        no

init_Ns_concentration
    description:  initial Nitrosomonas cell concentration
    default:      1e7
    minimum:      1e5
    maximum:      1e9
    fixed:        no

init_Nb_concentration
    description:  initial Nitrobacter cell concentration
    default:      1e7
    minimum:      1e5
    maximum:      1e9
    fixed:        no
```

click here for source code

All parameters are specified as non-`fixed`, because we will be fitting them latter on. Also observe that the `environment` file defines `pH` as an environmental parameter, the temporal profile of which is interpolated from measured time series included in the file `data_03/pH`:

```
pH
  description: pH measured during experiment
  dynamics: value interpolate "../data_03/pH"
  units:
```

Observe that the data file path is given relative to the MC model directory. The `pH` values are required to convert between concentrations of the tree metabolites `NH3`, `NH4` and `NH3_NH4` (ammonia and ammonium together). The three concentrations are defined to be at acid-base dissociation equilibrium, with an acid dissociation constant of 5.62×10^{-10} mol/L. The corresponding entries in the `metabolites` file thus look as follows:

```
..
NH3_NH4
    description: ammonia+ammonium
    max active uptake rate:  custom $Vmax_NH3$ * NH3/(NH3 + 2.6e-5)
    max active export rate:  unlimited
    max passive uptake rate: 0
    max passive export rate: 0
    environmental dynamics:  initial 0.916e-3 flux microbial_export

NH3
    description: ammonia
    max passive uptake rate: 0
    max passive export rate: 0
    environmental dynamics:  base_of_acid_plus_base NH3_NH4 5.62e-10

NH4
    description: ammonium
    max passive uptake rate: 0
    max passive export rate: 0
    environmental dynamics:  acid_of_acid_plus_base NH3_NH4 5.62e-10
..
```

Note that `NH4` and `NH3` are not directly taken up or exported by cells (see the `species` file). In fact, `Nitrosomonas` is assumed to take up ammonium from the formal `NH3_NH4` pool, changes of which are reflected in the `NH3` and `NH4` concentrations. The reason of this setup is that `NH3` is at dissociation equilibrium with `NH4`. The uptake kinetics of `NH3_NH4` are of Monod type depending on ammonia concentration (`NH3`), in line with suggestions by Suzuki et al. [89] that the undissociated form of ammonia may be the actual substrate for oxidation. Similar configurations are used for the `NO2`/`HNO2` pair and the `NO3`/`HNO3`

pair, e.g.

```
..
HNO2_NO2
    description: nitrite + nitrous acid
    max active uptake rate:  Monod $Vmax_NO2$ 2.29e-4
    max active export rate:  unlimited
    max passive uptake rate: 0
    max passive export rate: 0
    environmental dynamics:  initial 0.0196e-3 flux microbial_export

NO2
    description: nitrite
    max passive uptake rate: 0
    max passive export rate: 0
    environmental dynamics:  base_of_acid_plus_base HNO2_NO2 7.1e-4
..
```

Finally, observe that in the `species` file the `initial concentration` for the two cell species is set to the two corresponding symbolic parameters:

```
Nitrosomonas
    initial concentration: $init_Ns_concentration$
    life time:       infinite
    ..

Nitrobacter
    initial concentration: $init_Nb_concentration$
    life time:       infinite
    ..
```

We do not include cell death in the model because we are mainly interested in the growth phase of the two populations.

12.2 Running a single simulation using default parameters

Prepare an MCM script (let's call it `script_03`) similar to the following:

```
# specify the MC model directory
set MCmodelDir "microbial_community_03"

# specify data directory
set MCdataDir "data_03"

# create a new output directory
setodnew "simulations_03/run_"

saveScript    # save a backup copy of this script
saveMCmodel   # save a backup copy of the MC model directory
saveData      # save a backup copy of the data directory

# enable parallel (multithreaded) computation
set parallel

# adjust simulation parameters
set integrationTimeStep 0.01  # integration time step in days
set maxTimeSeriesSize   100   # record about 100 time points
set maxSimulationTime   23    # simulate 23 days

# statistical model for metabolite concentrations
# (the only type of data available)
```

```
set errorModel_MetaboliteConcentrations logNormal

# plot metabolic activity heatmaps every 5 days
# but disable hierarchical clustering
set    plotMetabolicActivityHeatmaps start 0 step 5
unset  metabolicHeatmaps_clusterMetabolites
unset  metabolicHeatmaps_clusterReactions
unset  metabolicHeatmaps_clusterSpecies

# also plot metabolic potential heatmap
set    plotMetabolicPotentialHeatmap

# also plot a diagram of metabolic fluxes on day 1
set    plotMetabolicFluxDiagrams at 1

# run a simulation of the default model in a sub-output directory
changeod "default_simulation"
runMCM

openod
quit
```

click here for source code

Observe that the control variables MCmodelDir and MCdataDir are used to indicate the MC model directory and the directory containing any data for comparison, respectively. Also note that we only run the simulation for 23 days because we lack measurements for pH beyond that point. Execute the script in MCM, e.g. by calling

```
MCM script_03
```

This will perform a single simulation of the model using default parameter values, writing all output to the sub-output directory `default_simulation`. Fig. 26 shows the cell concentrations predicted by the simulation. Fig. 27 shows the metabolic potential (available reactions) and metabolic activity (reaction rates) heatmaps for each species on day 10. Similarly, Fig. 28 shows the relevant metabolites and metabolite uptake/export rates for each species on day 10. Fig. 29 shows a chord diagram of current metabolic fluxes on day 1.

Note: At this point you should make a backup of the model and script, as we will be reverting to it in subsequent examples.

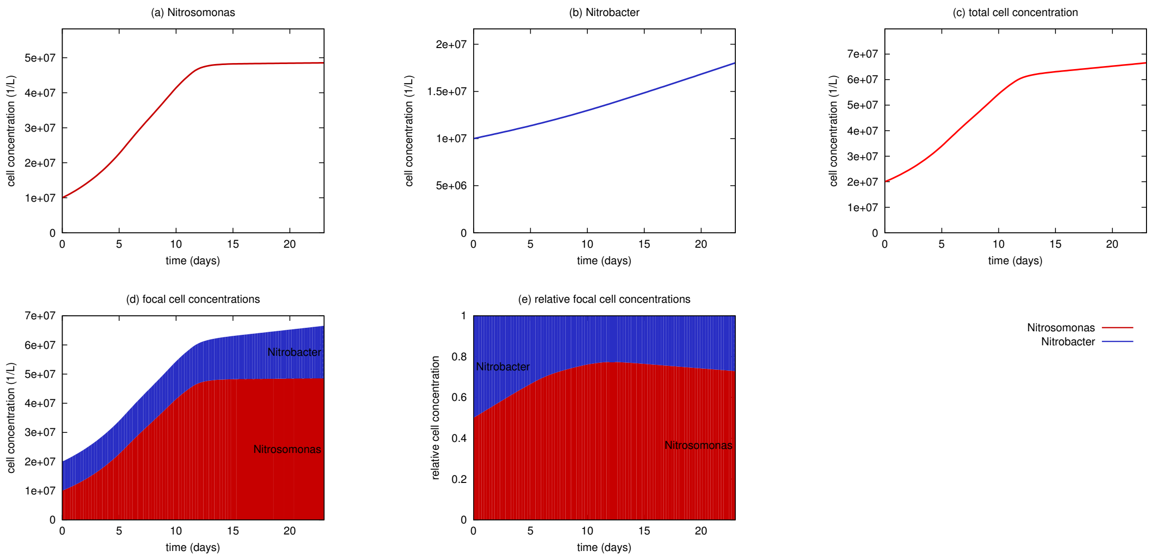

Figure 26: Cell concentrations for the *Nitrosomonas* and *Nitrobacter* nitrifiers, predicted by the MC model described in section 12. *Nitrosomonas*, an ammonia oxidizer, initially grows but reaches saturation upon depletion of ammonium. *Nitrobacter* grows on nitrite, a by-product of ammonium oxidation.

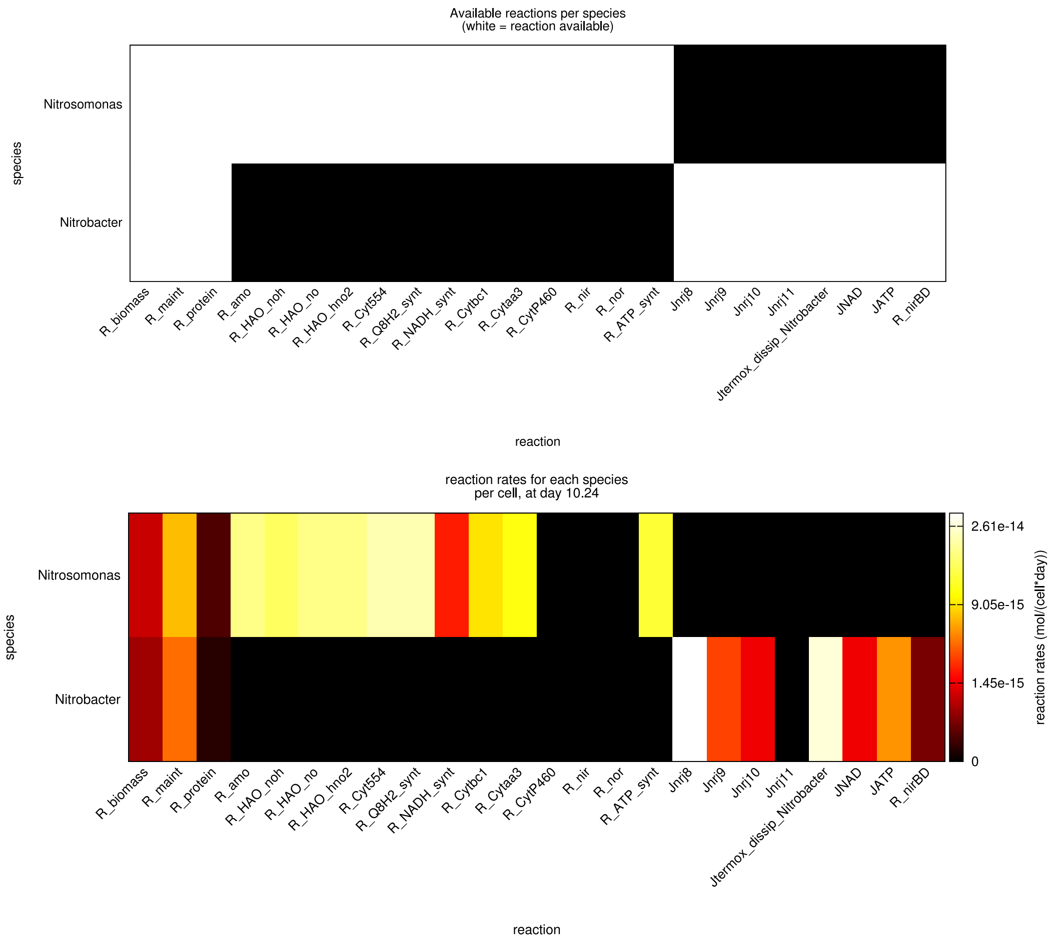

Figure 27: Metabolic potential (top) and metabolic activity (bottom) heatmaps showing available reactions and predicted reaction rates per species, respectively, at day 10 of the simulation described in section 12.2.

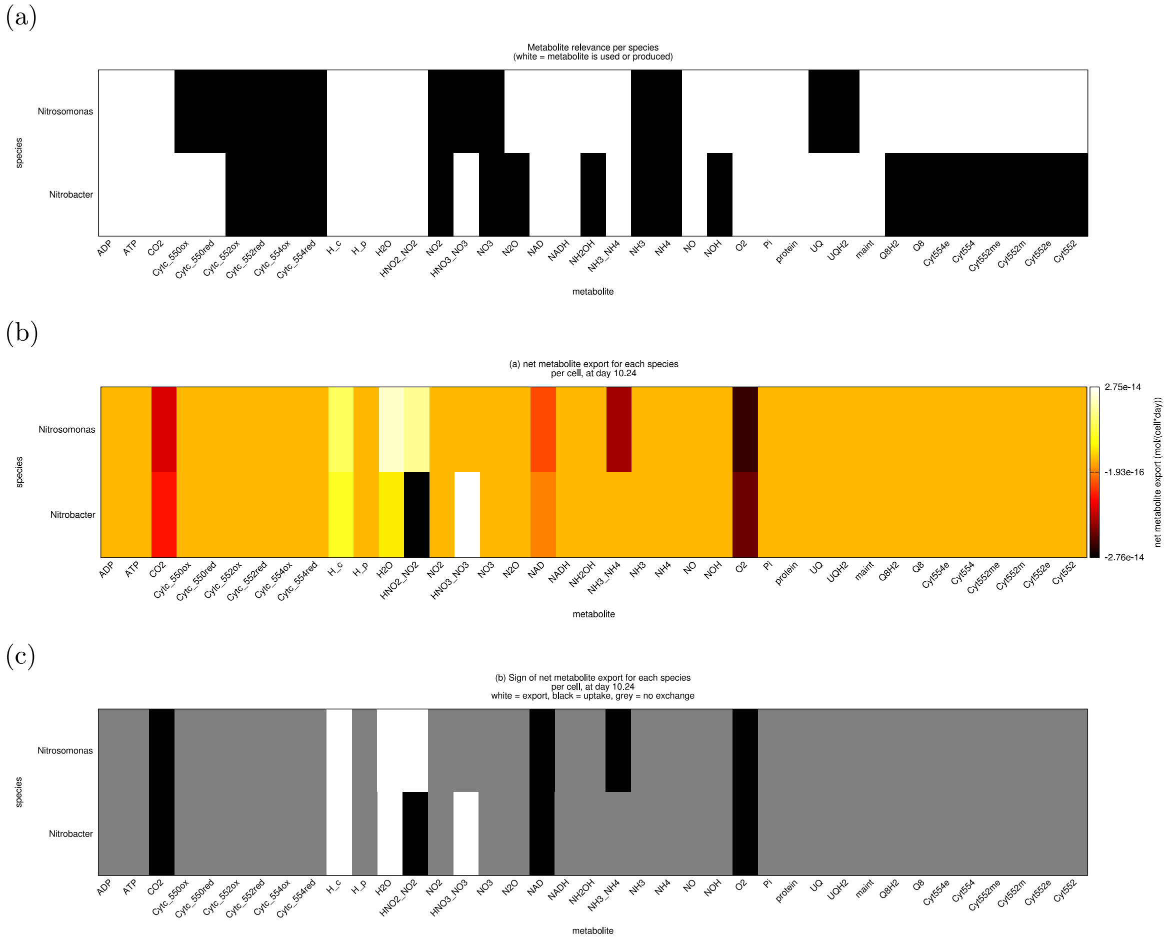

Figure 28: (a) Relevant metabolites per species (metabolic potential heatmap), (b) predicted net uptake rates per cell (metabolic activity heatmap) and (c) sign of (b), at day 10 of the simulation described in section 12.2. Observe that `HNO2_NO2` is exported by `Nitrosomonas` and taken up by `Nitrobacter`.

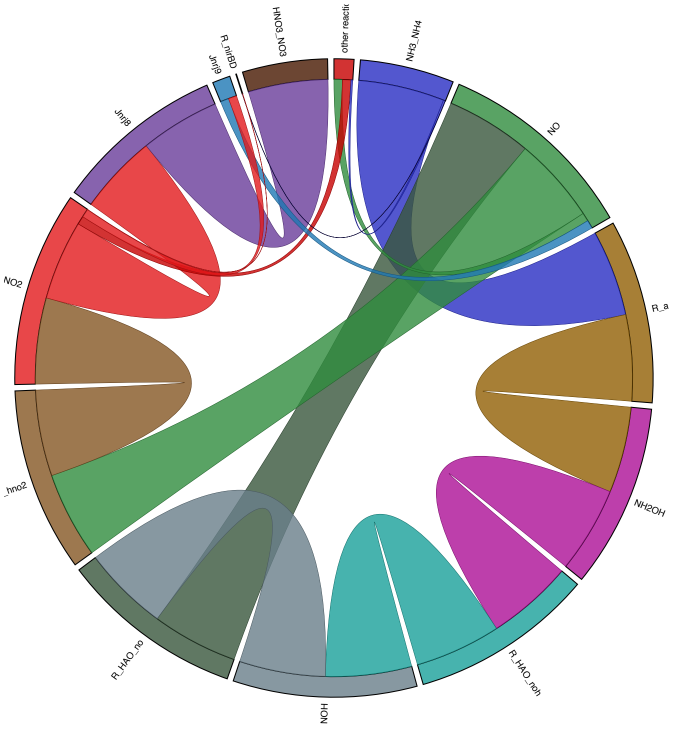

Figure 29: Chord diagram of community-wide metabolic fluxes between reactions and metabolites on day 1. Generated by simulation of the nitrifier community described in section 12.2. Chords connect reactants to reactions and reactions to products. Chords are colored according to their origin. Chord widths are proportional to flux rates. Excerpt from the output file `default_simulation/metabolic_flux_diagrams/current_at_time_1.html`.

12.3 Comparing the simulation to data

As part of the simulation described in the previous section, MCM should automatically detect two data files, `NH4` and `NO3`, located in `data_03/MetaboliteConcentrations` and containing measured time series for the `NH4` and `NO3` concentrations, respectively. At the end of the simulation, the predicted `NH4` and `NO3` concentrations are compared to the two provided time series. The log-likelihood is calculated for each compared quantity as well as the entire model, using the log-normal error model. All relevant output is written to the sub-output directory `default_simulation/comparison_to_data`. Fig. 30 shows the summarizing plot created by MCM. Note that with the default parameter values the model compares very poorly to the data.

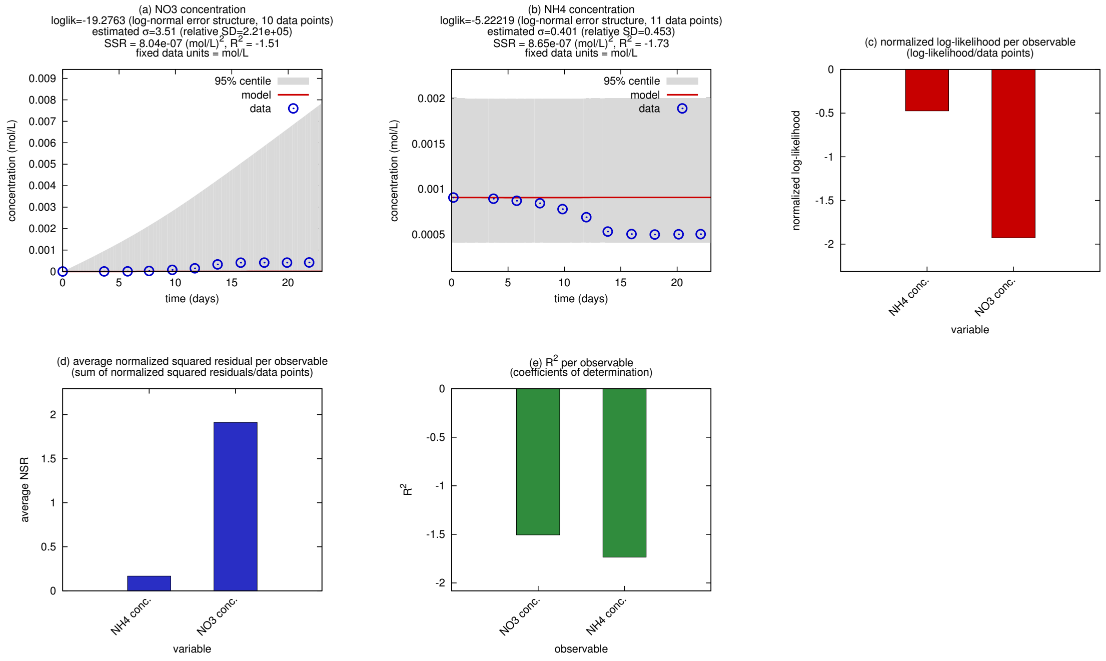

Figure 30: Comparison of predictions with time series data for the (a) NO3 and (b) NH4 concentrations, following a simulation of the nitrifier model described in section 12. Observe the poor comparison of the model predictions to the data. Figure (c) summarizes the normalized log-likelihoods and figure (d) summarizes the average normalized squared residuals for the two quantities, showing that NH4 has a better (but still bad) fit than NO3.

12.4 Local sensitivity analysis of the log-likelihoods

Having observed the poor comparison of the model predictions to the experimental data (Fig. 30), let us perform a local sensitivity analysis (LSA) of the log-likelihoods (see section 8.2) with respect to our symbolic parameters, `Vmax_NH3`, `Vmax_NO2`, `init_Ns_concentration` and `init_Nb_concentration`. This might give us clues about the direction towards which we should adjust these parameters, in order to achieve a better fit. Let us modify the script from section 12.2 by inserting the following commands right after the `runMCM` command:

```
# set a high verbosity to print plenty of information
set   SA_LL_verbosity 3

# change to a different sub-output directory
changeod ../LSA_LL

# perform LSA of the log-likelihoods
LSAMCM_LL
```

click here for source code

Execute the script as before. The sensitivity analysis invoked by the script involves several similar simulations of the model with slightly varied parameters. Once the partial derivatives of the log-likelihoods have been estimated, MCM creates a heatmap of the sensitivity matrix (`LSA_LL_heatmap.pdf`, shown in Fig. 31). MCM also writes a detailed report to `LSA_LL_report.txt`, the last bit of which is exemplified below (actual numbers may vary slightly):

```
Finished local sensitivity analysis of log-likelihood after 16 simulations
Final relative finite difference steps:
   Vmax_NH3: achieved 0.02 relative finite difference accuracy,
             at relative step 0.0005
   Vmax_NO2: achieved 0.02 relative finite difference accuracy,
             at relative step 0.0005
```

```
   init_Ns_concentration: achieved 0.02 relative finite difference accuracy,
                          at relative step 0.0005
   init_Nb_concentration: achieved 0.02 relative finite difference accuracy,
                          at relative step 0.0005


# Local LL sensitivity matrix
            Vmax_NH3        Vmax_NO2       init_Ns_concentration     init_Nb_concentration
NO3 conc.   0.69560092561   2.89569645699  0.383781010527            2.39858845357
NH4 conc.   0.254403660449  0              0.128789475738            0
overall     0.950004586059  2.89569645699  0.512570486265            2.39858845357


# Local NLL sensitivity matrix
            Vmax_NH3         Vmax_NO2        init_Ns_concentration     init_Nb_concentration
NO3 conc.   0.0695600925611  0.289569645699  0.0383781010527           0.239858845357
NH4 conc.   0.0231276054954  0               0.011708134158            0
overall     0.045238313622   0.137890307476  0.0244081183935           0.114218497789


# Did finite-difference quotients estimate partial derivative
# to satisfactory relative accuracy (0.02)?
# If not, then derivative was estimated through
# affine-linear extrapolation of finite differences
            Vmax_NH3   Vmax_NO2   init_Ns_concentration   init_Nb_concentration
NO3 conc.   yes        yes        yes                     yes
NH4 conc.   yes        yes        yes                     yes
overall     yes        yes        yes                     yes
```

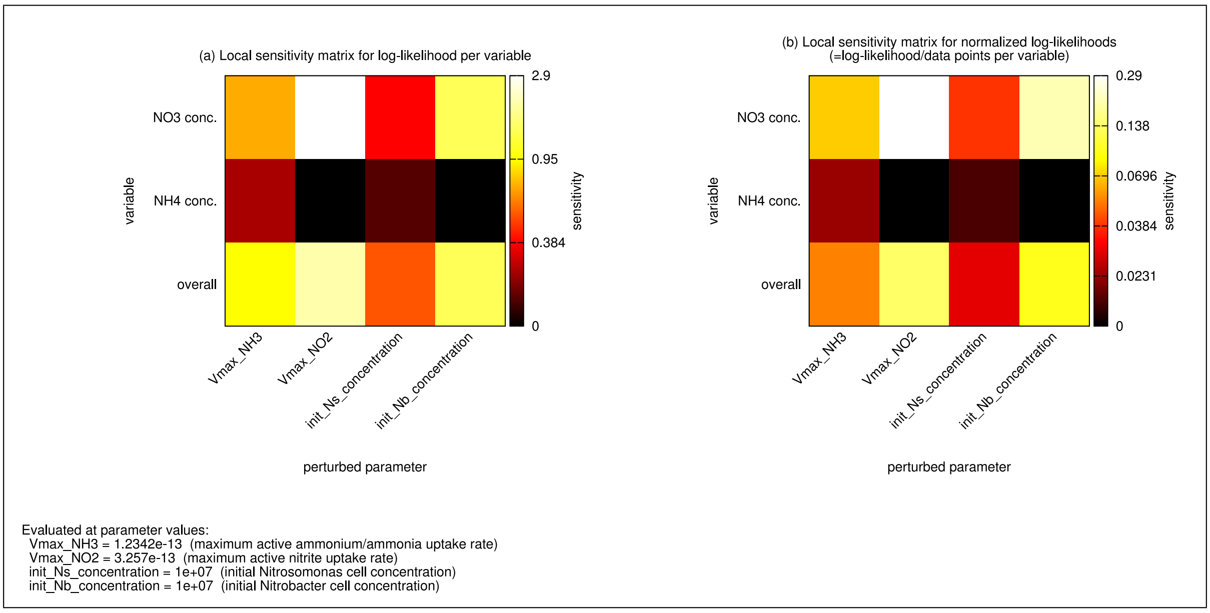

Figure 31: Local sensitivity heatmaps of the log-likelihoods (a) and normalized log-likelihoods (b) for the `NO3` and `NH4` concentrations, with respect to perturbed symbolic parameters (see section 12.4). A positive sensitivity coefficient between a log-likelihood and a parameter means that the prediction would match the data better if the parameter was slightly increased. Observe that **`Vmax_NO2`** and **`init_Nb_concentration`** do not seem to influence the goodness of fit of the `NH4` concentration, in line with expectations (since *Nitrobacter* consumes `NO2`, which is merely the oxidation product of `NH4`).

12.5 Global sensitivity analysis of the log-likelihoods

As suggested by the LSA of the log-likelihoods, we should rather increase all four parameters to achieve a better match with the data. Let's double them and see how that would affect the log-likelihoods using MCM's global sensitivity analysis. In the `parameters` file, set the minimum and maximum parameter values equal to their default and double that, respectively:

```
file_version: 1.3 # do not remove, move or change this line

Vmax_NH3
    description:  maximum active ammonium/ammonia uptake rate
    default:      1.2342e-13
    minimum:      1.2342e-13
    maximum:      2.46e-13
    fixed:        no

Vmax_NO2
    description:  maximum active nitrite uptake rate
    default:      3.257e-13
    minimum:      3.257e-13
    maximum:      6.52e-13
    fixed:        no

init_Ns_concentration
    description:  initial Nitrosomonas cell concentration
    default:      1e7
    minimum:      1e7
    maximum:      2e7
    fixed:        no

init_Nb_concentration
    description:  initial Nitrobacter cell concentration
    default:      1e7
    minimum:      1e7
    maximum:      2e7
    fixed:        no
```

click here for source code

In the previous MCM script from section 12.4, replace all LSA-related entries with the following commands:

```
set SA_LL_saveIntermediateSimulations

# change to another sub-output directory
changeod ../GSA_LL

# perform GSA of the log-likelihoods
GSAMCM_LL
```

click here for source code

Execute the new script as before. The script will invoke a global sensitivity analysis of the log-likelihoods, by testing the effects of choosing each of the parameters at its minimum and maximum value. Observe that we set the flag `SA_LL_saveIntermediateSimulations` because we're interested in the full simulation outputs for all tested parameter values. After a few simulations, MCM saves a detailed report in the file `GSA_LL_report.txt` and creates the sensitivity heatmap shown in Fig. 32. In line with our previous findings, we conclude that an increased value for all four parameters would improve the fit to the data.

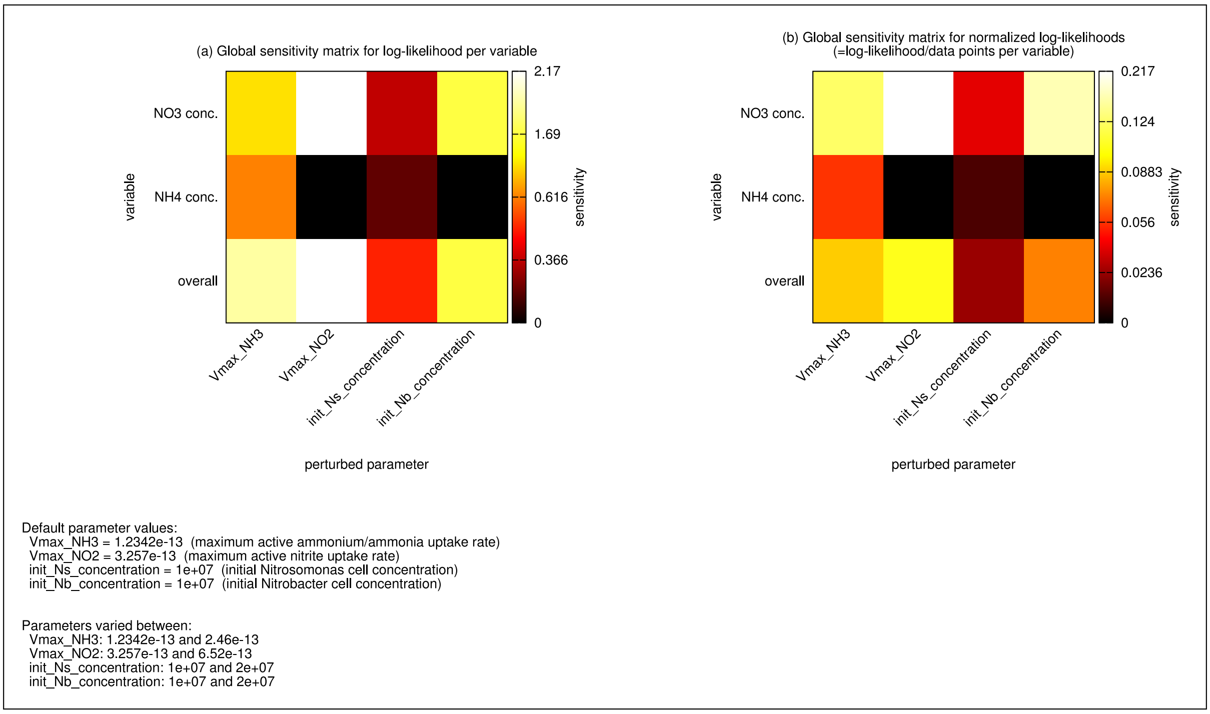

Figure 32: Global sensitivity heatmaps of the log-likelihoods (a) and normalized log-likelihoods (b) for the `NO3` and `NH4` concentrations, with respect to the parameters `Vmax_NH3`, `Vmax_NO2`, `init_Ns_concentration` and `init_Nb_concentration` (see section 12.4). A positive sensitivity coefficient between a log-likelihood and a parameter means that the prediction matches the data better at the parameter's maximum value, rather than its minimum (all other parameters being set to their default).

12.6 Calibrating parameters to data

In section 12.3 we have seen that the default parameter choices (taken from the literature) fail to reproduce the experimental time series data (Fig. 30). Through a local and global sensitivity analysis (sections 12.4 and 12.5) we have convinced ourselves that increasing `Vmax_NH3`, `Vmax_NO2`, `init_Ns_concentration` and `init_Nb_concentration` would improve the goodness of fit, but the *optimal* parameter choice still remains unknown. In this section we will use MCM's fitting routine to estimate the *true* values of our parameters, by maximizing the normalized log-likelihood (NLL) of the model (see section 7.3). In `script_03`, replace all previous sensitivity analysis associated additions with the following commands:

```
# change to a different sub-output directory
changeod ../fitting

# run fitting routine
fitMCM
```

click here for source code

Don't forget to revert the `parameters` file to its original content (as given in section 12.1).

Executing the new script will run a simulation with the default parameter values, followed by a series of simulations with iteratively adjusted parameter values. Once an optimal fit has been reached[20] (in terms of a maximized NLL), MCM performs a final simulation with the fitted parameters and compares it to the data, writing all output to the sub-output directory `fitting/run_final`. MCM will also plot the log-likelihoods encountered during the fitting process, allowing an evaluation of the convergence (Fig. 33).

[20]This can take anywhere from 10 to 1000 or more simulations, depending on the model and default parameter values. Fitting might also prematurely halt if the maximum number of allowed simulations or fitting time has been reached (set using the control variables `fit_maxSimulations` and `fit_maxTime`), or even due to other errors.

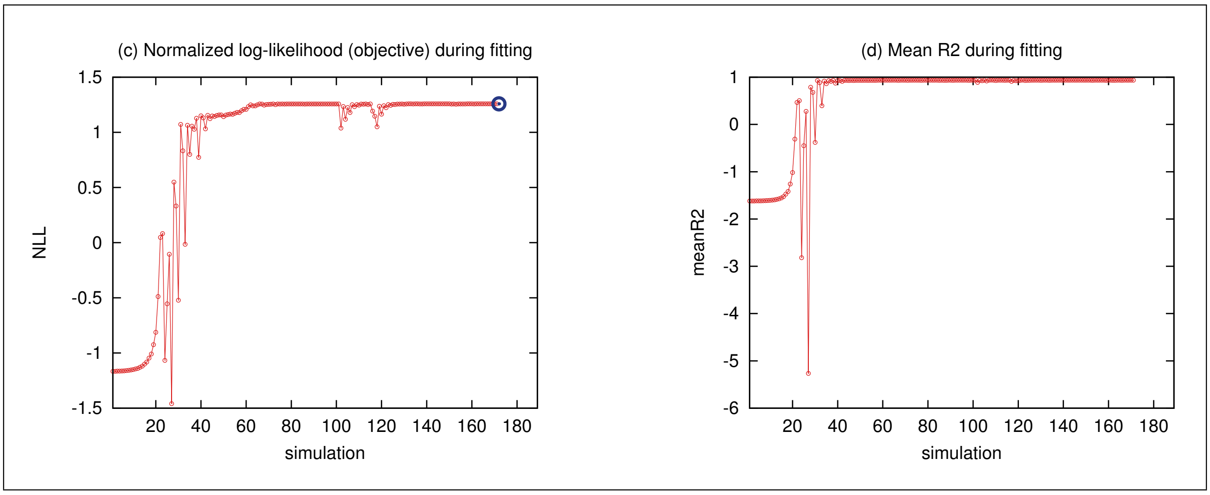

Figure 33: Plot of (a) normalized log-likelihoods and (b) mean coefficients of determination encountered during fitting of the model described in section 12.6. Excerpts from the output file `fit_progress.pdf`.

MCM then tries to estimate 95% confidence intervals (and in fact the entire covariance matrix) for the fitted parameters, a process which involves several additional simulations. The estimated covariance matrix of the fitted parameters is saved as a heatmap (see Fig. 34) in the file `covariance_matrix.pdf`.

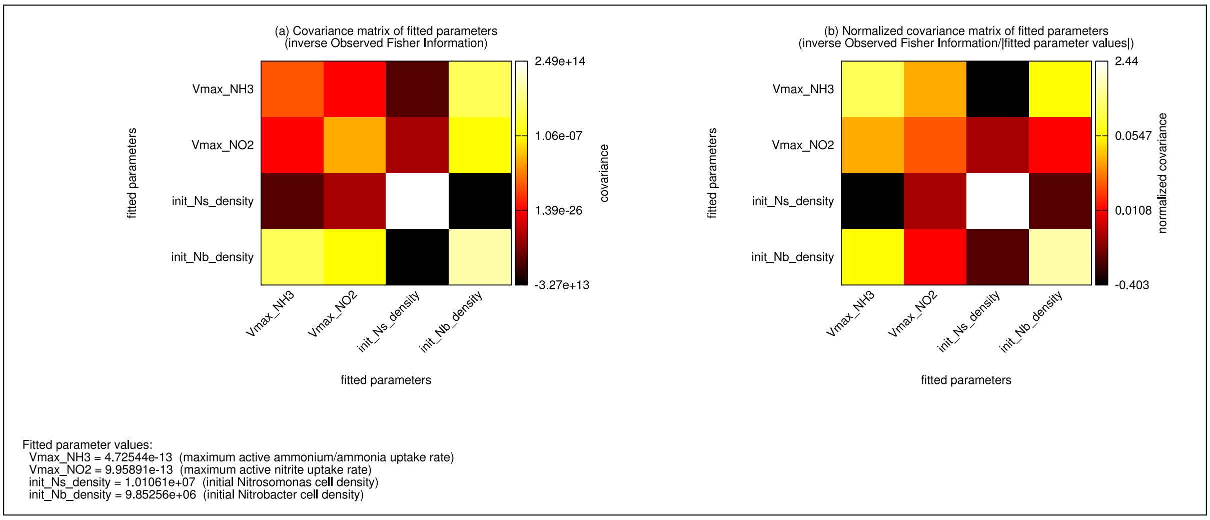

Figure 34: (a) Estimated covariance matrix ($\mathrm{Cov}(p_i, p_j)$) for the fitted parameters, `Vmax_NH3` and `Vmax_NO2`, for the nitrifier model considered in section 12.6. (b) Covariance matrix, with each entry normalized by the fitted parameter values ($\mathrm{Cov}(p_i, p_j)/\,|p_i \cdot p_j|$). Excerpts from the output file `covariance_matrix.pdf`.

The complete fit report is written to the file `fit_report.txt`. The report includes the fitted parameter values and (if available) their estimated confidence intervals, and should look similar to the following:

```
..
Fitting summary:

Fitting completed after 172 simulations: max objective reached within tolerance
  Maximum normalized log-likelihood (MNLL) = 1.258 (= log-likelihood/21 data points)
  Fitted parameter values:
    Vmax_NH3 = 4.72544e-13 (maximum active ammonium/ammonia uptake rate)
    Vmax_NO2 = 9.95891e-13 (maximum active nitrite uptake rate)
    init_Ns_concentration = 1.01061e+07 (initial Nitrosomonas cell concentration)
    init_Nb_concentration = 9.85256e+06 (initial Nitrobacter cell concentration)
```

```
  95% confidence intervals for fitted parameters
  estimated from inverse observed Fisher information:
    Vmax_NH3 = 4.72544e-13 +/- 2.391e-13 (standard error = 1.22e-13 = 25.81 %)
    Vmax_NO2 = 9.95891e-13 +/- 2.889e-13 (standard error = 1.474e-13 = 14.8 %)
    init_Ns_density = 1.01061e+07 +/- 3.096e+07 (standard error = 1.579e+07 = 156.3 %)
    init_Nb_density = 9.85256e+06 +/- 5.15e+06 (standard error = 2.628e+06 = 26.67 %)

Finished after 292 simulations
```

MCM will also create a `parameters` file with the fitted values (called `parameters_fitted`), which can be used directly in the original MC model. The comparison of the fitted model to the data is shown in Fig. 35. Observe the radical improvement of the goodness of fit.

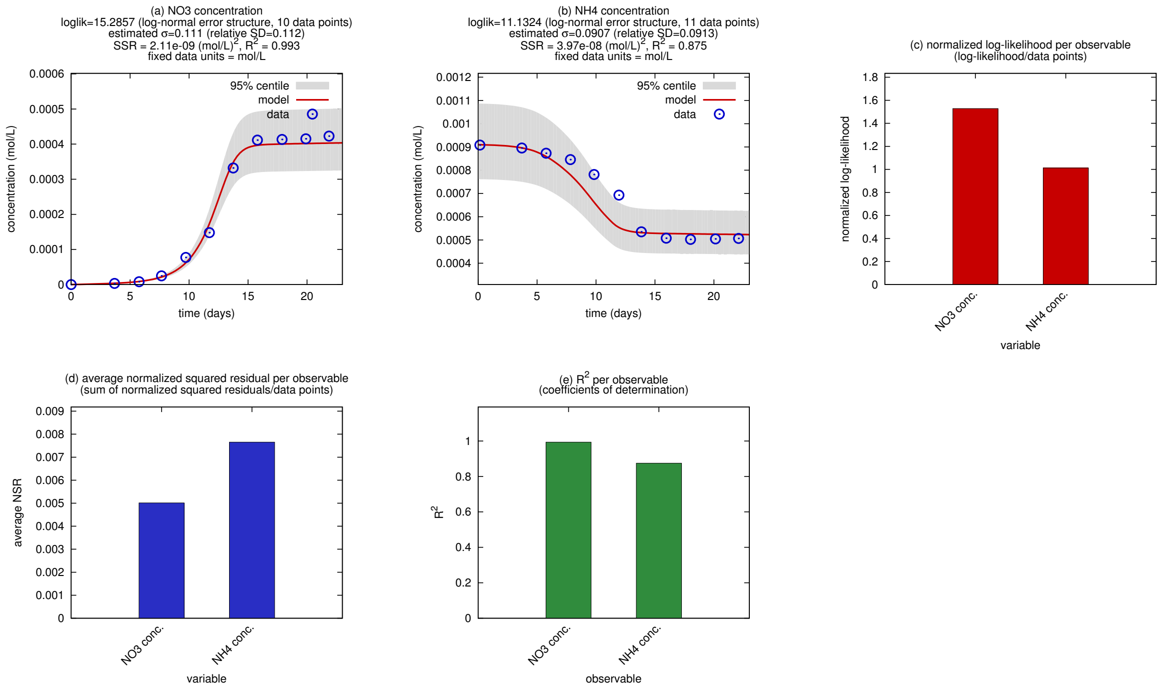

Figure 35: Comparison of predictions with time series data for the (a) NO3 and (b) NH4 concentrations, after a simulation of the fitted nitrifier model considered in section 12.6. Observe the much better reproduction of the data, when compared to the non-fitted model (Fig. 30). Figures (c), (d) and (e) summarize the normalized log-likelihoods, the average normalized squared residuals and the coefficients of determination for the two quantities, respectively.

12.7 Calibrating measurement units

As described in section 7, MCM supports the estimation of data unit scalings in the case that data is given in arbitrary (unknown) linear units. To illustrate this possibility, suppose that the NH4 measurements have unknown units, as might be the case with non-calibrated assays.

To start, revert back to the original model and script from section 12.2. Next, lets tell MCM not to interpret the NH4 data *absolutely* but only *relatively*, in order to estimate the correct scaling: Change the name of the data file NH4 (located in `data_03/MetaboliteConcentrations`) to "`NH4 [au]`" (make sure to include the space). A single simulation results in an estimation of the unknown measurement units for NH4 (Fig. 36).

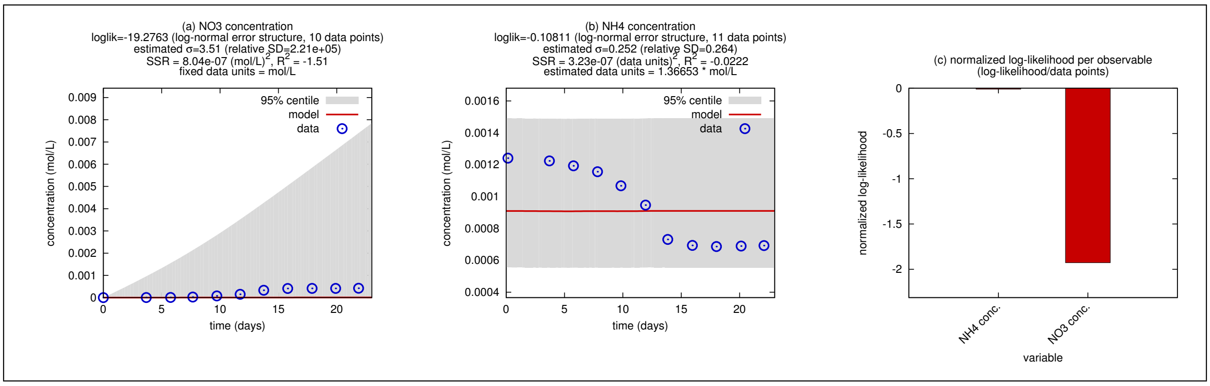

Figure 36: Comparison of predictions with time series data for the (a) `NO3` and (b) `NH4` concentrations, using the original (non-fitted) nitrifier model considered in section 12.7. Because the `NH4` data was labelled as having "arbitrary units", MCM attempts to estimate the correct unit conversion and rescales the plotted data accordingly to match the model units (mol/L in this case).

Let's go one step further, and fit our parameters to the available data (one of them faked of having unknown units). Similarly to section 12.6, simply insert the following lines into the control script after the `runMCM` command:

```
changeod ../fitting
fitMCM
```

Executing the modified script initiates MCM's fitting algorithm, which eventually produces the following fitting report:

```
Fitting summary:

Fitting completed after 315 simulations: max objective reached within tolerance
  Maximum normalized log-likelihood (MNLL) = 1.31087 (= log-likelihood/21 data points)
  Fitted parameter values:
    Vmax_NH3 = 4.64195e-13 (maximum active ammonium/ammonia uptake rate)
    Vmax_NO2 = 9.59793e-13 (maximum active nitrite uptake rate)
    init_Ns_concentration = 1.17477e+07 (initial Nitrosomonas cell concentration)
    init_Nb_concentration = 1.00234e+07 (initial Nitrobacter cell concentration)

  0.95% confidence intervals for fitted parameters
  estimated from inverse observed Fisher information:
    Vmax_NH3 = 4.64195e-13 +/- 1.619e-13 (standard error = 8.26e-14 = 66.92 %)
    Vmax_NO2 = 9.59793e-13 +/- 2.384e-13 (standard error = 1.216e-13 = 37.35 %)
    init_Ns_concentration = 1.17477e+07 +/- 2.442e+07 (standard error = 1.246e+07 = 124.6 %)
    init_Nb_concentration = 1.00234e+07 +/- 4.657e+06 (standard error = 2.376e+06 = 23.76 %)

Finished after 435 simulations
```

Observe that the fitted parameter values differ slightly from the ones estimated previously, achieving an improved NLL. This was of course expected: Since there's one additional parameter to calibrate (the `NH4` measurement units), MCM achieves a better fit to the data (Fig. 37).

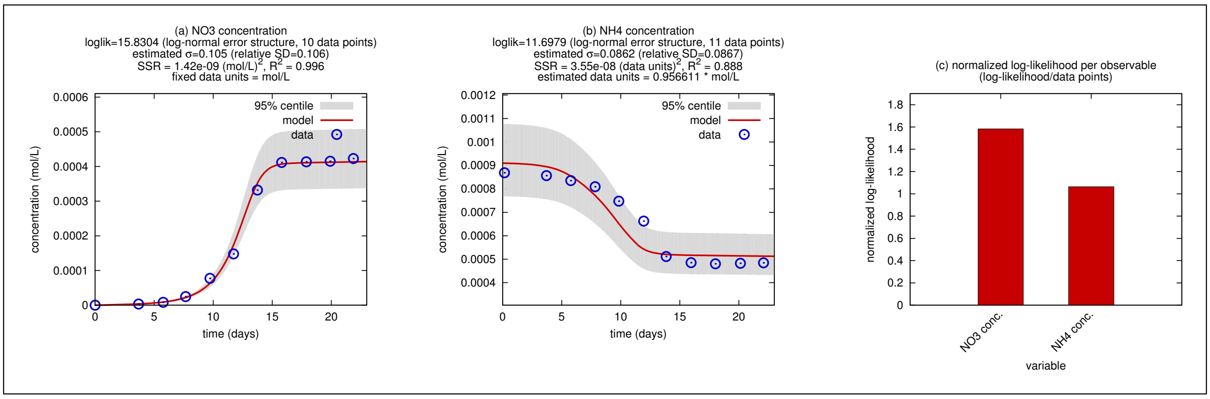

Figure 37: Comparison of predictions with time series data for the (a) NO3 and (b) NH4 concentrations, after a simulation of the fitted nitrifier model considered in section 12.7. Because the NH4 data was labelled as having "arbitrary units", MCM attempts to estimate their correct calibration. Excerpt from the output file `comparison_to_data/comparison.pdf`.

13 MCM output files (reference list)

This section gives an overview of all possible output files produced by MCM. The symbol "*" appended to some file names below stands for any of `.pdf` (or `.svg`), `_data.txt` and `_plot_script.gp`, i.e. a graphical output, the associated raw data and the gnuplot script used to generate the plot (see section 3.5 on plotting).

`plot_script.sh`: Shell script summarizing all created plots. Can be used to re-generate all plots post-simulation. Always created.

`context`: Snapshot of all MCM control variables and flags. Can be used to restore a previous state. Created using the command `saveContext` (see section 4.1).

`FBA_state_initial.txt`: Detailed description of the FBA problems for each cell species at the beginning of any simulation (see section 6). Mainly for troubleshooting.

`FBA_state_final.txt`: Detailed description of the FBA problems for each cell species at the end of any simulation (see section 6). Mainly for troubleshooting.

`parameter_values.txt`: Overview of current parameter values used in a simulation during parameter fitting (see section 7.3) or sensitivity analysis (see section 8).

`cell_biodiversity*`: Predicted time course of cell biodiversity-related measures. Part of any simulation output, e.g. generated by `runMCM` (see section 6).

`gene_biodiversity*`: Predicted time course of gene biodiversity-related measures. Part of any simulation output, e.g. generated by `runMCM` (see section 6).

`cell_concentrations*`: Predicted time course of cell concentrations. Part of any simulation output, e.g. generated by `runMCM` (see section 6).

`cell_concentrations_dead_alive*`: Predicted time course of cell concentrations, including all cells that were ever alive. Part of any simulation output, e.g. generated by `runMCM` (see section 6).

`cell_generations*`: Predicted time course of cell generation for each species, defined as the integrated per-capita birth rate divided by ln(2). Part of any simulation output, e.g. generated by `runMCM` (see section 6).

`cell_growth_rates*`: Predicted time course of cell (per-capita) growth rates. Part of any simulation output, e.g. generated by `runMCM` (see section 6).

`environment*`: Time course of environmental variables defined in the model. Part of any simulation output, e.g. generated by `runMCM` (see sections 5.1 and 6).

observables*: Time course of observables defined in the model. Part of any simulation output, e.g. generated by runMCM (see sections 5.5 and 6).

gene_concentrations*: Predicted time course of gene concentrations (summed over all cells). Part of any simulation output, e.g. generated by runMCM (see section 6).

gene_concentrations_dead_alive*: Predicted time course of gene concentrations (summed over all cells, including all cells that were ever alive). Part of any simulation output, e.g. generated by runMCM (see section 6).

metabolite_concentrations*: Predicted time course of environmental metabolite concentrations. Part of any simulation output, e.g. generated by runMCM (see section 6).

metabolite_net_export_community_wide*: Predicted time course of microbial net metabolite export rates (summed over all cells). Part of any simulation output, e.g. generated by runMCM (see section 6).

metabolite_pure_export_community_wide*: Predicted time course of microbial pure metabolite export rates (summed over all cells). Part of any simulation output, e.g. generated by runMCM (see section 6).

metabolite_pure_uptake_community_wide*: Predicted time course of microbial pure metabolite uptake rates (summed over all cells). Part of any simulation output, e.g. generated by runMCM (see section 6).

metabolite_net_export_per_cell*: Predicted time course of microbial net metabolite export rates (averaged over all cells that use or produce each metabolite). Part of any simulation output, e.g. generated by runMCM (see section 6).

metabolite_uptake_export_rate_limits*: Time course of cellular metabolite exchange rate limits (if variable). Part of any simulation output, e.g. generated by runMCM (see section 6).

reaction_rates_community_wide*: Predicted time course of reaction rates (summed over all cells). Part of any simulation output, e.g. generated by runMCM (see section 6).

metabolite_pure_environmental_production*: Predicted time course of pure environmental (i.e. extracellular) metabolite production rates, summed over all environmental reactions (see section 5.3.5). Part of any simulation output, e.g. generated by runMCM (see section 6).

metabolite_pure_environmental_consumption*: Predicted time course of pure environmental (i.e. extracellular) metabolite consumption rates, summed over all environmental reactions (see section 5.3.5). Part of any simulation output, e.g. generated by runMCM (see section 6).

metabolite_net_environmental_production*: Predicted time course of net environmental (i.e. extracellular) metabolite production rates, summed over all environmental reactions (see section 5.3.5). Part of any simulation output, e.g. generated by runMCM (see section 6).

reaction_rates_per_cell*: Predicted time course of reaction rates, averaged over all cells that perform each reaction. Part of any simulation output, e.g. generated by runMCM (see section 6).

reaction_rates_environmental*: Predicted time course of environmental (i.e. extracellular) reaction rates (see section 5.3.5). Part of any simulation output, e.g. generated by runMCM (see section 6).

reaction_rates_limits*: Time course of cellular reaction rate limits (if variable). Part of any simulation output, e.g. generated by runMCM (see section 6).

reaction_energetics*: Time course of reaction Gibbs free energies and Gibbs free energy release rates (if available). Part of any simulation output, e.g. generated by runMCM (see section 6).

simulation_report.txt: Report produced during a regular simulation (see section 6).

stoichiometry.arff: Community-wide stoichiometric matrix in ARFF format. Part of a regular simulation output generated by runMCM (see section 6).

metabolic_network.sbml: Community-wide metabolic network in SBML file format [37]. Part of a regular simulation output generated by runMCM (see section 6).

theoretical_limits.txt: Estimated maximum cell growth rates based on cell-metabolic network structure. Mainly for troubleshooting. Part of a regular simulation output generated by runMCM (see section 6).

FBA_structure_check.txt: Basic report on the structural consistency (e.g. check of metabolite dead-ends) of the cell-metabolic FBA models. Mainly for troubleshooting. Part of a regular simulation output generated by runMCM (see section 6).

comparison*: Comparison of simulation results and available data. Part of a regular simulation output generated by runMCM (see section 6), or a simulation output generated during fitting (see section 7.3) or sensitivity analysis of the log-likelihood (see section 8.2).

comparison_report.txt: Detailed report on the comparison of simulation results and available data. Part of a regular simulation output generated by runMCM (see section 6), or a simulation output generated during fitting (see section 7.3) or sensitivity analysis of the log-likelihood (see section 8.2).

loglikelihood.txt: Overall log-likelihood of the model when compared to available data, written as a separate file for potentially automated evaluation by 3rd party software. Part of a regular simulation output generated by runMCM (see section 6), or a simulation output generated during fitting (see section 7.3) or sensitivity analysis of the log-likelihood (see section 8.2).

metabolite_fluxes_heatmap*: Heatmap of per-cell net metabolite fluxes for each species, at any arbitrary time during a regular simulation (see section 6).

reaction_rates_heatmap*: Heatmap of per-cell reaction rates for each species, at any arbitrary time during a regular simulation (see section 6).

metabolic_activity.txt: Report on the metabolic activity for each species, at any arbitrary time during a regular simulation (see section 6).

cumulative_metabolite_fluxes_heatmap*: Heatmap of net metabolite fluxes for each species (integrated over time and all cells), at any arbitrary time during a regular simulation (see section 6).

cumulative_reaction_rates_heatmap*: Heatmap of reaction rates for each species (integrated over time and all cells), at any arbitrary time during a regular simulation (see section 6).

cumulative_metabolic_activity.txt: Report on the metabolic activity for each species (integrated over time and all cells), at any arbitrary time during a regular simulation (see section 6).

average_metabolite_fluxes_heatmap*: Heatmap of per-cell net metabolite fluxes for each species (averaged over time), at any arbitrary time during a regular simulation (see section 6).

average_reaction_rates_heatmap*: Heatmap of per-cell reaction rates for each species (averaged over time), at any arbitrary time during a regular simulation (see section 6).

average_metabolic_activity.txt: Report on the metabolic activity for each species (averaged over time), at any arbitrary time during a regular simulation (see section 6).

relevant_metabolites_heatmap*: Heatmap of metabolites that could in principle be used or produced by each species. Part of a regular simulation output generated by runMCM (see section 6).

available_reactions_heatmap*: Heatmap of available reactions (genes) for each species. Part of a regular simulation output generated by runMCM (see section 6).

metabolic_potential.txt: Report on the metabolic potential for each species. Part of a regular simulation output generated by runMCM (see section 6).

LSA_report.txt: Report generated during local sensitivity analysis (see section 8.1).

LSA_heatmap*: Sensitivity heatmap generated during local sensitivity analysis (see section 8.1).

LSA_LL_report.txt: Report generated during local sensitivity analysis of the log-likelihood (see section 8.2).

LSA_LL_heatmap*: Sensitivity heatmap generated during local sensitivity analysis of the log-likelihood (see section 8.2).

GSA_report.txt: Report generated during global sensitivity analysis (see section 8.1).

GSA_heatmap*: Sensitivity heatmap generated during global sensitivity analysis (see section 8.1).

GSA_LL_report.txt: Report generated during global sensitivity analysis of the log-likelihood (see section 8.2).

GSA_LL_heatmap*: Sensitivity heatmap generated during global sensitivity analysis of the log-likelihood (see section 8.2).

UA_report.txt: Report generated during uncertainty analysis (see section 9).

UA*: Plots of probability distributions of model predictions, generated as part of an uncertainty analysis (see section 9).

covariance_matrix*: Covariance matrix of maximum-likelihood estimated (fitted) parameters. Created by fitMCM (see section 7.3).

fit_report.txt: Report created during parameter fitting (see section 7.3).

parameters_fitted: List of final fitted parameters, in a format that can be used in MC models in subsequent simulations (see section 7.3).

fit_progress*: Log-likelihoods encountered during parameter fitting (see section 7.3).

Note that MCM will replace all existing output files without a warning. If you are running multiple simulations, make sure to change output directories between them (see section 4.1 for the relevant commands).

14 FAQ

14.1 Installation and compatibility

14.1.1 I get an error that yacc was not found when compiling

You most likely need to install `yacc` (or the more modern `bison`), which might be required to compile the `lpsolve` library. You can obtain the latest `bison` release here (file `bison-3.0.tar.gz` as of Sept. 7, 2014). Compile and install bison via the commands

```
gunzip -c bison-3.0.tar.gz | tar xopf -
cd bison-3.0
./configure
make
sudo make install
```

click here for source code

14.1.2 I get an error that lex was not found when compiling

You most likely need to install a lexical analyzer generator like `flex` (at least version 2.5.35), which might be required to compile the `lpsolve` library. On Ubuntu or Debian Linux, you can install the latest `flex` release via the commands

```
sudo apt-get update
sudo apt-get install flex
```

Alternatively, you can download the latest `flex` sources from the flex project website (`flex-2.5.39.tar.gz` as of Sept. 7, 2014) and install using

```
gunzip -c flex-2.5.39.tar.gz | tar xopf -
cd flex-2.5.39
./configure
make
```

```
sudo make install
```

click here for source code

You might need to rename the installed `flex` executable to `lex`.

14.1.3 When I compile I get an error that `omp.h` was not found

OpenMP is used to enable parallel computing in MCM. The easiest solution is to compile MCM without OpenMP, i.e. using

```
make openmp=0
```

in the terminal. Also consider switching (upgrading) to a compiler version that supports OpenMP.

Note to Mac OS X users: Recent versions of Apple's XCode install the `clang` compiler (instead of `GCC`), which has limited support for OpenMP and has been found to fail to compile the MCM code even with OpenMP disabled. In this case, you need to compile MCM using the non-XCode `GCC`[21]. Older XCode versions, on the other hand, install Apple's `GCC` version which has a known OpenMP bug. In this case, you can either compile MCM without OpenMP or using the non-Apple `GCC`. In either case, consider using one of the pre-compiled executable binaries available on the MCM website.

14.1.4 I get an error that the `libgomp` library was not found

When compiling MCM with support for parallel computing, the binary is dynamically linked to the OpenMP library `libgomp`. On some systems, that library might not exist, or may not be in the location that MCM expects. The generated error might look similar to

```
dyld: Library not loaded: /opt/local/lib/gcc47/libgomp.1.dylib
```

There are at least 3 solutions to the problem:

- You can compile MCM from the sources without OpenMP, by using

  ```
  make openmp=0
  ```

  in the terminal. Also see section 3.4.
- You can use one of the provided non-parallel binaries.
- You can install a recent GCC version (e.g. GCC 4.7), which comes with the appropriate library.

14.1.5 Heatmap PDF plots appear blurred

This is a known rendering problem of some PDF viewers (like Apple's Preview). There exist at least three solutions:

- Try an alternative PDF viewer (like Acrobat Reader).
- Plot in SVG format instead (use the command `set plotType SVG`).
- Install the command line tool `eps2eps`, which MCM uses to fix blurred heatmaps.

See section 3.5 for more details on plotting.

14.1.6 MCM doesn't generate any plots

You might not have the (proper) gnuplot version installed on your system. If you are getting all the corresponding `_plot_script.gp` and `_data.txt` files, but no plots, this is most likely the case. The current MCM version (1.3) is most compatible with gnuplot 4.6; other gnuplot versions might or might not work. To check if MCM was able to find the correct gnuplot version on your system you can use the command `checkGnuplot` in the MCM command line. To point MCM to the correct gnuplot installation path use the command `setGnuplot`. See section 3.5 for more details.

[21] The latter can be installed using `homebrew` or `MacPorts`.

14.1.7 MCM hangs during a simulation for no apparent reason

This problem can occur on Macs during parallel computation (multithreading) due to an OpenMP bug in Apple's GCC compiler. Try to disable parallel computation (disabled by default) or compile MCM with a different GCC compiler (GCC version ≥ 4.7). Also see the FAQ in section 14.1.3.

14.1.8 Are there any risks associated with using MCM?

MCM is a standalone program that should not interfere with your operating system. That being said, please read the disclaimer in section 15. You should also know that the MCM commands `execute` and `executeinod` (see section 4.1) allow the execution of any shell command with the permissions specified for the user account running MCM. You should therefore restrict the permissions of MCM users appropriately (e.g. don't allow the control of MCM through a public web interface).

14.1.9 Will MCM run on Windows?

Most likely not. We have not tested MCM on any Windows machine, nor did we develop MCM with Windows in mind. Frankly, Windows is not an operating system for high-performance scientific computing.

14.2 Models

14.2.1 How do I include gene regulation?

MCM models all cell metabolism via optimization of a user-provided objective function under flux balance constraints (section 1.3). However, regulation can be emulated by specifying particular reaction rate and metabolite transport rate limits as appropriate functions of metabolite concentrations or environmental variables [15]. For example, to model oxygen inhibition of nitrogen fixation [62], the maximum nitrogen fixation rate might be specified as a decreasing function of oxygen concentration (section 5.3.3). It is also possible to enforce the performance of a reaction, either at a constant rate or as a function of metabolite concentrations or environmental variables (section 5.4.7).

Alternatively, FBA models can be extended to include dynamical internal variables which influence, and are influenced by, a cell's metabolism (see examples in section 5.12). Such non-stationary cell models could accommodate dynamical transcriptional regulatory constraints, that restrict the FBA solution space depending on the abundance of internally produced inhibitors or activators. This approach, known as regulatory FBA (rFBA), is described in detail by [14, 13].

14.2.2 MCM falsely predicts zero cell metabolism

Make sure your FBA models are feasible at the current environmental conditions and metabolite concentrations. The command `runMCM` will produce an output file called `FBA_state_initial.txt`, which lists a detailed description of all FBA models and their optimal solution. If the flag `includeLowLevelLPdetails` is set, MCM includes specifications of the derived linear programming problems in the common `CPLEX-LP` and `lp_solve` LP formats, allowing the independent verification of their feasibility[22]. `runMCM` also tries to detect *dead-end* metabolites in FBA models and prints the results to the file `FBA_structure_check.txt`.

Depending on the control variable `saveCellMetabolicNetworksSBML`, MCM will also save cell-metabolic FBA models as SBML files [37] that can be visualized or analyzed with other tools[23] [94, 80].

Similarly, depending on the control variable `plotMetabolicActivityHeatmaps`, MCM will plot metabolic activity heatmaps, and write a detailed report on the metabolic activity and limiting metabolites and reactions into the file `metabolic_activity.txt` (see section 6).

[22] Note however that many LP solvers will fail on large scale FBA problems [9].

[23] Note that MCM uses "`infinity`" to denote non-existing upper or lower bounds for reaction rates in saved SBML files. Depending on the tool you use to analyze these SBML files, you may need to replace `infinity` with something else (e.g. a very large number).

If the flag checkTheoreticalSystemLimits is set during runMCM, MCM will try to estimate the maximum cell growth rate based on FBA model structure, by setting all metabolite transport and reaction rate limits to their most relaxed value. The results are reported in the file theoretical_limits.txt.

Note that cell-metabolic models often involve thousands of constraints and algebraic coefficients spanning several orders of magnitude. To avoid serious rounding errors, non-trivial (i.e. non-zero and non-infinite) FBA model parameters (e.g. metabolite uptake/export rate limits) of similar physical units must be of similar scales (i.e. less than 8 orders of magnitude apart). If reaction rate or metabolite transport rate limits are not known, do not set them to a *large* number (as is typically done in FBA models to represent infinity). Instead, use the unlimited keyword to denote the absence of a constraint (see sections 5.3.3 and 5.2.1).

14.2.3 My organism should perform reaction X, but FBA does not predict it

FBA predicts metabolism based on optimality principles (in the case of MCM, maximization of biomass production). This assumption is at least questionable for genetically engineered organisms or those exposed to environments that are radically different from the environments that shaped their evolution [83]. Nevertheless, you can *force* an organism to perform a certain reaction as part of its metabolism through a series of modifications to the original metabolic model, as described in section 5.4.7.

14.2.4 What if an FBA problem has no solution or a negative optimum?

If a cell-metabolic FBA model is not solvable or predicts a negative biomass production rate, MCM assumes zero metabolic activity and zero biomass production.

14.2.5 Can I use different error models for different variables?

Yes. Using the control variables listed in section 7.2 you can specify a different error model (e.g. normal or logNormal) for each type of variables such as metabolite concentrations, reaction rates and so on. If you need to use a different error model for similar variable types (e.g. for two different metabolites), you can define an observable for each variable (section 5.5) and explicitly specify the appropriate error model for each observable. For example, to use a normal error model for NH4 and a logNormal error model for NO3, you can define the following two observables:

```
obs_NH4
   value: NH4
   error model: normal
   units: mol/L

obs_NO3
   value: NO3
   error model: logNormal
   units: mol/L
```

Specifying units is optional and only affects axis labeling in graphical output.

14.2.6 How do I keep track of species-specific activity?

To keep track of species- and cell-specific reaction rates and metabolite fluxes during simulations set the control variable output_speciesSpecificMetabolism to either save or saveAndPlot. This will save (and optionally plot) all reaction rates and all metabolite fluxes for all species into the sub-output directory simulation_species_specific. Depending on the model size, the output might use up a significant amount of disk space.

Alternatively, one can keep track of custom observables (section 5.5) focused on individual aspects of a cell's metabolic activity. For example, to keep track of the rate of reaction amo per Nitrosomonas cell one can define the following observable:

```
amo_per_Nitrosomonas_cell
   value: rate_pc.amo.Nitrosomonas
   units: mol/(cell*day)
```

See Table 2 for additional options for species-specific observables. Similarly to other model variables, observables can be compared to data and used for model calibration (section 7). To actually plot an observable's time course, make sure to specify it as focal (section 5.6) and to set the control flag `summary_includeObservables` (section 4.1).

14.3 Control

14.3.1 What's a flag or a control variable and how do I use them?

An MCM flag is a boolean option (which can only be on or off, *set* or *unset*) specified in the MCM command line, which allows the user to toggle between two alternative (typically opposite) behaviors. For example, to toggle between high-contrast and linear-colorspace heatmaps you can either write

```
set highContrastHeatmaps
```

or

```
unset highContrastHeatmaps
```

respectively. A control variable, similarly to flags, controls MCM's subsequent behavior but can have more complicated or specialized values (such as a number, a set of numbers or a file path). For example, `maxSimulationTime` is used to specify the duration of a single simulation, e.g. as

```
set maxSimulationTime 10
```

A more complicated example is `plotMetabolicActivityHeatmaps`, which holds a sequence of times at which MCM is to plot metabolic activity heatmaps during a simulation. Setting the value of this variable requires a special syntax. For example, writing

```
set plotMetabolicActivityHeatmaps start 0 step 2 end 10
```

will assign to the variable the sequence `{0, 2, 4, 6, 8, 10}`. One can always obtain an overview of a variable's purpose and syntax using the `help` command, e.g.

```
help plotMetabolicActivityHeatmaps
```

Consult section 4.2 for an interpretation of syntax overviews.

14.3.2 Can I analyze or vizualize MC models using 3rd party tools?

The short answer is yes. The long answer is that you need to save the MC model into another common file format like `ARFF`, `BIOM` [52], `GEXF` [3] or `SBML` [37]. This can be done as part of a regular simulation, as described in section 6. Note that when exporting MC models into other file formats some information is potentially lost, for example if the target file format does not support the full complexity of MC models.

14.3.3 How can I improve the quality of plots?

Every plot generated by MCM (e.g. `UA.pdf`) is accompanied by the corresponding raw data (`UA_data.txt`), as well as a self-contained gnuplot script (`UA_plot_script.gp`) used to generate the plot. Gnuplot itself is a sophisticated graphing tool capable of producing high quality plots (consult the gnuplot homepage for usage instructions). You can either adjust and re-run the gnuplot script to produce a modified version of the plot, or use any graphing tool of your choice with the raw data. Alternatively, you can choose to plot in SVG file format and directly edit the generated plots in any SVG editor. See section 3.5 for more details on plotting.

14.3.4 Can I change the appearance of heatmaps?

The coloring of heatmaps can be modified through the control variable `heatmapColorPalette` and the flag `highContrastHeatmaps`, e.g. as:

```
set heatmapColorPalette heat  # heatmap coloring scheme
                              # other options include parrot, grey, printableOnGrey, rainbow ..
set highContrastHeatmaps      # emphasize differences in heatmaps (use nonlinear color scale)
```

In heatmaps generated by sensitivity analysis (see section 8) or covariance heatmaps generated during parameter fitting (see section 7.3), the order of parameters and output variables can either be as defined in the MC model or chosen using hierarchical clustering. You can toggle between the two options using the flags SA_clusterVariables, SA_clusterParameters, SA_LL_clusterVariables, SA_LL_clusterParameters and fit_clusterParameters, e.g. as follows:

```
set fit_clusterParameters     # enable hierarchical clustering of parameters
                              # in covariance matrix heatmap

set   SA_clusterParameters    # cluster parameters
unset SA_clusterVariables     # but don't cluster variables in sensitivity analysis heatmaps
```

Similarly, for metabolic potential and metabolic activity heatmaps generated during a regular simulation (see section 6), metabolites, reactions and species can be clustered or shown in their standard order, depending on the flags

- metabolicHeatmaps_clusterMetabolites,
- metabolicHeatmaps_clusterReactions and
- metabolicHeatmaps_clusterSpecies,

respectively. One can also choose between showing all metabolites, reactions and species or just focal ones (see section 5.6 on focals), depending on the flags

- metabolicHeatmaps_onlyFocalMetabolites,
- metabolicHeatmaps_onlyFocalReactions and
- metabolicHeatmaps_onlyFocalSpecies,

respectively. Finally, as with all MCM-generated plots, one can manually adjust heatmaps post-creation as described in section 14.3.3.

14.3.5 Some simulations just hang on one time point for ever

The simplex algorithm used by MCM to solve FBA problems has exponential ($\mathcal{O}(e^n)$) worst case complexity [43], and some FBA problems can take an unusually long time to be solved. This happens rarely, but depends on the particular metabolic stoichiometry of a cell and the constraints currently imposed by the environment. To "jump over" such locks, you can use the control variable FBATimeOut to specify a time threshold (in seconds) beyond which an FBA problem will cancel and return with no solution. At that time point, the metabolism of the cell will be set to zero (i.e. all pathways deactivated).

14.3.6 What format should my time series data files be in?

MCM assumes that all time series data files are in space-delimited two-column (time & value) tabular format. Time points can be in any order and duplicate times are also allowed. In the case of data used for statistical comparisons (see sections 7 and 8.2), multiple values at identical time points are either averaged (if the flag averageAmbiguousData is set) or treated as independent measurements (if averageAmbiguousData is unset). In the case of interpolated time series used to define some model variable (e.g. an external metabolite influx or an environmental variable, see sections 5.2.2 and 5.1), values at duplicate time points are averaged prior to interpolation. Empty lines, excessive whitespace and comments preceded by the "#" symbol are ignored. An example time series data file is shown below:

```
# pH measured during Nitrobacter & Nitrosospira ammonium oxidation experiment
# Taken from de Boer 1989, Fig. 2 (DOI: 10.1007/BF00456098)
# Time(days)  pH
0             7.085
3.462         7.101
```

```
5.425        7.095

# some more data
15.855       4.699
9.5575       6.819
```

Time series data files can include optional `factor` or `transform` directives for modifying data before it is loaded by MCM. `factor` tells MCM to multiply all subsequent time series values with a given numerical factor. `transform` tells MCM to transform all subsequent time series values using a given functional expression of `x`. A `factor` and `transform` directive only applies to the entries below it, and only until another `factor` or `transform` directive is encountered. For example, the file

```
0            7.085
factor       10
3.462        0.7101
factor       100
5.425        0.07095
15.855       0.004699
transform    2*(x+1)
9.5575       2.4095
```

defines the same time series as the previous one. Furthermore, the optional directive `end` tells MCM to ignore any subsequent values. For example, the file

```
0            7.085
3.462        7.101
5.425        7.095
15.855       4.699
9.5575       6.819
end
11.232       5.487
12.311       4.922
```

defines the same time series as the two previous ones.

14.3.7 Can I simulate multiple model variants at once?

Yes. You can use the command runMCM (or any other command) multiple times in the same MCM script file, while modifying your model in between using macros (section 4.3). Make sure to change the output directory for each new simulation, otherwise previous simulation results might be overwritten. For example, let's say we have an MC model containing the following species:

```
Nitrosomonas
    initial concentration: 1e8
    mass:                 3e-13
    life time:            infinite
    reactions:            amo
    active uptakes:       O2, NH4
    active exports:       NO2
```

Apart from an `infinite` life expectancy, we are also interested in a finite life expectancy of 10 days, and we wish to run a separate simulation of each model variant. For that purpose, we use a macro (let's call it `life_time_Ns`) and modify the above species definition to the following:

```
Nitrosomonas
    initial concentration: 1e8
    mass:                 3e-13
    life time:            $life_time_Ns$
    reactions:            amo
    active uptakes:       O2, NH4
    active exports:       NO2
```

We can now define and subsequently modify the macro `life_time_Ns` between simulations from within the MCM script, e.g. as follows:

```
# create a new output directory, whose name includes today's date and time
setod "simulation $now$"

# create output sub-directory for 1st model variant
changeod "run_life_time_infinite"

# define macro
macro life_time_Ns:infinite

# run simulation of 1st model variant
runMCM

# create new output sub-directory for 2nd model variant
changeod "../run_life_time_10_days"

# modify macro
macro life_time_Ns:10

# run simulation of 2nd model variant
runMCM
```

14.3.8 Can I limit the statistical analysis to a specific time interval?

Yes. You can specify a time interval (or a union of multiple intervals) within which to statistically evaluating the model, e.g. when fitting it to data. For this specify the control variable `statisticsInterval`, e.g. as in the examples below:

```
set statisticsInterval never
set statisticsInterval [0:10]              # days 0 to 10
set statisticsInterval [0:10] and [20:30]  # days 0 to 10 and 20 to 30
set statisticsInterval [0:10] and [20:*]   # days 0 to 10 and 20 to infinity
```

`statisticsInterval` also modifies the time range considered by sensitivity analysis (section 8) and time series analysis (section 6).

14.3.9 How do I exactly repeat a stochastic simulation?

MCM initializes its random number generators using the command `seed`. If this command is not called, all stochastic routines of MCM will produce the same outcome every time MCM is re-started. If `seed` is called with a numerical argument (a *seed number*, e.g. `seed 1000`), then MCM's random number generators will be initialized according to the given seed number. Alternatively, if the call to `seed` did not include an explicit seed number, a random seed number is chosen and printed to the command line. Hence, whenever you call `seed` with the same seed number, your subsequent stochastic simulations will remain unchanged, provided that you neither modified the MC model nor MCM's command line options related to model integration (e.g. time steps).

14.4 Citing and contributing

14.4.1 How do I cite MCM in my work?

Please use the information provided in the following BibTeX entry:

```
@article{LoucaMCM2015,
Author = {Stilianos Louca and Michael Doebeli},
Title = {Calibration and analysis of genome-based models for microbial ecology},
Journal = {eLife},
Year = {2015},
```

```
Howpublished = {\url{http://www.zoology.ubc.ca/MCM}}
```

For more complete, up-to-date citation info please consult the MCM website.

14.4.2 How can I thank the creators of MCM?

If you use MCM in any published research, you are kindly asked to cite our work appropriately. Citations are vital to our careers as scientists. Please consult section 14.4.1 for citation details. Beyond that, feel free to recommend MCM to colleagues, or even help improve it (see section 14.4.3).

14.4.3 How can I contribute to MCM's improvement?

If you used MCM for your research, we would love to hear about your experience and any difficulties you encountered. You are invited to send us bug reports, suggestions and feature requests. Contact details can be obtained at MCM's website: `http://www.zoology.ubc.ca/MCM`.

15 Disclaimer and license agreement

MCM is free software: you can redistribute it and/or modify it under the terms of the GNU Lesser General Public License as published by the Free Software Foundation, either version 3 of the License, or (at your option) any later version. MCM is distributed in the hope that it will be useful, but WITHOUT ANY WARRANTY; without even the implied warranty of MERCHANTABILITY or FITNESS FOR A PARTICULAR PURPOSE. See the GNU Lesser General Public License for more details. You should have received a copy of the GNU Lesser General Public License along with MCM. If not, see `http://www.gnu.org/licenses/lgpl.html`.

References

[1] Almaas, E., Kovacs, B., Vicsek, T., Oltvai, Z., Barabási, A.L., 2004. Global organization of metabolic fluxes in the bacterium *Escherichia coli*. Nature 427, 839–843.

[2] Atkins, G.L., Nimmo, I.A., 1980. Current trends in the estimation of Michaelis-Menten parameters. Analytical Biochemistry 104, 1–9.

[3] Bastian, M., Heymann, S., Jacomy, M., 2009. Gephi: An open source software for exploring and manipulating networks. AAAI Publications .

[4] Blazier, A.S., Papin, J.A., 2012. Integration of expression data in genome-scale metabolic network reconstructions. Frontiers in Physiology 3.

[5] de Boer, W., Laanbroek, H., 1989. Ureolytic nitrification at low pH by *Nitrosospira* spec. Archives of Microbiology 152, 178–181.

[6] Canfield, D.E., Thamdrup, B., 2009. Towards a consistent classification scheme for geochemical environments, or, why we wish the term 'suboxic' would go away. Geobiology 7, 385–392.

[7] Cariboni, J., Gatelli, D., Liska, R., Saltelli, A., 2007. The role of sensitivity analysis in ecological modelling. Ecological Modelling 203, 167–182.

[8] Carlin, B., Louis, T., 2011. Bayesian Methods for Data Analysis. Chapman & Hall/CRC Texts in Statistical Science, Taylor & Francis. 3 edition.

[9] Chindelevitch, L., Trigg, J., Regev, A., Berger, B., 2014. An exact arithmetic toolbox for a consistent and reproducible structural analysis of metabolic network models. Nat Commun 5.

[10] Chiu, H.C., Levy, R., Borenstein, E., 2014. Emergent biosynthetic capacity in simple microbial communities. PLoS Computational Biology 10, e1003695.

[11] Christopher Frey, H., Patil, S.R., 2002. Identification and review of sensitivity analysis methods. Risk analysis 22, 553–578.

[12] Cleveland, C., Liptzin, D., 2007. C:N:P stoichiometry in soil: is there a "Redfield ratio" for the microbial biomass? Biogeochemistry 85, 235–252.

[13] Covert, M.W., Palsson, B.Ø., 2002. Transcriptional regulation in constraints-based metabolic models of *Escherichia coli*. Journal of Biological Chemistry 277, 28058–28064.

[14] Covert, M.W., Schilling, C.H., Palsson, B., 2001. Regulation of gene expression in flux balance models of metabolism. Journal of Theoretical Biology 213, 73–88.

[15] Covert, M.W., Xiao, N., Chen, T.J., Karr, J.R., 2008. Integrating metabolic, transcriptional regulatory and signal transduction models in *Escherichia coli*. Bioinformatics 24, 2044–2050.

[16] David, L.A., Materna, A.C., Friedman, J., Campos-Baptista, M.I., Blackburn, M.C., Perrotta, A., Erdman, S.E., Alm, E.J., 2014. Host lifestyle affects human microbiota on daily timescales. Genome Biology 15, R89.

[17] Davidson, R., MacKinnon, J., 2004. Econometric Theory and Methods. Oxford University Press.

[18] Dean, J., 1998. Lange's Handbook of Chemistry. McGraw-Hill Professional. 15 edition.

[19] Dennis, B., Ponciano, J., 2014. Density-dependent state-space model for population-abundance data with unequal time intervals. Ecology 95, 2069–2076.

[20] Duarte, N.C., Herrgård, M.J., Palsson, B.Ø., 2004. Reconstruction and validation of *Saccharomyces cerevisiae* iND750, a fully compartmentalized genome-scale metabolic model. Genome Research 14, 1298–1309.

[21] Edwards, J.S., Ibarra, R.U., Palsson, B.O., 2001. In silico predictions of *Escherichia coli* metabolic capabilities are consistent with experimental data. Nature Biotechnology 19, 125–130.

[22] Emerson, S., Hedges, J., 2008. Chemical Oceanography and the Marine Carbon Cycle. Cambridge University Press, Cambridge, UK.

[23] Fagerbakke, K., Heldal, M., Norland, S., 1996. Content of carbon, nitrogen, oxygen, sulfur and phosphorus in native aquatic and cultured bacteria. Aquatic Microbial Ecology 10, 15–27.

[24] Feist, A.M., Henry, C.S., Reed, J.L., Krummenacker, M., Joyce, A.R., Karp, P.D., Broadbelt, L.J., Hatzimanikatis, V., Palsson, B.Ø., 2007. A genome-scale metabolic reconstruction for *Escherichia coli* K-12 MG1655 that accounts for 1260 ORFs and thermodynamic information. Molecular Systems Biology 3, 121.

[25] Feist, A.M., Palsson, B.O., 2010. The biomass objective function. Current Opinion in Microbiology 13, 344–349.

[26] Freilich, S., Zarecki, R., Eilam, O., Segal, E.S., Henry, C.S., Kupiec, M., Gophna, U., Sharan, R., Ruppin, E., 2011. Competitive and cooperative metabolic interactions in bacterial communities. Nature Communications 2, 589.

[27] Gillespie, D., 1992. Markov Processes: An Introduction for Physical Scientists. Academic Press.

[28] Hansen, H., Koch, C.B., 1998. Reduction of nitrate to ammonium by sulphate green rust: activation energy and reaction mechanism. Clay Minerals 33, 87–101.

[29] Hansen, H.C.B., Koch, C.B., Nancke-Krogh, H., Borggaard, O.K., Sørensen, J., 1996. Abiotic nitrate reduction to ammonium: key role of green rust. Environmental Science & Technology 30, 2053–2056.

[30] Harcombe, W.R., Delaney, N.F., Leiby, N., Klitgord, N., Marx, C.J., 2013. The ability of flux balance analysis to predict evolution of central metabolism scales with the initial distance to the optimum. PLOS Computational Biology 9, e1003091.

[31] Harcombe, W.R., Riehl, W.J., Dukovski, I., Granger, B.R., Betts, A., Lang, A.H., Bonilla, G., Kar, A., Leiby, N., Mehta, P., Marx, C.J., Segrè, D., 2014. Metabolic resource allocation in individual microbes determines ecosystem interactions and spatial dynamics. Cell Reports 7, 1104–1115.

[32] Henry, C.S., DeJongh, M., Best, A.A., Frybarger, P.M., Linsay, B., Stevens, R.L., 2010. High-throughput generation, optimization and analysis of genome-scale metabolic models. Nature Biotechnology 28, 977–982.

[33] Herrgård, M.J., Lee, B.S., Portnoy, V., Palsson, B.Ø., 2006. Integrated analysis of regulatory and metabolic networks reveals novel regulatory mechanisms in *Saccharomyces cerevisiae*. Genome research 16, 627–635.

[34] Hoehler, T.M., Alperin, M.J., Albert, D.B., Martens, C.S., 2001. Apparent minimum free energy requirements for methanogenic archaea and sulfate-reducing bacteria in an anoxic marine sediment. FEMS Microbiology Ecology 38, 33–41.

[35] Hood, R.R., Laws, E.A., Armstrong, R.A., Bates, N.R., Brown, C.W., Carlson, C.A., Chai, F., Doney, S.C., Falkowski, P.G., Feely, R.A., Friedrichs, M.A.M., Landry, M.R., Keith Moore, J., Nelson, D.M., Richardson, T.L., Salihoglu, B., Schartau, M., Toole, D.A., Wiggert, J.D., 2006. Pelagic functional group modeling: Progress, challenges and prospects. Deep Sea Research II: Topical Studies in Oceanography 53, 459–512.

[36] Hoover, S.R., Porges, N., 1952. Assimilation of dairy wastes by activated sludge: II. The equation of synthesis and rate of oxygen utilization. Sewage and Industrial Wastes 24, 306–312.

[37] Hucka, M., Finney, A., Sauro, H.M., Bolouri, H., Doyle, J.C., Kitano, H., , the rest of the SBML Forum:, Arkin, A.P., Bornstein, B.J., Bray, D., Cornish-Bowden, A., Cuellar, A.A., Dronov, S., Gilles, E.D., Ginkel, M., Gor, V., Goryanin, I.I., Hedley, W.J., Hodgman, T.C., Hofmeyr, J.H., Hunter, P.J., Juty, N.S., Kasberger, J.L., Kremling, A., Kummer, U., Le Novère, N., Loew, L.M., Lucio, D., Mendes, P., Minch, E., Mjolsness, E.D., Nakayama, Y., Nelson, M.R., Nielsen, P.F., Sakurada, T., Schaff, J.C., Shapiro, B.E., Shimizu, T.S., Spence, H.D., Stelling, J., Takahashi, K., Tomita, M., Wagner, J., Wang, J., 2003. The systems biology markup language (sbml): a medium for representation and exchange of biochemical network models. Bioinformatics 19, 524–531.

[38] Ibarra, R.U., Edwards, J.S., Palsson, B.O., 2002. *Escherichia coli* K-12 undergoes adaptive evolution to achieve in silico predicted optimal growth. Nature 420, 186–189.

[39] Jin, Q., Roden, E.E., Giska, J.R., 2013. Geomicrobial kinetics: Extrapolating laboratory studies to natural environments. Geomicrobiology Journal 30, 173–185.

[40] Johnson, S.G., 2014. The nlopt nonlinear-optimization package. Software.

[41] Karloff, H., 2008. Linear Programming. Modern Birkhäuser Classics, Birkhäuser Boston.

[42] Karlsson, F.H., Nookaew, I., Petranovic, D., Nielsen, J., 2011. Prospects for systems biology and modeling of the gut microbiome. Trends in Biotechnology 29, 251–258.

[43] Klee, V., Minty, G.J., 1972. How good is the simplex algorithm?, in: Inequalities, III, Academic Press, New York. pp. 159—175.

[44] Klitgord, N., Segrè, D., 2010. Environments that induce synthetic microbial ecosystems. PLOS Computational Biology 6, e1001002.

[45] Leppävuori, J.T., Domach, M.M., Biegler, L.T., 2011. Parameter estimation in batch bioreactor simulation using metabolic models: sequential solution with direct sensitivities. Industrial & Engineering Chemistry Research 50, 12080–12091.

[46] Limpert, E., Stahel, W.A., Abbt, M., 2001. Log-normal distributions across the sciences: Keys and clues. BioScience 51, 341–352.

[47] Louca, S., Doebeli, M., 2015a. Calibration and analysis of genome-based models for microbial ecology. eLife (In review).

[48] Louca, S., Doebeli, M., 2015b. Transients of competitive exclusion in microbial communities. In review .

[49] Mahadevan, R., Edwards, J.S., Doyle III, F.J., 2002. Dynamic flux balance analysis of diauxic growth in *Escherichia coli*. Biophysical Journal 83, 1331–1340.

[50] MATLAB, 2010. version 7.10.0 (R2010a). The MathWorks Inc., Natick, Massachusetts.

[51] McAdams, H.H., Arkin, A., 1998. Simulation of prokaryotic genetic circuits. Annual Review of Biophysics and Biomolecular Structure 27, 199–224.

[52] McDonald, D., Clemente, J.C., Kuczynski, J., Rideout, J.R., Stombaugh, J., Wendel, D., Wilke, A., Huse, S., Hufnagle, J., Meyer, F., et al., 2012. The biological observation matrix (biom) format or: how i learned to stop worrying and love the ome-ome. GigaScience 1, 7.

[53] Michel Berkelaar, Kjell Eikland, P.N., 2004. lpsolve 5.0 - open source (mixed-integer) linear programming system. Software.

[54] Millero, F.J., 2013. Chemical Oceanography. Marine Science Series, Taylor & Francis, Bosa Roca. 4 edition.

[55] Milne-Thomson, L., 2000. The Calculus of Finite Differences. AMS Chelsea Pub.

[56] Nelder, J.A., Mead, R., 1965. A simplex method for function minimization. The Computer Journal 7, 308–313.

[57] Orth, J.D., Thiele, I., Palsson, B.O., 2010. What is flux balance analysis? Nature Biotechnology 28, 245–248.

[58] Owens, J., Legan, J., 1987. Determination of the monod substrate saturation constant for microbial growth. FEMS Microbiology Letters 46, 419–432.

[59] Perez-Garcia, O., Villas-Boas, S., Singhal, N., 2014a. A method to calibrate metabolic network models with experimental datasets, in: Saez-Rodriguez, J., Rocha, M.P., Fdez-Riverola, F., De Paz Santana, J.F. (Eds.), 8th International Conference on Practical Applications of Computational Biology & Bioinformatics (PACBB 2014). Springer International Publishing. volume 294 of *Advances in Intelligent Systems and Computing*, pp. 183–190.

[60] Perez-Garcia, O., Villas-Boas, S.G., Swift, S., Chandran, K., Singhal, N., 2014b. Clarifying the regulation of NO/N_2O production in *Nitrosomonas europaea* during anoxic–oxic transition via flux balance analysis of a metabolic network model. Water Research 60, 267–277.

[61] Platt, T., Denman, K.L., 1975. Spectral analysis in ecology. Annual Review of Ecology and Systematics 6, 189–210.

[62] Postgate, J., 1998. Nitrogen Fixation. Cambridge University Press.

[63] Poughon, L., Dussap, C.G., Gros, J.B., 2001. Energy model and metabolic flux analysis for autotrophic nitrifiers. Biotechnology and Bioengineering 72, 416–433.

[64] Powell, M.J.D., 1994. A direct search optimization method that models the objective and constraint functions by linear interpolation, in: Gomez, S., Hennart, J.P. (Eds.), Advances in Optimization and Numerical Analysis. Kluwer Academic: Dordrecht, pp. 51–67.

[65] Powell, M.J.D., 2009. The BOBYQA algorithm for bound constrained optimization without derivatives. Technical Report NA2009/06. Department of Applied Mathematics and Theoretical Physics, Cambridge England.

[66] Press, W.H., Teukolsky, S.A., Vetterling, W.T., Flannery, B.P., 1997. Numerical recipes in C. Cambridge University Press. 2 edition.

[67] Price, N.D., Reed, J.L., Palsson, B.O., 2004. Genome-scale models of microbial cells: evaluating the consequences of constraints. Nature Reviews Microbiology 2, 886–897.

[68] Redfield, A.C., 1958. The biological control of chemical factors in the environment. American Scientist 46, 205–221.

[69] Reed, D.C., Algar, C.K., Huber, J.A., Dick, G.J., 2014. Gene-centric approach to integrating environmental genomics and biogeochemical models. Proceedings of the National Academy of Sciences 111, 1879–1884.

[70] Reed, J.L., Vo, T.D., Schilling, C.H., Palsson, B.O., et al., 2003. An expanded genome-scale model of *Escherichia coli* K-12 (iJR904 GSM/GPR). Genome Biology 4, R54.

[71] Reeve, C.A., Bockman, A.T., Matin, A., 1984. Role of protein degradation in the survival of carbon-starved *Escherichia coli* and *Salmonella typhimurium*. Journal of Bacteriology 157, 758–763.

[72] Remacle, J., De Leval, J., 1978. Approaches to nitrification in a river. in: Schlessinger, D. (Ed.), Microbiology. American Society for Microbiology. pp. 352–356.

[73] Roden, E.E., Jin, Q., 2011. Thermodynamics of microbial growth coupled to metabolism of glucose, ethanol, short-chain organic acids, and hydrogen. Applied and Environmental Microbiology 77, 1907–1909.

[74] Roling, W.F., van Bodegom, P.M., 2014. Towards quantitative understanding on microbial community structure and functioning: a modelling-centred approach using degradation of marine oil spills as example. Frontiers in Microbiology 5.

[75] Rowan, T., 1990. Functional Stability Analysis of Numerical Algorithms. Ph.D. thesis. Department of Computer Sciences, University of Texas at Austin.

[76] Royama, T., 1992. Analytical Population Dynamics. Population and Community Biology Series, Springer.

[77] Scaldaferri, M.C.L., Pimentel, A.S., 2009. Theoretical study of the reaction of hydrogen sulfide with nitrate radical. Chemical Physics Letters 470, 203 – 209.

[78] Scheibe, T.D., Mahadevan, R., Fang, Y., Garg, S., Long, P.E., Lovley, D.R., 2009. Coupling a genome-scale metabolic model with a reactive transport model to describe in situ uranium bioremediation. Microbial Biotechnology 2, 274–286.

[79] Schellenberger, J., Park, J.O., Conrad, T.M., Palsson, B.Ø., 2010. BiGG: a Biochemical Genetic and Genomic knowledgebase of large scale metabolic reconstructions a Biochemical Genetic and Genomic knowledgebase of large scale metabolic reconstructions. BMC bioinformatics 11, 213.

[80] Schellenberger, J., Que, R., Fleming, R.M.T., Thiele, I., Orth, J.D., Feist, A.M., Zielinski, D.C., Bordbar, A., Lewis, N.E., Rahmanian, S., Kang, J., Hyduke, D.R., Palsson, B.O., 2011. Quantitative prediction of cellular metabolism with constraint-based models: the COBRA Toolbox v2.0. Nature Protocols 6, 1290–1307.

[81] Schink, B., Stams, A., 2006. Syntrophism among prokaryotes, in: Dworkin, M., Falkow, S., Rosenberg, E., Schleifer, K.H., Stackebrandt, E. (Eds.), The Prokaryotes. Springer, New York, pp. 309–335.

[82] Schuetz, R., Kuepfer, L., Sauer, U., 2007. Systematic evaluation of objective functions for predicting intracellular fluxes in *Escherichia coli*. Molecular Systems Biology 3.

[83] Segrè, D., Vitkup, D., Church, G.M., 2002. Analysis of optimality in natural and perturbed metabolic networks. Proceedings of the National Academy of Sciences 99, 15112–15117.

[84] Shapiro, O.H., Kushmaro, A., 2011. Bacteriophage ecology in environmental biotechnology processes. Current Opinion in Biotechnology 22, 449–455.

[85] Starkenburg, S.R., Chain, P.S.G., Sayavedra-Soto, L.A., Hauser, L., Land, M.L., Larimer, F.W., Malfatti, S.A., Klotz, M.G., Bottomley, P.J., Arp, D.J., Hickey, W.J., 2006. Genome sequence of the chemolithoautotrophic nitrite-oxidizing bacterium *Nitrobacter winogradskyi* nb-255. Applied and Environmental Microbiology 72, 2050–2063.

[86] Stolper, D.A., Revsbech, N.P., Canfield, D.E., 2010. Aerobic growth at nanomolar oxygen concentrations. Proceedings of the National Academy of Sciences 107, 18755–18760.

[87] Stolyar, S., Van Dien, S., Hillesland, K.L., Pinel, N., Lie, T.J., Leigh, J.A., Stahl, D.A., 2007. Metabolic modeling of a mutualistic microbial community. Molecular Systems Biology 3, 92.

[88] Suttle, C.A., 2007. Marine viruses – major players in the global ecosystem. Nature Reviews Microbiology 5, 801–812.

[89] Suzuki, I., Dular, U., Kwok, S.C., 1974. Ammonia or ammonium ion as substrate for oxidation by *Nitrosomonas europaea* cells and extracts. Journal of Bacteriology 120, 556–558.

[90] Tarantola, A., 2005. Inverse Problem Theory and Methods for Model Parameter Estimation. Society for Industrial and Applied Mathematics, Philadelphia.

[91] Uhlenbeck, G.E., Ornstein, L.S., 1930. On the theory of the Brownian motion. Physical Review 36, 823–841.

[92] Varma, A., Palsson, B.O., 1993. Metabolic capabilities of *Escherichia coli*: I. Synthesis of biosynthetic precursors and cofactors. Journal of Theoretical Biology 165, 477–502.

[93] Varma, A., Palsson, B.O., 1994. Stoichiometric flux balance models quantitatively predict growth and metabolic by-product secretion in wild-type *Escherichia coli* W3110. Applied and Environmental Microbiology 60, 3724–3731.

[94] Villéger, A.C., Pettifer, S.R., Kell, D.B., 2010. Arcadia: a visualization tool for metabolic pathways. Bioinformatics 26, 1470–1471.

[95] Voss, M., Bange, H.W., Dippner, J.W., Middelburg, J.J., Montoya, J.P., Ward, B., 2013. The marine nitrogen cycle: recent discoveries, uncertainties and the potential relevance of climate change. Philosophical Transactions of the Royal Society B: Biological Sciences 368.

[96] Williams, T., Kelley, C., many others, 2010. Gnuplot 4.4: an interactive plotting program. http://gnuplot.sourceforge.net.

[97] Zhuang, K., Izallalen, M., Mouser, P., Richter, H., Risso, C., Mahadevan, R., Lovley, D.R., 2011. Genome-scale dynamic modeling of the competition between *Rhodoferax* and *Geobacter* in anoxic subsurface environments. The ISME journal 5, 305–316.
