# Supplementary material for: Calibration and analysis of genome-based models for microbial ecology
Source: eLife. 2015 Oct 16;4:e08208. doi: 10.7554/eLife.08208 (PMC4608356; doi:10.7554/eLife.08208)
Supplement: Supplementary file 2. — An overview of the fitted parameter values for the E. coli models. DOI: http://dx.doi.org/10.7554/eLife.08208.014 [file elife08208s002.pdf]

Supplementary file 2: Fitted parameters for the *E. coli* models described in the main article, together with reference values from the literature for comparison. Maximum cell-specific uptake rates (V_{max}) are in fmol/cell/d. Half-saturation constants for acetate ($K_{\mathrm{half,acetate}}$) are in mM, half-saturation constants for glucose ($K_{\mathrm{half,glucose}}$) are in $\mu\mathrm{M}$. Initial cell densities are in 10^9 $\mathrm{cells/L}$. Non-growth associated maintenance requirements are given in $\mathrm{fmol\ ATP/cell/d}$. The $\mathrm{O_2}$ mass transfer coefficient is in 1/d (reference value only roughly comparable, as the transfer coefficient depends strongly on shaking frequency and flask volume (Maier *et al.*, 2004)). Dry-weight-specific values from the literature were converted to cell-specific values by assuming a dry weight of 180 $\mathrm{fg/cell}$ (Fagerbakke *et al.*, 1996). All reference values were measured for strains other than B REL606.

**parameter**	**values**	**comparison**	**reference**
$V_{\mathrm{max,acetate}}$	67.8 (A), 16.5 (SS), 220 (FS)	8.6	(Meadows *et al.*, 2010)
$K_{\mathrm{half,acetate}}$	10.6 (A), 5.55 (SS), 12.9 (FS)	6.0	(Meadows *et al.*, 2010)
$V_{\mathrm{max,glucose}}$	43.2 (A), 56.9 (SS), 29.0 (FS)	45	(Varma & Palsson, 1994)
$K_{\mathrm{half,glucose}}$	21.3 (A), 11.4 (SS), 44.6 (FS)	3–15	(Gosset, 2005)
maintenance req.	18.6 (A), 11.0 (SS), 15.0 (FS)	32	(Varma & Palsson, 1994)
$\mathrm{O_2}$ mass transfer	60.9	180	(Mahadevan *et al.*, 2002)
init. cell density	8.48 (A), 11.3 (SS), 7.57 (FS)		

References

Fagerbakke K, Heldal M, Norland S (1996) Content of carbon, nitrogen, oxygen, sulfur and phosphorus in native aquatic and cultured bacteria. *Aquatic Microbial Ecology* 10: 15–27

Gosset G (2005) Improvement of *Escherichia coli* production strains by modification of the phosphoenolpyruvate: sugar phosphotransferase system. *Microbial Cell Factories* 4: 14

Mahadevan R, Edwards JS, Doyle III FJ (2002) Dynamic flux balance analysis of diauxic growth in *Escherichia coli*. *Biophysical Journal* 83: 1331–1340

Maier U, Losen M, Büchs J (2004) Advances in understanding and modeling the gas–liquid mass transfer in shake flasks. *Biochemical Engineering Journal* 17: 155–167

Meadows AL, Karnik R, Lam H, Forestell S, Snedecor B (2010) Application of dynamic flux balance analysis to an industrial *Escherichia coli* fermentation. *Metabolic Engineering* 12: 150–160

Varma A, Palsson BO (1994) Stoichiometric flux balance models quantitatively predict growth and metabolic by-product secretion in wild-type *Escherichia coli* W3110. *Applied and Environmental Microbiology* 60: 3724–3731
